# Phenome-wide association studies demonstrating pleiotropy of genetic variants within *FTO* with and without adjustment for body mass index

**DOI:** 10.3389/fgene.2014.00250

**Published:** 2014-08-05

**Authors:** Robert M. Cronin, Julie R. Field, Yuki Bradford, Christian M. Shaffer, Robert J. Carroll, Jonathan D. Mosley, Lisa Bastarache, Todd L. Edwards, Scott J. Hebbring, Simon Lin, Lucia A. Hindorff, Paul K. Crane, Sarah A. Pendergrass, Marylyn D. Ritchie, Dana C. Crawford, Jyotishman Pathak, Suzette J. Bielinski, David S. Carrell, David R. Crosslin, David H. Ledbetter, David J. Carey, Gerard Tromp, Marc S. Williams, Eric B. Larson, Gail P. Jarvik, Peggy L. Peissig, Murray H. Brilliant, Catherine A. McCarty, Christopher G. Chute, Iftikhar J. Kullo, Erwin Bottinger, Rex Chisholm, Maureen E. Smith, Dan M. Roden, Joshua C. Denny

**Affiliations:** ^1^Department of Medicine, Vanderbilt UniversityNashville, TN, USA; ^2^Department of Biomedical Informatics, Vanderbilt UniversityNashville, TN, USA; ^3^Office of Research, Vanderbilt UniversityNashville, TN, USA; ^4^Department of Molecular Physiology and Biophysics, Center for Human Genetics Research, Vanderbilt UniversityNashville, TN, USA; ^5^Department of Pharmacology, Vanderbilt UniversityNashville, TN, USA; ^6^Vanderbilt Epidemiology Center, Vanderbilt UniversityNashville, TN, USA; ^7^Center for Human Genetics, Marshfield Clinic Research FoundationMarshfield, WI, USA; ^8^Biomedical Informatics Research Center, Marshfield Clinic Research FoundationMarshfield, WI, USA; ^9^Division of Genomic Medicine, National Human Genome Research InstituteBethesda, MD, USA; ^10^Department of Medicine, University of WashingtonSeattle, WA, USA; ^11^Department of Biochemistry and Molecular Biology, Center for Systems Genomics, The Pennsylvania State UniversityUniversity Park, PA, USA; ^12^Divisions of Biomedical Informatics and Statistics, Mayo ClinicRochester, MN, USA; ^13^Division of Epidemiology, Mayo ClinicRochester, MN, USA; ^14^Group Health Research InstituteSeattle, WA, USA; ^15^Department of Genome Sciences, University of WashingtonSeattle, WA, USA; ^16^Genomic Medicine Institute, Geisinger Health SystemDanville, PA, USA; ^17^Weis Center for Research, Geisinger Health SystemDanville, PA, USA; ^18^Essentia Institute of Rural HealthDuluth, MN, USA; ^19^Division of Cardiovascular Diseases, Mayo ClinicRochester, MN, USA; ^20^The Charles Bronfman Institute for Personalized Medicine, Icahn School of Medicine at Mount SinaiNew York, NY, USA; ^21^Department of Cell and Molecular Biology, Feinberg School of Medicine, Northwestern UniversityEvanston, IL, USA

**Keywords:** PheWAS, genetic association, pleiotropy, Exome chip, *FTO*, BMI

## Abstract

Phenome-wide association studies (PheWAS) have demonstrated utility in validating genetic associations derived from traditional genetic studies as well as identifying novel genetic associations. Here we used an electronic health record (EHR)-based PheWAS to explore pleiotropy of genetic variants in the fat mass and obesity associated gene (*FTO*), some of which have been previously associated with obesity and type 2 diabetes (T2D). We used a population of 10,487 individuals of European ancestry with genome-wide genotyping from the Electronic Medical Records and Genomics (eMERGE) Network and another population of 13,711 individuals of European ancestry from the BioVU DNA biobank at Vanderbilt genotyped using Illumina HumanExome BeadChip. A meta-analysis of the two study populations replicated the well-described associations between *FTO* variants and obesity (odds ratio [OR] = 1.25, 95% Confidence Interval = 1.11–1.24, *p* = 2.10 × 10^−9^) and *FTO* variants and T2D (*OR* = 1.14, 95% *CI* = 1.08–1.21, *p* = 2.34 × 10^−6^). The meta-analysis also demonstrated that *FTO* variant rs8050136 was significantly associated with sleep apnea (*OR* = 1.14, 95% *CI* = 1.07–1.22, *p* = 3.33 × 10^−5^); however, the association was attenuated after adjustment for body mass index (BMI). Novel phenotype associations with obesity-associated *FTO* variants included fibrocystic breast disease (rs9941349, *OR* = 0.81, 95% *CI* = 0.74–0.91, *p* = 5.41 × 10^−5^) and trends toward associations with non-alcoholic liver disease and gram-positive bacterial infections. *FTO* variants not associated with obesity demonstrated other potential disease associations including non-inflammatory disorders of the cervix and chronic periodontitis. These results suggest that genetic variants in *FTO* may have pleiotropic associations, some of which are not mediated by obesity.

## Introduction

Pleiotropy, the phenomenon in which a single gene or genetic variant is associated with multiple phenotypes, is essential to the functionality of the human genome (Crespi, 2010; Wagner and Zhang, 2011; Pavlicev and Wagner, 2012). Through comparing multiple genome-wide association studies (GWAS) and candidate gene studies, pleiotropy has been noted in many single nucleotide polymorphisms (SNPs) and genes, potentially providing greater insight into putative biological mechanisms (Sivakumaran et al., 2011; Stranger et al., 2011; Solovieff et al., 2013). The increasing prevalence of DNA biobanks linked to rich phenotype resources and large epidemiological databases have enabled the development of phenome-wide association study (PheWAS) method as an additional tool to investigate pleiotropy (Denny et al., 2010a; Pendergrass et al., 2011). As a complement to GWAS, PheWAS enables both the validation of genotype-phenotype associations identified through traditional GWAS and the generation of new hypotheses, identifying potentially novel associations as well as putative instances of genetic pleiotropy (Denny et al., 2011; Pendergrass et al., 2013). A recent application of PheWAS to 3144 GWAS-identified variants, replicated 210 known associations and noted 63 new, pleiotropic associations (Denny et al., 2013).

The Electronic Medical Records and Genomics (eMERGE) Network was formed in 2007 to use phenotypes derived from electronic health record (EHR) data to perform GWAS and other genomic investigations (Kullo et al., 2011; Pathak et al., 2011; Crosslin et al., 2013; Ding et al., 2013). eMERGE investigators have also used EHR-based PheWAS methods to evaluate multiple phenotypes associated with specific genetic variants (Denny et al., 2010a; Pathak et al., 2012; Hebbring et al., 2013). PheWAS has been used to enhance our understanding of the genetic determinants of complex traits discovered through GWAS. For example, a PheWAS of variants associated with longer cardiac conduction (Ritchie et al., 2013) demonstrated an association with atrial fibrillation, and a PheWAS of variants affecting platelet count and size identified associations with autoimmune diseases (Shameer et al., 2013).

Variants in the fat mass and obesity associated gene (*FTO)* have been studied since 2007, when it was discovered that some were associated with body mass index (BMI) and obesity (Frayling et al., 2007). Multiple GWAS have demonstrated further associations between variants in *FTO* and obesity (Jacobsson et al., 2012). Some of these variants have also been noted to be associated with both obesity and type 2 diabetes (T2D) (Hertel et al., 2011; Rees et al., 2011; Li et al., 2012) including SNPs rs9939609 and rs8050136, which are in high linkage disequilibrium (LD) with each other in people of European ancestry (*r*^2^ = 1.00; using 1000 Genomes Pilot 1 reference in the CEU population). The SNP rs8050136 is located in an intronic region where the transcription factor cut-like homeobox (*CUTL1*) protein (Li et al., 2000) is predicted to bind (Stratigopoulos et al., 2008). This variant has been associated with T2D and obesity in Han Chinese and European populations (Hubacek et al., 2008; Liu et al., 2010; Hotta et al., 2011) but other studies found no association between this variant and T2D or obesity in the Chinese Han population (Li et al., 2008; Xi and Mi, 2009). These differences in associations of SNPs with phenotypes have been further analyzed through fine mapping of BMI loci (Gong et al., 2013). This study reported that GWAS studies primarily performed in European populations of numerous loci associated with BMI are not generalizable to other ethnic groups, for example African Americans. Another study demonstrated that rs8050136 was associated with increased energy intake from fat with similar total energy intake (Park et al., 2013). A more recent study noted that the mechanism of action for common variants in *FTO* may be through regulation of *IRX3* expression, which is highly expressed in the brain (Smemo et al., 2014).

There is also evidence of other putative disease associations with *FTO* variants that have not achieved genome-wide significance, such as pancreatic cancer, Alzheimer's disease, attention deficit hyperactivity disorder, alcoholism, and osteoarthritis (Keller et al., 2011; Lurie et al., 2011; Sobczyk-Kopciol et al., 2011; arcOGEN Consortium et al., 2012; Corella et al., 2012; Reitz et al., 2012; Velders et al., 2012). These varied disease-SNP associations suggest that SNPs in *FTO* may have pleiotropic effects. Utilizing the population and diagnostic diversity contained within the real-world clinical environment for variants within *FTO*, our goal was to determine whether an EHR-based PheWAS could identify genetic pleiotropy that might otherwise remain undetected in traditional cohort study designs. In the present study, we utilized PheWAS method and data sets from the eMERGE network (McCarty et al., 2011; Gottesman et al., 2013) to evaluate pleiotropy of variants in *FTO*.

## Materials and methods

### Participation of eMERGE sites

The eMERGE Network data used in this study consists of seven institutions (Group Health Cooperative and University of Washington, Marshfield Clinic, Mayo Clinic, Northwestern University, Mount Sinai, Geisinger Health System, and Vanderbilt University Medical Center), each with DNA biorepositories linked to their EHRs. Each site pulled demographic, vital sign, and billing data from their EHR research data repositories for this study. All projects were either approved by local IRBs or classified as IRB exempt as non-human subjects research.

### Genotyping of eMERGE subjects

Variants for eMERGE subjects were selected from extant genome-wide genotypes with either the Human660W-Quadv1_A or Illumina OmniExpress chips. The Human660W-Quadv1_A BeadChip was completed at the Center for Genotyping and Analysis at the Broad Institute, and the Center for Inherited Disease Research at Johns Hopkins University. Genotyping for Illumina OmniExpress BeadChips was performed at the University of Pittsburgh Genomics and Proteomics Core Laboratories. These genotyping data comprised 10,487 individuals of European ancestry, as designated in the EHRs.

Quality-control (QC) of the genotype data was performed using a pipeline developed by the eMERGE Genomics Working Group (Turner et al., 2011). This process included call rate restrictions listed below, identification of sex mismatch and anomalies, checking duplicate and HapMap concordance, as well as identifying batch effects, sample relatedness, and minor allele frequency (MAF). Population stratification was evaluated using STRUCTURE (Pritchard et al., 2000) and EIGENSTRAT (Price et al., 2006). Only SNPs with call rates >99% and MAF >0.01 in unrelated samples were included for further study. Relatedness was determined on the basis of identity by descent (IBD) estimates generated from the genome-wide genotype data in PLINK (Purcell et al., 2007). All study sites had pairs of individuals with an IBD estimate greater than 0.25; only one of the individuals in each related pair was randomly selected and used in the analysis. Additional genotypes were imputed using IMPUTE2 (Marchini et al., 2007) and 1000 Genomes Project as the reference (1000 Genomes Project Consortium et al., 2010). We used imputed SNPs with a minimum info score of 0.7 and called genotypes based on the maximum posterior probability.

We selected 54 SNPs, of which 51 were imputed in at least one site, located in *FTO* that met the QC criteria above and were previously associated with obesity (Jacobsson et al., 2012). QC and subsequent association tests were performed using PLINK (Purcell et al., 2007) and the R statistical package (R Core Team, 2013).

### Genotyping of vanderbilt subjects using HumanExome BeadChips

We selected 13,711 individuals of European ancestry from the BioVU DNA databank with BMI data who were genotyped using the Illumina Infinium HumanExome BeadChip, which includes >240,000 markers, mostly within exonic regions, as well as SNPs from the GWAS catalog (Welter et al., 2014) including rs8050136 in *FTO*. Genotyping was performed at the Vanderbilt Technologies for Advanced Genomics (VANTAGE) Core, and genomic data were processed by the Vanderbilt Technologies for Advanced Genomics Analysis and Research Design (VANGARD) Core. Clustering was performed using GenomeStudio's GenTrain clustering algorithm followed by manual review and reclustering; genotype calling was performed using GenomeStudio's GenCall algorithm. Genotyping quality was evaluated using SNP call rates and concordance rates with HapMap controls; SNPs with <99.8% call rate or <98% concordance were excluded. In the first analysis, we focused on rs8050136, which had a call rate of >99.9%. In the subsequent analyses, we further analyzed eight *FTO* SNPs on the Exome chip, which had call rates greater than 99.8% and MAFs >0.01. Similar to the eMERGE set, for individuals with an IBD estimate greater than 0.25; only one of the individuals in each related group was selected randomly and used in our analyses.

### PheWAS analyses

We first tested the 54 eMERGE SNPs for association with BMI using linear regression. We calculated LD with our reference SNP rs8050136, chosen as the reference because of its GWAS associations with BMI and T2D in the literature and since it was directly genotyped on all of the platforms. To evaluate phenotype associations and potential pleiotropy among different FTO SNPs, we grouped SNPs into three groups for convenience based on their LD with rs805136: high LD (*r*^2^ > 0.80), moderate LD (0.80 ≥ *r*^2^ > 0.60) and low LD (*r*^2^ ≤ 0.60) with rs8050136. Our hypothesis was that SNPs in high LD would show similar patterns of phenotype associations with rs8050136, and that different patterns may be observed in SNPs with lower LD.

Analyses for the eMERGE and the BioVU datasets were conducted separately and then meta-analyzed. The eMERGE population had 54 SNPs and the BioVU population had nine SNPs for analysis, which were also present in the eMERGE dataset. We used logistic regression adjusted for age, sex, eMERGE site, and the first three principal components as calculated for each dataset by EIGENSTRAT, using an additive genetic model. We performed PheWAS using each SNP using methods and code groupings described previously (Denny et al., 2013) using the R PheWAS package (Carroll et al., 2014), briefly, calculating comprehensive associations between SNPs and a total of 1645 clinical phenotypes derived from the International Classification of Disease, 9th CM (ICD-9) edition codes from each site's EHR. The ICD-9 codes that are associated with each phenotype can be found at the PheWAS catalog located online at http://phewas.mc.vanderbilt.edu/. Cases for a given disease were defined as having at least two relevant ICD-9 codes on different days. The PheWAS method also defines control groups for each disease, which ensures that related diseases do not serve as controls for the current disease being analyzed. We performed association testing for all PheWAS phenotypes occurring in at least 20 individuals (effectively 20 “cases”).

We then compared our results to performing PheWAS for each *FTO* SNP adjusting for BMI. The BMI, obtained from each site's EHR, was estimated using the average BMI from individuals within our dataset. To minimize erroneous data, we only used BMI measurements between 15 and 70, a range that we have used in prior studies and has good precision (Denny et al., 2010b). Plotting was performed in R using the PheWAS and ggplot2 packages.

Meta-analysis was performed using the inverse-variance method (Hunter et al., 1982) for the nine shared SNPs. There were 1010 phenotypes that were in common across both datasets and met our minimum case criteria of at least 20 cases. This yields a Bonferroni corrected *p*-value of 4.95 × 10^−5^, (*p* = 0.05/1010 = 4.95 × 10^−5^), for a single SNP. We chose a single SNP, phenome-wide correction threshold since most of the SNPs in this analysis were in high LD with each other and thus do not represent truly independent tests. A false discovery rate (FDR) of *q* = 0.05, calculated with the Benjamin and Hochberg method using the R p.adjust method, yields a *p*-value of 2.48 × 10^−4^ (Benjamini and Hochberg, 1995). For our latter analyses, we considered a total of 54 SNPs. Since many phenotypes are correlated with each other and many of the SNPs are in LD, we also used simpleM (Gao et al., 2010) to estimate the number of unique tests performed, leading to an adjustment of *p* = 2.36 × 10^−6^. All analyses assumed a two-tailed distribution.

## Results

### Overview

A total of 24,198 individuals were used in our analyses (Table 1). Both the eMERGE and BioVU datasets were similar in median age, sex, and BMI. Our analysis of the association of the *FTO* SNPs with BMI (Table 2) showed that most SNPs in high linkage disequilibrium with rs8050136 (*r*^2^ > 0.80) have highly significant *p*-values (<3 × 10^−9^) and betas for BMI (Table 2). SNPs with lower correlations with rs8050136 have highly variable associations with BMI.

**Table 1:** **Characteristics of the study sets**.

	**eMERGE *n* = 10,487**	**BioVU *n* = 13,711**	**Combined *n* = 24,198**
Genotyping Platform	Illumina Human660W-Quadv1_A	Illumina HumanExome	
Number of SNPs	54	9	9
Total number of phenotypes	1094	1254	1010
Median age (IQR)	58 (48–68)	60 (47–72)	59 (48–70)
Female (%)	52.24	54.31	53.35
BMI (average ± *SD*)	30.86 ± 7.48	28.43 ± 6.44	29.54 ± 7.04
Most frequent diagnoses	Hypertension (66%) Hyperlipidemia (61%) Pain in limb (47%) Malaise and fatigue (39%) Abdominal/pelvic symptoms (36%)	Hypertension (63%) Malaise and fatigue (51%) Eye infection, viral (50%) Hyperlipidemia (40%) Pain in limb (39%)	Hypertension (64%) Hyperlipidemia (49%) Malaise and fatigue (46%) Pain in limb (43%) GERD (34%)

*This table shows the main characteristics of the study populations of European ancestry, including age, sex, BMI and the five most significant PheWAS phenotypes observed in the datasets. The sample size included 10,487 from the eMERGE population and 13,711 from the BioVU population for a total of 24,198 people. For a given phenotype, in the combined dataset our maximum number of cases was 14,592 in hypertension and the minimum number of cases was 44*.

**Table 2:** **Association between *FTO* variants and average BMI**.

**SNP**	**Minor allele**	**Major allele**	**MAF**	**Beta (95%CI)**	***p*^†^**	***r*^2^**
**rs8050136**	**A**	**C**	**0.41**	**0.535 (0.363, 0.707)**	**1.28E-09**	**1.00**
rs9935401	A	G	0.41	0.535 (0.363, 0.707)	1.26E-09	1.00
rs11075990	G	A	0.42	0.534 (0.362, 0.706)	1.29E-09	1.00
rs9923233	C	G	0.42	0.534 (0.362, 0.706)	1.29E-09	1.00
rs9926289	A	G	0.42	0.534 (0.362, 0.706)	1.29E-09	1.00
rs9936385	C	T	0.42	0.534 (0.362, 0.706)	1.29E-09	1.00
rs9939609	A	T	0.42	0.534 (0.362, 0.706)	1.29E-09	1.00
rs8043757	T	A	0.41	0.539 (0.367, 0.711)	9.71E-10	1.00
rs7185735	G	A	0.42	0.536 (0.364, 0.708)	1.17E-09	1.00
rs17817449	G	T	0.40	0.548 (0.376, 0.720)	5.07E-10	1.00
rs7193144	C	T	0.41	0.529 (0.357, 0.701)	1.96E-09	1.00
rs3751812	T	G	0.34	0.572 (0.400, 0.744)	9.34E-11	0.99
rs55872725	T	C	0.35	0.561 (0.389, 0.733)	1.67E-10	0.94
rs1558902	A	T	0.35	0.560 (0.388, 0.732)	1.84E-10	0.94
rs62048402	A	G	0.35	0.560 (0.388, 0.732)	1.84E-10	0.94
rs11642015	T	C	0.35	0.561 (0.389, 0.733)	1.70E-10	0.94
rs1421085	C	T	0.35	0.561 (0.389, 0.733)	1.70E-10	0.94
rs9941349	T	C	0.37	0.564 (0.392, 0.736)	1.42E-10	0.92
rs9931494	G	C	0.37	0.561 (0.389, 0.733)	1.72E-10	0.92
rs12149832	A	G	0.35	0.560 (0.388, 0.732)	1.71E-10	0.90
rs1121980	A	G	0.44	0.522 (0.351, 0.693)	2.40E-09	0.88
rs9939973	A	G	0.43	0.528 (0.357, 0.699)	1.48E-09	0.88
rs9940646	G	C	0.43	0.528 (0.357, 0.699)	1.48E-09	0.88
rs9940128	A	G	0.43	0.527 (0.356, 0.698)	1.61E-09	0.88
rs9937053	A	G	0.43	0.530 (0.359, 0.701)	1.35E-09	0.88
rs9930333	G	T	0.44	0.534 (0.363, 0.705)	9.67E-10	0.88
rs9932754	C	T	0.39	0.544 (0.373, 0.715)	4.63E-10	0.85
rs9930506	G	A	0.39	0.544 (0.373, 0.715)	4.63E-10	0.85
rs9922619	T	G	0.39	0.553 (0.382, 0.724)	2.37E-10	0.85
rs8057044	G	A	0.47	0.530 (0.359, 0.701)	1.25E-09	0.72
rs17817288	G	A	0.48	0.528 (0.357, 0.699)	1.19E-09	0.68
rs9922047	C	G	0.44	0.502 (0.331, 0.673)	7.21E-09	0.64
rs1861866	C	T	0.44	0.498 (0.327, 0.669)	9.63E-09	0.64
rs8055197	G	A	0.44	0.498 (0.327, 0.669)	9.63E-09	0.64
rs10852521	T	C	0.44	0.497 (0.326, 0.668)	1.02E-08	0.64
rs8047395	G	A	0.43	0.496 (0.325, 0.667)	1.10E-08	0.64
rs8044769	T	C	0.42	0.504 (0.333, 0.675)	6.64E-09	0.62
rs3751813	G	T	0.45	0.419 (0.247, 0.591)	2.06E-06	0.57
rs4783819	G	C	0.33	0.414 (0.236, 0.592)	5.43E-06	0.41
rs1477196	A	G	0.32	0.410 (0.232, 0.588)	6.74E-06	0.40
rs7190492	A	G	0.33	0.426 (0.248, 0.604)	2.83E-06	0.40
rs7186521	G	A	0.45	0.251 (0.080, 0.422)	3.79E-03	0.09
rs1861869	G	C	0.47	0.274 (0.103, 0.445)	1.62E-03	0.08
rs1861868	T	C	0.44	0.256 (0.087, 0.425)	3.04E-03	0.08
rs6499640	G	A	0.39	0.264 (0.090, 0.438)	3.15E-03	0.06
rs11075986	G	C	0.12	0.065 (-0.251, 0.381)	0.69	0.06
rs16945088	G	A	0.12	0.001 (-0.317, 0.319)	0.99	0.06
rs8063946	T	C	0.12	0.101 (-0.260, 0.462)	0.58	0.04
rs1075440	G	A	0.28	0.173 (-0.011, 0.357)	0.06	0.04
rs16952520	G	A	0.09	0.205 (-0.238, 0.648)	0.36	0.03
rs12447107	C	G	0.08	0.246 (-0.379, 0.871)	0.44	0.01
rs7204609	C	T	0.10	0.469 (-0.111, 1.049)	0.11	0.01
rs7199182	G	A	0.06	2.346 (0.472, 4.220)	0.01	0.00
rs1108102	A	T	0.03	1.045 (-1.732, 3.822)	0.46	0.00

*Analysis used an additive genetic model and linear regression adjusted for age, sex, and first three principal components using the imputed eMERGE samples. The SNPs below are sorted by p-value. The beta represents the kg/m^2^ increase in BMI per minor allele. Linkage disequilibrium (r^2^) was calculated between rs8050136 (bolded) and other FTO SNPs using the eMERGE imputed set. The Bonferroni correction alpha = 0.05 for 54 SNPs is 9.26 × 10^−3^*.

† *Values are not corrected for multiple testing*.

### PheWAS of *FTO* rs8050136 unadjusted for BMI

In the BioVU population, we observed that obesity (*OR* = 1.22, *p* = 1.4 × 10^−6^, 95% *CI* = 1.13–1.33) was significantly associated with rs8050136. Three obesity-related diseases also trended toward significance; T2D (*OR* = 1.14, *p* = 5.3 × 10^−5^, 95% *CI* = 1.07–1.21), obstructive sleep apnea (OSA; *OR* = 1.15, *p* = 4.6 × 10^−3^, 95% *CI* = 1.04–1.26) and chronic non-alcoholic liver disease (NAFLD; *OR* = 1.20, *p* = 6.06 × 10^−3^, 95% *CI* = 1.05–1.38) (Supplementary Table 1). We observed similar odds ratios for obesity and T2D in eMERGE (obesity: *OR* = 1.37, *p* = 1.88 × 10^−4^, 95% *CI* = 1.16–1.61; T2D: *OR* = 1.16, *p* = 0.014, 95% *CI* = 1.03–1.32). eMERGE results also demonstrated similar trends toward significant associations with OSA (*OR* = 1.14, *p* = 2.4 × 10^−3^, 95% *CI* = 1.05–1.24) (Supplementary Table 1). After meta-analysis, obesity (*OR* = 1.25, *p* = 2.1 × 10^−9^, 95% *CI* = 1.16–1.35), morbid obesity (*OR* = 1.34, *p* = 1.07 × 10^−7^, 95% *CI* = 1.20–1.48), and two obesity-related diseases, T2D (*OR* = 1.14, *p* = 2.3 × 10^−6^, 95% *CI* = 1.08–1.21) and OSA (*OR* = 1.15, *p* = 3.3 × 10^−5^, 95% *CI* = 1.07–1.22), were associated with rs8050136 (Table 3). Additionally, the associations with NAFLD and fibrocystic breast disease were also *q* < 0.05.

**Table 3:** **Meta-analysis PheWAS results for rs8050136 with and without adjustment for average BMI**.

**Phenotype**	**Cases**	**Not adjusted for BMI**		**Adjusted for BMI**	
		***p*^†^**	***OR* (95% CI)**	***p*^†^**	***OR* (95% *CI*)**
Overweight	3943	1.38 × 10^−8^	1.17 (1.11–1.24)	0.185	1.05 (0.98–1.12)
Obesity	1662	2.10 × 10^−9^	1.25 (1.16–1.35)	0.017	1.11 (1.02–1.22)
Morbid obesity	756	1.07 × 10^−7^	1.34 (1.20–1.48)	0.016	1.17 (1.03–1.33)
Type 2 diabetes	3936	2.34 × 10^−6^	1.14 (1.08–1.21)	4.56 × 10^−4^	1.09 (1.03–1.15)
Sleep apnea	2335	3.33 × 10^−5^	1.14 (1.07–1.22)	0.040	1.07 (1.00–1.15)
Cystic mastopathy	967	2.00 × 10^−4^	0.82 (0.74–0.91)	4.75 × 10^−4^	0.84 (0.75–0.92)
Chronic Nonalcoholic Liver disease	684	2.22 × 10^−4^	1.23 (1.10–1.37)	1.86 × 10^−3^	1.19 (1.07–1.33)
Chronic Ulcer of Leg or Foot	768	8.31 × 10^−4^	1.19 (1.08–1.32)	2.55 × 10^−3^	1.17 (1.06–1.30)
Acute Renal Failure	2047	1.12 × 10^−3^	1.12 (1.05–1.20)	3.74 × 10^−3^	1.11 (1.03–1.19)
Staphylococcus infections	723	2.44 × 10^−3^	1.18 (1.06–1.31)	5.76 × 10^−3^	1.16 (1.04–1.29)
Superficial cellulitis and abscess	2861	5.65 × 10^−3^	1.09 (1.02–1.15)	0.039	1.06 (1.00–1.13)
Streptococcus infection	428	4.26 × 10^−3^	1.21 (1.05–1.39)	6.56 × 10^−3^	1.21 (1.05–1.39)
Osteomyelitis	352	6.15 × 10^−3^	1.23 (1.06–1.43)	0.011	1.21 (1.04–1.41)
All gram positive infections	1095	6.21 × 10^−4^	1.16 (1.07–1.27)	1.3 × 10^−3^	1.15 (1.06–1.26)
Joint effusions	387	2.35 × 10^−3^	1.25 (1.08–1.44)	6.90 × 10^−3^	1.22 (1.06–1.41)

*This table includes all phenotypes with p-value less than 1.00 × 10^−4^ prior to BMI adjustment. The Bonferroni alpha = 0.05 equates to a p-value of 4.95 × 10^−5^, and an FDR of q = 0.05 gives a p-value of 2.48 × 10^−4^. OR, Odds ratio; CI, confidence interval. The ICD-9 codes that are associated with each phenotype can be found at the PheWAS catalog located online at http://phewas.mc.vanderbilt.edu/*.

† *Values are not corrected for multiple testing*.

### PheWAS of *FTO* rs8050136 adjusted for BMI

**Figure 1:**
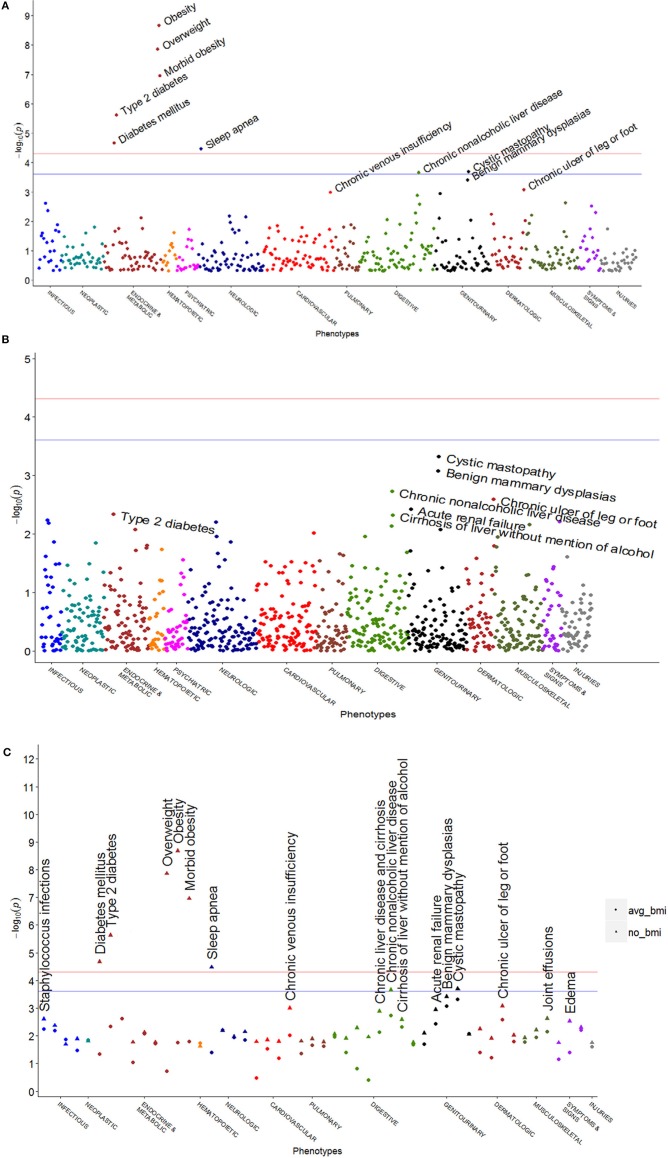
**PheWAS plots for *FTO* rs8050136 with and without BMI adjustment**. The pink horizontal line represents *p* = 4.95 × 10^−5^, which is the Bonferroni correction, and the blue horizontal line represents an FDR *q* = 0.05 (*p* = 2.48 × 10^−4^). **(A)** without BMI adjustment, **(B)** with BMI adjustment, and **(C)** most significant phenotypic associations before and after BMI adjustment (BMI-unadjusted values are shown as triangles and average BMI values are shown as dots) plotted on the same axis. The colors of points indicate the membership according to the phenotype classes identified on the X axis.

After adjusting for average BMI, some of the associations were greatly attenuated, while others remained relatively unchanged (Table 3, Figure 1). The associations with obesity and OSA were largely attenuated by adjustment for BMI (obesity: *OR* = 1.11, *p* = 0.017, 95% *CI* = 1.02–1.22; morbid obesity: *OR* = 1.17, *p* = 0.016, 95% *CI* = 1.03–1.33; OSA: *OR* = 1.07, *p* = 0.040, 95% *CI* = 1.00–1.15). Chronic non-alcoholic liver disease demonstrated a possible association with rs8050136, which was only slightly attenuated between unadjusted and BMI-adjusted analyses (*OR*: 1.23 vs. 1.19; *p*: 2.2 × 10^−4^ vs. 1.9 × 10^−3^, 95% *CI* = 1.10–1.37 vs. 1.07–1.33). Additional phenotypes trended toward association with rs8050136, including fibrocystic breast disease (*OR* = 0.84, *p* = 4.8 × 10^−4^, 95% *CI* = 0.75–0.92), staphylococcal infections (*OR* = 1.16, *p* = 5.8 × 10^−3^, 95% *CI* = 1.04–1.29), streptococcal infections (*OR* = 1.21, *p* = 6.6 × 10^−3^, 95% *CI* = 1.05–1.39), osteomyelitis (*OR* = 1.21, *p* = 0.011, 95% *CI* = 1.04–1.41), and joint effusions (*OR* = 1.22, *p* = 6.9 × 10^−3^, 95% *CI* = 1.06–1.41). These were not notably changed by BMI adjustment. Due to the number of gram-positive bacterial infections, we tested *post hoc* for the association between the SNP and a composite phenotype of all gram-positive infections, which were defined as staphylococcal infections, streptococcal infections, pneumococcal pneumonia, and gram positive septicemia. When combining all gram-positive phenotypes, the result was similar to the individual phenotypes (*n* = 1095, *OR* = 1.15 95% confidence interval [95% *CI*] = 1.06–1.26).

### PheWAS of other *FTO* SNPs associated with obesity

**Figure 2:**
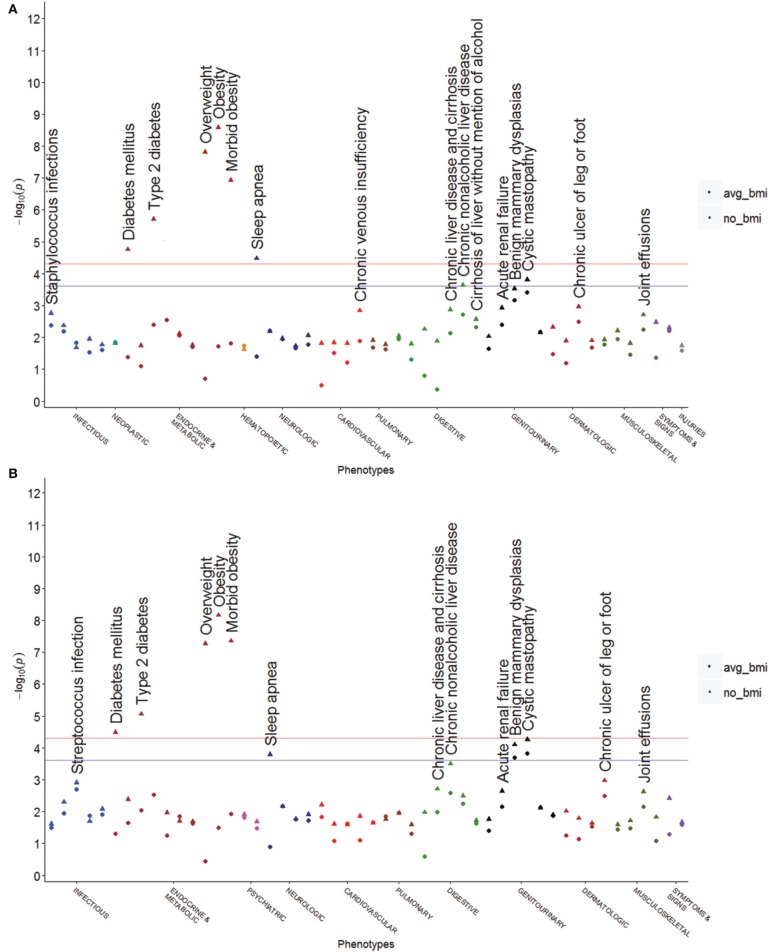
**PheWAS plots for other obesity associated SNPs in high LD with rs8050136**. These plots show unadjusted values and the average BMI adjusted values on the same axis. These SNPs are associated with BMI and have different correlations with rs8050136. These SNPs are present in both datasets and are presented as meta-analyses below. The pink horizontal line represents *p* = 4.95 × 10^−5^, which is the Bonferroni correction, and the blue horizontal line represents an FDR *q* = 0.05 (*p* = 2.48 × 10^−4^). **(A)** rs9939609 is reported widely in the literature and has a nearly identical pattern of associations to rs8050136 (*r*^2^ = 0.96). **(B)** rs9941349 also has a similar pattern to rs8050136 but cystic mastopathy is marginally more associated (*p* = 5.41 × 10^−5^, *OR* = 0.81 before BMI adjustment) than in rs8050136 (*r*^2^ = 0.88).

**Figure 3:**
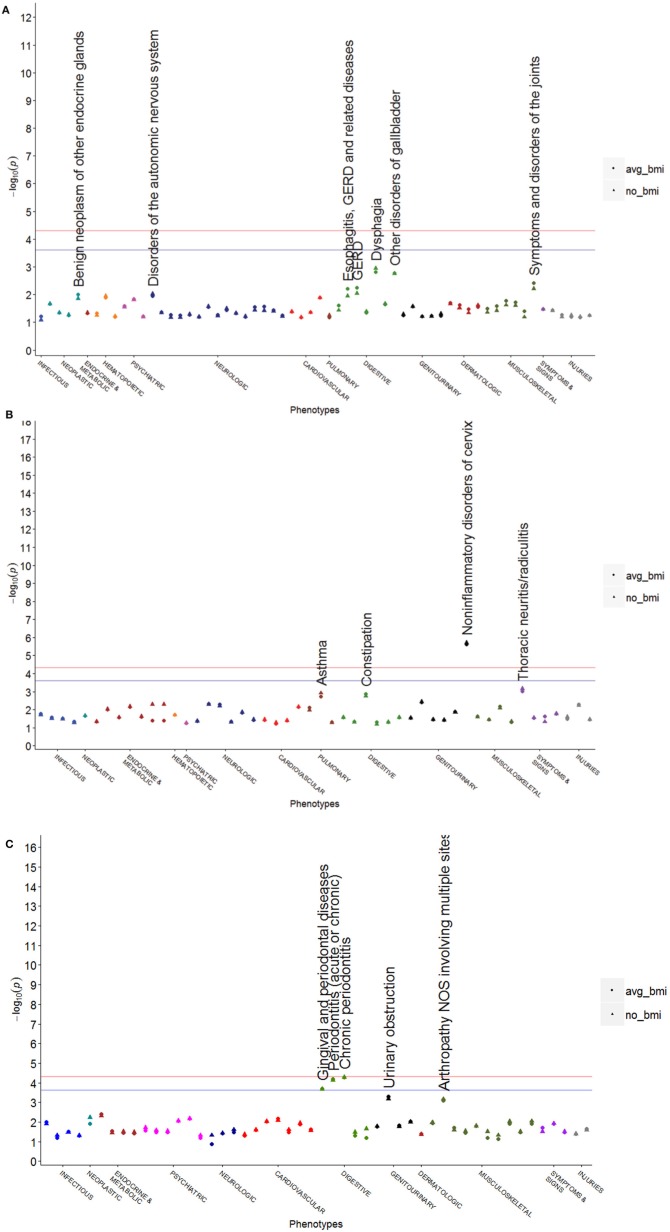
**PheWAS plots for other obesity associated SNPs in low LD with rs8050136**. These plots show values without adjustment for BMI (shown as triangles) and with adjustment for average BMI (shown as dots) plotted on the same axis. **(A)** rs6499640 is in both datasets with a lower LD with rs8050136 (*r*^2^ = 0.06) and has a different phenotype pattern than rs8050136 **(B)** rs16952520 is only present in the eMERGE population and has low LD with rs8050136 (*r*^2^ = 0.03) and while not strongly associated with obesity or diabetes does show significant association with non-inflammatory disorders of the cervix (*OR* = 6.76, *p* = 1.92 × 10^−6^), unaffected by adjustment for BMI (*OR* = 6.66, *p* = 2.36 × 10^−6^) **(C)** rs7199182 is only present in the eMERGE population and has a low LD with rs8050136 (*r*^2^ = 0.04) and is associated with chronic periodontitis before and after BMI adjustment (no adjustment: *p* = 5.40 × 10^−5^; BMI adjustment: *p* = 5.20 × 10^−5^).

The results of SNPs in high LD with rs8050136 (*r*^2^ > 0.8) showed a similar pattern of phenotypes to rs8050136 (Figures 2A,B). Rs9941349, which is in LD with rs8050136 (*r*^2^ = 0.92) trended toward association with cystic mastopathy prior to BMI adjustment (*p* = 5.4 × 10^−5^, *OR* = 0.81, 95% *CI* = 0.73–0.90). SNPs with moderate to low correlation with rs8050136 had much different patterns of associations. Some of these SNPs demonstrated associations with obesity (e.g., rs9939609, rs9941349), and some did not (e.g., rs6499640, rs7199182; see Table 2). Of these SNPs, we only had eMERGE and BioVU data for rs6499640 (Figure 3A). All other SNPs were only available in the eMERGE data. “Non-inflammatory disorders of the cervix” was associated with some *FTO* SNPs (rs16952520: *n* = 21, *p* = 1.92 × 10^−6^, *OR* = 6.76, 95% *CI* = 3.08–14.84), and was unaffected by adjustment for BMI (*OR* = 6.66, 95% *CI* = 3.03–14.64, *p* = 2.36 × 10^−6^) (Figure 3B, MAF = 0.087). One less common genetic variant rs7199182 (Figure 3C, MAF = 0.064) was associated with chronic periodontitis (202 cases, *OR* = 14.58, 95% *CI* = 3.97–53.57, *p* = 5.40 × 10^−5^), and was not changed with adjustment for BMI with the signal being slightly stronger (*OR* = 14.66, 95% *CI* = 3.99–53.84, *p* = 5.20 × 10^−5^). Neither rs16952520 nor rs7199182 were associated with obesity or T2D. Detailed results for selected SNPs are shown in Supplementary Tables 3, 4.

## Discussion

We studied the pleiotropic patterns for *FTO* variants with and without adjustment for BMI using phenome-wide associations in two large EHR cohorts. Consistent with other studies, we identified statistically significant associations with obesity, morbid obesity, and T2D among SNPs known to be associated with BMI; these associations were attenuated by adjustment for BMI. We also identified an association with OSA and trends toward association with NAFLD, fibrocystic breast disease, and infections, primarily gram-positive, with obesity-related SNPs. Some of these potential associations seem independent of BMI adjustment. Fibrocystic breast changes are a common benign breast disease and traditionally not thought related to obesity, including several epidemiological studies (Friedenreich et al., 2000; Baer et al., 2005; Li et al., 2005). Gram-positive infections could be explained in part by higher incidence of T2D in genetic variants of *FTO*. By analyzing other SNPs not significantly associated with BMI in our analysis, we also identified a few other potential associations with less common traits not associated with obesity (periodontitis, non-inflammatory diseases of the cervix); neither of these SNPs is in high LD with obesity-related SNPs. The most common ICD-9 code for “non-inflammatory disorders of the cervix” is cervical stenosis or stricture not related to congenital abnormalities or labor, which can result from surgical procedures, radiation, trauma, repeated vaginal infections, or menopause-related atrophy. These results, along with the recent association of *FTO* variants with *IRX3* regulation (Smemo et al., 2014), suggest a broader role for *FTO* beyond that of regulating fat mass.

The question of whether the association of *FTO* variants and T2D is influenced by obesity or both obesity and *FTO* has been studied previously. A UK study of 9103 individuals demonstrated the loss of association after adjustment for BMI, as the T2D-*FTO* association prior to adjustment for BMI showed an *OR* = 1.15, *p* = 9 × 10^−6^ and after adjustment showed an *OR* = 1.03, *p* = 0.44 (Frayling et al., 2007). However, other studies suggest that T2D's association with *FTO* remains after adjustment for BMI (Hertel et al., 2011; Li et al., 2012). Li et al. studied 96,551 East and South Asians and demonstrated an association with T2D (*OR* = 1.15, *p* = 5.5 × 10^−8^) that was only partially attenuated after adjustment for BMI (*OR* = 1.10, *p* = 6.6 × 10^−5^) (Li et al., 2012). Similarly, Hertel et al. observed a significant T2D-*FTO* association even after adjustment for BMI in 41,504 Scandinavians, with the *OR* prior to adjustment of 1.13, *p* = 4.5 × 10^−8^ and after adjustment, *OR* = 1.09, *p* = 1.2 × 10^−4^ (Hertel et al., 2011). Finally a meta-analysis of 24,198 individuals demonstrated *FTO* rs9939609 (in high LD with rs8050136 with *r*^2^ > 0.8) was highly significantly associated with T2D before and after adjustment for BMI (before adjustment *OR* = 1.14, 95% *CI* = 1.12–1.16, *p* = 1.00 × 10^−41^; after adjustment *OR* = 1.07, 95% *CI* = 1.05–1.09, *p* = 6.42 × 10^−41^) (Xi et al., 2014). However, among individuals of European ancestry, the association was markedly attenuated after adjustment for BMI (before adjustment *OR* = 1.14, 95% *CI* = 1.11–1.16, *p* = 1.36 × 10^−36^; after adjustment *OR* = 1.06, 95% *CI* = 1.04–1.09, *p* = 3.51 × 10^−8^). In our study, the association between *FTO* and T2D did not decrease after adjustment for BMI as markedly as phenotypes such as obesity or sleep apnea. The effect sizes of these associations with T2D in our study closely parallels these larger studies (before BMI adjustment: *OR* = 1.14, 95% *CI* = 1.08–1.21, *p* = 2.11 × 10^−6^; after adjustment: *OR* = 1.09, 95% *CI* = 1.03–1.15, *p* = 2.62 × 10^−3^). Although these results show an association of *FTO* with T2D, a mediation analysis first demonstrating the associations of *FTO* SNPs with BMI and pre-diagnostic BMI with T2D, and subsequently modeling both *FTO* SNPs and pre-diagnostic BMI on T2D would help determine the direct and indirect effects of *FTO* on T2D.

Many of our findings, while having strong signals, were not significant after Bonferroni correction. The significant associations using Bonferroni correction included obesity, T2D, and OSA prior to BMI adjustment. After adjustment for average BMI, no associations retained statistical significance, but multiple phenotypes approached significance including T2D, NAFLD, and the protective effect on fibrocystic breast disease.

There is still much debate and uncertainty about both phenotypic association and protein functionality of *FTO*. Human *FTO* protein expression studies fail to replicate *FTO*'s association with obesity observed in mouse models (Klöting et al., 2008; Wåhlén et al., 2008; Grunnet et al., 2009). Recent studies have shown that the SNPs in *FTO* that are associated with obesity regulate *IRX3* expression, which is highly expressed in the brain (Smemo et al., 2014). Studies have described the association between *FTO* and obesity, while the association between T2D and *FTO* is debated (Hubacek et al., 2008; Li et al., 2008; Xi and Mi, 2009; Liu et al., 2010; Hotta et al., 2011). More studies with larger populations are required to assess the validity of many of these associations. The results of these associations show the power of the PheWAS method to efficiently detect known and novel pleiotropic associations of genetic variants.

BMI is an inexact surrogate for adiposity. Indeed, individuals with a high BMI do not necessarily have a high body fat percentage, thus BMI may not be the optimal definition of the phenotype (Müller et al., 2010). However, BMI has been shown to be as good a surrogate for obesity and diabetes as other central obesity indicators in multiple studies and meta-analyses (Vazquez et al., 2007; Nyamdorj et al., 2008, 2009).

Prior studies have suggested several other phenotypes that may be associated with *FTO* variants, including pancreatic cancer, Alzheimer's disease, attention deficit hyperactivity disorder, and alcoholism (Keller et al., 2011; Lurie et al., 2011; Sobczyk-Kopciol et al., 2011; arcOGEN Consortium et al., 2012; Corella et al., 2012; Reitz et al., 2012; Velders et al., 2012). We did not find evidence for these associations in our data set (*p* > 0.05) (Table 4), but in these cases we may be underpowered to find an association, with case sizes of 76 (attention deficit hyperactivity disorder), 183 (pancreatic cancer), 192 (Alzheimer's disease), and 267 (alcoholism) in our population. A trend toward association between *FTO* rs8044769 and osteoarthritis was observed in a previous GWAS study (rs8044769, *r*^2^ = 0.647 with rs8050136, *p* = 4 × 10^−6^) (arcOGEN Consortium et al., 2012). Our observation of a trend toward associations with joint effusions, which may be caused by osteoarthritis, lends some support to this inflammatory association.

**Table 4:** **Meta-analysis PheWAS results of rs8050136 for previously reported phenotypes associated with genetic variants**.

**Phenotype**	**Cases**	**Not adjusted for BMI**		**Adjusted for BMI**	
		***p*^†^**	***OR* (95% *CI*)**	***p*^†^**	***OR* (95% *CI*)**
Attention deficit hyperactivity disorder	76	0.085	0.74 (0.52–1.04)	0.11	0.75 (0.53–1.06)
Pancreatic cancer	183	0.23	1.14 (0.92–1.40)	0.19	1.15 (0.93–1.42)
Alcoholism	267	0.37	1.08 (0.91–1.29)	0.32	1.09 (0.92–1.30)
Senile dementia	192	0.90	0.99 (0.80–1.22)	0.90	0.99 (0.80–1.22)
Osteoarthritis	6328	0.20	1.03 (0.98–1.08)	0.88	1.00 (0.95–1.06)

*This table includes select phenotypes that have been previously reported in the literature. The Bonferroni alpha = 0.05 equates to a p-value of 4.95 × 10^−5^, and an FDR of q = 0.05 gives a p-value of 2.48 × 10^−4^. OR, Odds ratio; CI, confidence interval*.

† *Values are not corrected for multiple testing*.

Further analysis of multiple SNPs associated with obesity in *FTO* yielded some interesting results. First, the SNPs that are in high correlation with rs8050136 (*r*^2^ > 0.8) have very similar results to rs8050136, which is what we would expect. There are also SNPs that were associated with fibrocystic breast disease prior to adjustment for BMI. rs7199182, is in low LD with rs8050136 (*r*^2^ < 0.01), showed significant associations with chronic periodontitis before and after adjustment for BMI. Further analysis of this SNP and its association with chronic periodontitis will need to be investigated to validate this finding. One important consideration of this analysis is the small overlap of genotyped SNPs between the BioVU and eMERGE population. There are multiple SNPs that are present in both datasets and are highly correlated with rs8050136, but only rs6499640, which is in weak LD with rs8050136 (*r*^2^ = 0.06), was genotyped in both datasets. We are unable to impute the BioVU. The lack of overlapping SNPs limits our sample size to evaluate more of the potentially novel findings. Limitations caution interpretation of this study. Some of the case sizes were small and will require larger populations to validate. PheWAS analyses require robust EHR systems that can query patient cohorts efficiently. We used ICD-9 codes for the determination of phenotypes, codes which can be unreliable, inaccurate, and incomplete (Kern et al., 2006; Campbell et al., 2011); however, this could tend to result in missed, rather than false, associations. In addition to the caveats of ICD-9 codes, there are limitations of multiple hypothesis testing that come with comparisons of over 1000 phenotypes. Significance corrections like Bonferroni may be too strict; some of the near-significant pleiotropic associations may, in fact, represent genuine associations. Further testing with larger populations and more carefully defined phenotypes are needed to determine whether these associations are real.

Here we demonstrate the use of the PheWAS method to illustrate pleiotropic effects of variation in the gene *FTO*. When examining this gene with known pleiotropy, we were able to reproduce previously-discovered associations and identify potential new associations, some of which appear independent of obesity.

### Conflict of interest statement

The authors declare that the research was conducted in the absence of any commercial or financial relationships that could be construed as a potential conflict of interest.
