# Supplementary material for: Phenome-wide association studies demonstrating pleiotropy of genetic variants within FTO with and without adjustment for body mass index
Source: Front Genet. 2014 Aug 5;5:250. doi: 10.3389/fgene.2014.00250 (PMC4134007; doi:10.3389/fgene.2014.00250)
Supplement: Supplementary file 1 [file DataSheet1.DOCX]

**Phenome Wide Association Studies demonstrating pleiotropy of genetic variants within *FTO* with and without adjustment for body mass index**

Contents

[Supplementary Table 1: Full results from top 250 phenotypes of PheWAS analysis of rs8050136 for each population 2](#_Toc390686231)

[Supplementary Table 2: Top 100 phenotypes from the meta-analysis of SNP rs6499640, in low association with rs8050136. 35](#_Toc390686232)

[Supplementary Table 3: Results from the top 100 phenotypes from the eMERGE PheWAS of rs7199182, in low association with rs8050136. 39](#_Toc390686233)

## Supplementary Table 1: Full results from top 250 phenotypes of PheWAS analysis of rs8050136 for each population. The table is a detailed description of the PheWAS analysis of rs8050136 for each population (eMERGE n=10,487 and BioVU n=13,711). The columns state the number of cases in each population, the adjustment (either by average BMI (avg_bmi) or no adjustment (no_bmi), the raw p-value, the Odds Ratio (OR), and the 95% Confidence Interval (95% CI). The Bonferroni alpha=0.05 equates to a p-value of 4.95x10^-5^, and an FDR of q=0.05 gives a p-value of 2.48x10^-4^. The ICD-9 codes that are associated with each phenotype can be found online at http://phewas.mc.vanderbilt.edu/. The code groupings used here are identical to those in (Denny et al., 2013).

| **eMERGE** | | | | | | | | | |
| --- | --- | --- | --- | --- | --- | --- | --- | --- | --- |
|  |  |  |  |  |  |  |  |  |  |
| **PheWAS description** | **Cases** | **adjustment** | **p†** | **OR** | **95% CI** | | | | |
| **End stage renal disease** | **57** | **avg_bmi** | **3.47E-04** | **1.98** | **(** | **1.36** | **-** | **2.88** | **)** |
| **Chronic ulcer of leg or foot** | **440** | **avg_bmi** | **2.13E-03** | **1.24** | **(** | **1.08** | **-** | **1.43** | **)** |
| **Chronic ulcer of skin** | **611** | **avg_bmi** | **4.14E-03** | **1.19** | **(** | **1.06** | **-** | **1.34** | **)** |
| **Emphysema** | **128** | **avg_bmi** | **6.03E-03** | **0.69** | **(** | **0.53** | **-** | **0.90** | **)** |
| **Abnormal results of function study of liver** | **179** | **avg_bmi** | **9.17E-03** | **1.32** | **(** | **1.07** | **-** | **1.63** | **)** |
| **Cystic mastopathy** | **518** | **avg_bmi** | **0.010** | **0.82** | **(** | **0.70** | **-** | **0.95** | **)** |
| **Disturbances of sensation of smell and taste** | **24** | **avg_bmi** | **0.010** | **2.14** | **(** | **1.20** | **-** | **3.82** | **)** |
| **Abnormal cytological, histological, immunological and DNA test findings** | **169** | **avg_bmi** | **0.011** | **1.34** | **(** | **1.07** | **-** | **1.69** | **)** |
| **Fracture of lower limb** | **375** | **avg_bmi** | **0.012** | **1.22** | **(** | **1.04** | **-** | **1.42** | **)** |
| **Corns and callosities** | **364** | **avg_bmi** | **0.015** | **1.21** | **(** | **1.04** | **-** | **1.42** | **)** |
| **Arterial embolism and thrombosis of lower extremity artery** | **108** | **avg_bmi** | **0.017** | **1.40** | **(** | **1.06** | **-** | **1.83** | **)** |
| **Retinal edema and hypertensive retinopathy** | **107** | **avg_bmi** | **0.018** | **1.39** | **(** | **1.06** | **-** | **1.83** | **)** |
| **Viral warts & HPV** | **425** | **avg_bmi** | **0.019** | **1.19** | **(** | **1.03** | **-** | **1.37** | **)** |
| **Immunity deficiency** | **26** | **avg_bmi** | **0.019** | **1.92** | **(** | **1.11** | **-** | **3.31** | **)** |
| **Influenza** | **42** | **avg_bmi** | **0.019** | **0.56** | **(** | **0.34** | **-** | **0.91** | **)** |
| **Abnormal findings on radiological examination intrathoracic organs** | **119** | **avg_bmi** | **0.020** | **1.38** | **(** | **1.05** | **-** | **1.80** | **)** |
| **Scar conditions and fibrosis of skin** | **106** | **avg_bmi** | **0.021** | **1.38** | **(** | **1.05** | **-** | **1.81** | **)** |
| **Anemia of chronic disease** | **202** | **avg_bmi** | **0.023** | **1.26** | **(** | **1.03** | **-** | **1.54** | **)** |
| **Other specified diseases of sebaceous glands** | **160** | **avg_bmi** | **0.023** | **0.76** | **(** | **0.60** | **-** | **0.96** | **)** |
| **Other hypertrophic and atrophic conditions of skin** | **1045** | **avg_bmi** | **0.025** | **1.12** | **(** | **1.01** | **-** | **1.24** | **)** |
| **Chronic tonsillitis and adenoiditis** | **27** | **avg_bmi** | **0.027** | **1.84** | **(** | **1.07** | **-** | **3.17** | **)** |
| **Other abnormality of urination** | **219** | **avg_bmi** | **0.027** | **1.24** | **(** | **1.02** | **-** | **1.50** | **)** |
| **Behcet's syndrome** | **48** | **avg_bmi** | **0.029** | **0.62** | **(** | **0.40** | **-** | **0.95** | **)** |
| **Other disorders of peritoneum** | **53** | **avg_bmi** | **0.030** | **1.53** | **(** | **1.04** | **-** | **2.24** | **)** |
| **Acute renal failure** | **305** | **avg_bmi** | **0.031** | **1.20** | **(** | **1.02** | **-** | **1.42** | **)** |
| **Obstruction of bile duct** | **32** | **avg_bmi** | **0.031** | **0.54** | **(** | **0.31** | **-** | **0.95** | **)** |
| **Spinal stenosis of lumbar region** | **456** | **avg_bmi** | **0.032** | **1.16** | **(** | **1.01** | **-** | **1.34** | **)** |
| **Other disorders of the nervous system** | **38** | **avg_bmi** | **0.033** | **1.63** | **(** | **1.04** | **-** | **2.55** | **)** |
| **Anemia in chronic kidney disease** | **75** | **avg_bmi** | **0.033** | **1.41** | **(** | **1.03** | **-** | **1.95** | **)** |
| **Benign mammary dysplasias** | **654** | **avg_bmi** | **0.034** | **0.86** | **(** | **0.75** | **-** | **0.99** | **)** |
| **Diverticulitis** | **202** | **avg_bmi** | **0.035** | **1.25** | **(** | **1.02** | **-** | **1.53** | **)** |
| **Eating disorder** | **52** | **avg_bmi** | **0.035** | **1.53** | **(** | **1.03** | **-** | **2.26** | **)** |
| **Peripheral retinal degenerations** | **105** | **avg_bmi** | **0.037** | **1.34** | **(** | **1.02** | **-** | **1.77** | **)** |
| **Muscular wasting and disuse atrophy** | **33** | **avg_bmi** | **0.037** | **0.56** | **(** | **0.33** | **-** | **0.97** | **)** |
| **Intestinal malabsorption NOS** | **22** | **avg_bmi** | **0.038** | **1.87** | **(** | **1.04** | **-** | **3.38** | **)** |
| **Second degree AV block** | **32** | **avg_bmi** | **0.038** | **1.70** | **(** | **1.03** | **-** | **2.79** | **)** |
| **Irritable Bowel Syndrome** | **365** | **avg_bmi** | **0.038** | **1.19** | **(** | **1.01** | **-** | **1.39** | **)** |
| **Spinal stenosis** | **475** | **avg_bmi** | **0.039** | **1.15** | **(** | **1.01** | **-** | **1.32** | **)** |
| **Arthralgia/ankylosis of temporomandibular joint** | **29** | **avg_bmi** | **0.039** | **1.73** | **(** | **1.03** | **-** | **2.93** | **)** |
| **Chronic ulcer of unspecified site** | **123** | **avg_bmi** | **0.040** | **1.30** | **(** | **1.01** | **-** | **1.68** | **)** |
| **Calculus of bile duct** | **86** | **avg_bmi** | **0.041** | **0.72** | **(** | **0.52** | **-** | **0.99** | **)** |
| **Macular puckering of retina** | **227** | **avg_bmi** | **0.041** | **1.22** | **(** | **1.01** | **-** | **1.48** | **)** |
| **Lipoma of skin & subcutaneous tissue** | **114** | **avg_bmi** | **0.042** | **0.75** | **(** | **0.56** | **-** | **0.99** | **)** |
| **Cholelithiasis with other cholecystitis** | **90** | **avg_bmi** | **0.042** | **1.36** | **(** | **1.01** | **-** | **1.83** | **)** |
| **Other specified cardiac dysrhythmias** | **788** | **avg_bmi** | **0.042** | **1.13** | **(** | **1.00** | **-** | **1.28** | **)** |
| **Chronic nonalcoholic liver disease** | **221** | **avg_bmi** | **0.043** | **1.22** | **(** | **1.01** | **-** | **1.49** | **)** |
| **Other disorders of testis** | **75** | **avg_bmi** | **0.043** | **0.69** | **(** | **0.49** | **-** | **0.99** | **)** |
| **Thoracic neuritis/radiculitis** | **634** | **avg_bmi** | **0.045** | **1.13** | **(** | **1.00** | **-** | **1.28** | **)** |
| **Testicular dysfunction** | **91** | **avg_bmi** | **0.045** | **0.73** | **(** | **0.53** | **-** | **0.99** | **)** |
| **Esophageal cancer** | **33** | **avg_bmi** | **0.045** | **1.66** | **(** | **1.01** | **-** | **2.72** | **)** |
| **Chronic periodontitis** | **202** | **avg_bmi** | **0.046** | **0.81** | **(** | **0.65** | **-** | **1.00** | **)** |
| **Lipoma** | **247** | **avg_bmi** | **0.049** | **0.83** | **(** | **0.69** | **-** | **1.00** | **)** |
| **Obstructive chronic bronchitis** | **188** | **avg_bmi** | **0.050** | **0.80** | **(** | **0.65** | **-** | **1.00** | **)** |
| **Hemoptysis** | **76** | **avg_bmi** | **0.052** | **0.71** | **(** | **0.50** | **-** | **1.00** | **)** |
| **Other open wound of head and face** | **164** | **avg_bmi** | **0.054** | **1.25** | **(** | **1.00** | **-** | **1.56** | **)** |
| **Ventral hernia** | **141** | **avg_bmi** | **0.054** | **1.26** | **(** | **1.00** | **-** | **1.60** | **)** |
| **Allergic conjunctivitis** | **119** | **avg_bmi** | **0.055** | **1.31** | **(** | **0.99** | **-** | **1.71** | **)** |
| **Decubitus ulcer** | **112** | **avg_bmi** | **0.056** | **1.30** | **(** | **0.99** | **-** | **1.69** | **)** |
| **Cirrhosis of liver without mention of alcohol** | **80** | **avg_bmi** | **0.056** | **1.36** | **(** | **0.99** | **-** | **1.87** | **)** |
| **Disorders of synovium, tendon, and bursa** | **1114** | **avg_bmi** | **0.056** | **1.10** | **(** | **1.00** | **-** | **1.22** | **)** |
| **Chronic liver disease and cirrhosis** | **258** | **avg_bmi** | **0.056** | **1.19** | **(** | **1.00** | **-** | **1.43** | **)** |
| **Temporomandibular joint disorders** | **117** | **avg_bmi** | **0.058** | **1.29** | **(** | **0.99** | **-** | **1.68** | **)** |
| **Appendiceal conditions** | **110** | **avg_bmi** | **0.059** | **0.76** | **(** | **0.57** | **-** | **1.01** | **)** |
| **Cancer, suspected or other** | **173** | **avg_bmi** | **0.060** | **1.23** | **(** | **0.99** | **-** | **1.52** | **)** |
| **Chronic renal failure** | **894** | **avg_bmi** | **0.061** | **1.10** | **(** | **1.00** | **-** | **1.22** | **)** |
| **Myeloproliferative disease** | **86** | **avg_bmi** | **0.061** | **0.74** | **(** | **0.53** | **-** | **1.01** | **)** |
| **Urinary obstruction** | **40** | **avg_bmi** | **0.065** | **0.64** | **(** | **0.39** | **-** | **1.03** | **)** |
| **Other aneurysm** | **865** | **avg_bmi** | **0.067** | **0.90** | **(** | **0.80** | **-** | **1.01** | **)** |
| **Hemangioma and lymphangioma, any site** | **116** | **avg_bmi** | **0.068** | **1.28** | **(** | **0.98** | **-** | **1.66** | **)** |
| **Polycythemia vera** | **23** | **avg_bmi** | **0.068** | **0.54** | **(** | **0.28** | **-** | **1.05** | **)** |
| **Other cells and casts in urine** | **75** | **avg_bmi** | **0.069** | **1.40** | **(** | **0.97** | **-** | **2.01** | **)** |
| **Nonrheumatic aortic valve disorders** | **472** | **avg_bmi** | **0.069** | **0.88** | **(** | **0.76** | **-** | **1.01** | **)** |
| **Other congenital anomalies of skin** | **41** | **avg_bmi** | **0.071** | **1.49** | **(** | **0.97** | **-** | **2.29** | **)** |
| **Fracture of humerus** | **144** | **avg_bmi** | **0.072** | **1.24** | **(** | **0.98** | **-** | **1.58** | **)** |
| **Displacement of intervertebral disc** | **291** | **avg_bmi** | **0.072** | **1.17** | **(** | **0.99** | **-** | **1.38** | **)** |
| **Coronary atherosclerosis** | **1468** | **avg_bmi** | **0.073** | **1.09** | **(** | **0.99** | **-** | **1.19** | **)** |
| **Testicular hypofunction** | **83** | **avg_bmi** | **0.074** | **0.74** | **(** | **0.53** | **-** | **1.03** | **)** |
| **Vascular insufficiency of intestine** | **79** | **avg_bmi** | **0.074** | **1.33** | **(** | **0.97** | **-** | **1.82** | **)** |
| **Noninflammatory disorders of vagina** | **85** | **avg_bmi** | **0.074** | **1.32** | **(** | **0.97** | **-** | **1.80** | **)** |
| **Periodontitis (acute or chronic)** | **227** | **avg_bmi** | **0.075** | **0.83** | **(** | **0.68** | **-** | **1.02** | **)** |
| **Gastroparesis** | **25** | **avg_bmi** | **0.075** | **1.66** | **(** | **0.95** | **-** | **2.90** | **)** |
| **Conjunctivitis, infectious** | **235** | **avg_bmi** | **0.075** | **1.20** | **(** | **0.98** | **-** | **1.45** | **)** |
| **Diseases of white blood cells** | **354** | **avg_bmi** | **0.075** | **1.15** | **(** | **0.99** | **-** | **1.34** | **)** |
| **Urethral stricture (not specified as infectious)** | **53** | **avg_bmi** | **0.075** | **0.69** | **(** | **0.46** | **-** | **1.04** | **)** |
| **Dermatophytosis of nail** | **564** | **avg_bmi** | **0.076** | **0.88** | **(** | **0.77** | **-** | **1.01** | **)** |
| **Chronic venous insufficiency** | **271** | **avg_bmi** | **0.078** | **1.18** | **(** | **0.98** | **-** | **1.43** | **)** |
| **Fracture of neck of femur** | **204** | **avg_bmi** | **0.078** | **1.21** | **(** | **0.98** | **-** | **1.48** | **)** |
| **Adverse drug events and drug allergies** | **237** | **avg_bmi** | **0.080** | **1.18** | **(** | **0.98** | **-** | **1.42** | **)** |
| **Retinal drusen** | **199** | **avg_bmi** | **0.080** | **1.21** | **(** | **0.98** | **-** | **1.49** | **)** |
| **Acute periodontitis** | **27** | **avg_bmi** | **0.082** | **0.59** | **(** | **0.32** | **-** | **1.07** | **)** |
| **Conjunctivitis, noninfectious** | **148** | **avg_bmi** | **0.085** | **1.24** | **(** | **0.97** | **-** | **1.59** | **)** |
| **Cellulitis and abscess of foot/toes** | **117** | **avg_bmi** | **0.088** | **1.26** | **(** | **0.97** | **-** | **1.63** | **)** |
| **Disturbances of amino-acid transport** | **29** | **avg_bmi** | **0.089** | **1.57** | **(** | **0.93** | **-** | **2.66** | **)** |
| **Disturbances of sulphur-bearing amino-acid metabolism** | **29** | **avg_bmi** | **0.089** | **1.57** | **(** | **0.93** | **-** | **2.66** | **)** |
| **Other disorders of biliary tract** | **46** | **avg_bmi** | **0.089** | **0.68** | **(** | **0.44** | **-** | **1.06** | **)** |
| **Hypotension NOS** | **175** | **avg_bmi** | **0.091** | **1.21** | **(** | **0.97** | **-** | **1.50** | **)** |
| **Elevated white blood cell count** | **101** | **avg_bmi** | **0.091** | **1.28** | **(** | **0.96** | **-** | **1.69** | **)** |
| **Hydrocele** | **47** | **avg_bmi** | **0.091** | **0.68** | **(** | **0.43** | **-** | **1.06** | **)** |
| **Angina pectoris** | **627** | **avg_bmi** | **0.092** | **1.11** | **(** | **0.98** | **-** | **1.26** | **)** |
| **Breast conditions, congenital or relating to hormones** | **86** | **avg_bmi** | **0.092** | **1.30** | **(** | **0.96** | **-** | **1.76** | **)** |
| **Keloid scar** | **26** | **avg_bmi** | **0.092** | **1.61** | **(** | **0.92** | **-** | **2.80** | **)** |
| **Protein plasma/amino-acid transport and metabolism disorder** | **150** | **avg_bmi** | **0.093** | **1.22** | **(** | **0.97** | **-** | **1.53** | **)** |
| **Secondary malignancy of lymph nodes** | **158** | **avg_bmi** | **0.094** | **1.21** | **(** | **0.97** | **-** | **1.52** | **)** |
| **Thyrotoxicosis** | **128** | **avg_bmi** | **0.095** | **1.24** | **(** | **0.96** | **-** | **1.59** | **)** |
| **Elevated levels of transaminase or lactic acid dehydrogenase** | **180** | **avg_bmi** | **0.096** | **1.21** | **(** | **0.97** | **-** | **1.51** | **)** |
| **Duodenitis** | **86** | **avg_bmi** | **0.097** | **1.30** | **(** | **0.95** | **-** | **1.78** | **)** |
| **Insomnia** | **470** | **avg_bmi** | **0.098** | **1.12** | **(** | **0.98** | **-** | **1.28** | **)** |
| **Fracture of tibia and fibula** | **107** | **avg_bmi** | **0.099** | **1.26** | **(** | **0.96** | **-** | **1.66** | **)** |
| **Cardiac complications, not elsewhere classified** | **52** | **avg_bmi** | **0.100** | **0.70** | **(** | **0.46** | **-** | **1.07** | **)** |
| **Lichen** | **48** | **avg_bmi** | **0.100** | **1.40** | **(** | **0.94** | **-** | **2.09** | **)** |
| **Cancer of the upper aerodigestive tract** | **49** | **avg_bmi** | **0.101** | **0.70** | **(** | **0.46** | **-** | **1.07** | **)** |
| **Disorders of iris and ciliary body** | **116** | **avg_bmi** | **0.102** | **0.79** | **(** | **0.60** | **-** | **1.05** | **)** |
| **Immune disorders** | **117** | **avg_bmi** | **0.103** | **1.24** | **(** | **0.96** | **-** | **1.61** | **)** |
| **Atrophic gastritis** | **56** | **avg_bmi** | **0.103** | **1.37** | **(** | **0.94** | **-** | **2.00** | **)** |
| **Paralytic ileus** | **77** | **avg_bmi** | **0.104** | **1.31** | **(** | **0.95** | **-** | **1.80** | **)** |
| **Cardiac congenital anomalies** | **98** | **avg_bmi** | **0.104** | **1.26** | **(** | **0.95** | **-** | **1.68** | **)** |
| **Congenital anomalies of great vessels** | **21** | **avg_bmi** | **0.106** | **1.65** | **(** | **0.90** | **-** | **3.02** | **)** |
| **Spondylosis with myelopathy** | **73** | **avg_bmi** | **0.106** | **1.31** | **(** | **0.94** | **-** | **1.82** | **)** |
| **Open wound of hand except finger(s)** | **87** | **avg_bmi** | **0.106** | **1.29** | **(** | **0.95** | **-** | **1.76** | **)** |
| **Open wound of foot except toe(s) alone** | **32** | **avg_bmi** | **0.106** | **0.64** | **(** | **0.38** | **-** | **1.10** | **)** |
| **Hypoventilation** | **29** | **avg_bmi** | **0.107** | **1.55** | **(** | **0.91** | **-** | **2.64** | **)** |
| **Prolapse of vaginal vault after hysterectomy** | **53** | **avg_bmi** | **0.108** | **0.71** | **(** | **0.47** | **-** | **1.08** | **)** |
| **Hypotension** | **472** | **avg_bmi** | **0.108** | **1.12** | **(** | **0.98** | **-** | **1.28** | **)** |
| **Fever of unknown origin** | **568** | **avg_bmi** | **0.109** | **1.11** | **(** | **0.98** | **-** | **1.25** | **)** |
| **Respiratory abnormalities** | **65** | **avg_bmi** | **0.109** | **1.34** | **(** | **0.94** | **-** | **1.90** | **)** |
| **Other alveolar and parietoalveolar pneumonopathy** | **34** | **avg_bmi** | **0.109** | **1.48** | **(** | **0.92** | **-** | **2.37** | **)** |
| **Osteoarthrosis NOS** | **2286** | **avg_bmi** | **0.110** | **1.07** | **(** | **0.98** | **-** | **1.17** | **)** |
| **Somatoform disorder** | **64** | **avg_bmi** | **0.110** | **1.34** | **(** | **0.94** | **-** | **1.92** | **)** |
| **Nontoxic multinodular goiter** | **94** | **avg_bmi** | **0.110** | **0.78** | **(** | **0.58** | **-** | **1.06** | **)** |
| **Optic neuritis/neuropathy** | **81** | **avg_bmi** | **0.111** | **0.76** | **(** | **0.55** | **-** | **1.06** | **)** |
| **Alcoholism** | **78** | **avg_bmi** | **0.111** | **1.30** | **(** | **0.94** | **-** | **1.80** | **)** |
| **Atrial fibrillation & flutter** | **1003** | **avg_bmi** | **0.112** | **1.09** | **(** | **0.98** | **-** | **1.22** | **)** |
| **Cerebral aneurysm** | **32** | **avg_bmi** | **0.112** | **0.65** | **(** | **0.38** | **-** | **1.11** | **)** |
| **Diseases of hard tissues of teeth** | **269** | **avg_bmi** | **0.114** | **0.87** | **(** | **0.72** | **-** | **1.04** | **)** |
| **Dermatophytosis of the body** | **84** | **avg_bmi** | **0.115** | **1.29** | **(** | **0.94** | **-** | **1.76** | **)** |
| **Herpes zoster with nervous system complications** | **40** | **avg_bmi** | **0.116** | **1.42** | **(** | **0.92** | **-** | **2.21** | **)** |
| **Crystal arthropathies** | **75** | **avg_bmi** | **0.116** | **1.30** | **(** | **0.94** | **-** | **1.80** | **)** |
| **Inflammatory spondylopathies** | **37** | **avg_bmi** | **0.116** | **1.45** | **(** | **0.91** | **-** | **2.30** | **)** |
| **Type 1 diabetic neuropathy** | **75** | **avg_bmi** | **0.117** | **0.76** | **(** | **0.54** | **-** | **1.07** | **)** |
| **Pneumonia** | **1089** | **avg_bmi** | **0.117** | **0.92** | **(** | **0.83** | **-** | **1.02** | **)** |
| **Brain cancer** | **30** | **avg_bmi** | **0.121** | **1.49** | **(** | **0.90** | **-** | **2.46** | **)** |
| **Acute and chronic tonsillitis** | **56** | **avg_bmi** | **0.121** | **1.35** | **(** | **0.92** | **-** | **1.99** | **)** |
| **Cancer of brain and nervous system** | **30** | **avg_bmi** | **0.121** | **1.49** | **(** | **0.90** | **-** | **2.45** | **)** |
| **Atherosclerosis of native arteries of the extremities with ulceration or gangrene** | **184** | **avg_bmi** | **0.121** | **1.19** | **(** | **0.96** | **-** | **1.47** | **)** |
| **Abnormal glucose** | **1109** | **avg_bmi** | **0.122** | **0.92** | **(** | **0.82** | **-** | **1.02** | **)** |
| **Complication of internal orthopedic device** | **191** | **avg_bmi** | **0.122** | **0.85** | **(** | **0.69** | **-** | **1.05** | **)** |
| **Sciatica** | **269** | **avg_bmi** | **0.123** | **1.16** | **(** | **0.96** | **-** | **1.39** | **)** |
| **Obesity** | **317** | **avg_bmi** | **0.125** | **1.18** | **(** | **0.95** | **-** | **1.47** | **)** |
| **Morbid obesity** | **317** | **avg_bmi** | **0.125** | **1.18** | **(** | **0.95** | **-** | **1.47** | **)** |
| **Chronic prostatitis** | **57** | **avg_bmi** | **0.125** | **1.35** | **(** | **0.92** | **-** | **1.98** | **)** |
| **Sepsis** | **79** | **avg_bmi** | **0.125** | **1.28** | **(** | **0.93** | **-** | **1.75** | **)** |
| **Other anemias** | **1831** | **avg_bmi** | **0.125** | **1.07** | **(** | **0.98** | **-** | **1.16** | **)** |
| **Psychogenic disorder** | **32** | **avg_bmi** | **0.126** | **0.65** | **(** | **0.37** | **-** | **1.13** | **)** |
| **Other benign neoplasm of connective and other soft tissue** | **52** | **avg_bmi** | **0.127** | **1.36** | **(** | **0.92** | **-** | **2.01** | **)** |
| **Hypertrophy of breast (Gynecomastia)** | **70** | **avg_bmi** | **0.128** | **1.30** | **(** | **0.93** | **-** | **1.81** | **)** |
| **Cervical intraepithelial neoplasia (Cervical dysplasia)** | **107** | **avg_bmi** | **0.130** | **1.24** | **(** | **0.94** | **-** | **1.64** | **)** |
| **Deviated nasal septum** | **214** | **avg_bmi** | **0.130** | **1.17** | **(** | **0.95** | **-** | **1.45** | **)** |
| **Superficial cellulitis and abscess** | **1298** | **avg_bmi** | **0.131** | **1.07** | **(** | **0.98** | **-** | **1.17** | **)** |
| **Infection of the eye** | **623** | **avg_bmi** | **0.131** | **1.11** | **(** | **0.97** | **-** | **1.26** | **)** |
| **Esophagitis, GERD and related diseases** | **2286** | **avg_bmi** | **0.132** | **0.94** | **(** | **0.87** | **-** | **1.02** | **)** |
| **Purpura and other hemorrhagic conditions** | **365** | **avg_bmi** | **0.133** | **0.89** | **(** | **0.76** | **-** | **1.04** | **)** |
| **Cervical cancer and dysplasia** | **119** | **avg_bmi** | **0.133** | **1.22** | **(** | **0.94** | **-** | **1.59** | **)** |
| **Excessive or frequent menstruation** | **189** | **avg_bmi** | **0.134** | **0.83** | **(** | **0.64** | **-** | **1.06** | **)** |
| **Cramp of limb** | **102** | **avg_bmi** | **0.134** | **1.24** | **(** | **0.94** | **-** | **1.63** | **)** |
| **Other acquired musculoskeletal deformity** | **167** | **avg_bmi** | **0.134** | **1.18** | **(** | **0.95** | **-** | **1.47** | **)** |
| **Conduct disorders** | **22** | **avg_bmi** | **0.134** | **1.58** | **(** | **0.87** | **-** | **2.88** | **)** |
| **Sleep apnea** | **1393** | **avg_bmi** | **0.135** | **1.07** | **(** | **0.98** | **-** | **1.18** | **)** |
| **Failure to thrive** | **47** | **avg_bmi** | **0.136** | **0.72** | **(** | **0.46** | **-** | **1.11** | **)** |
| **Bronchopneumonia and lung abscess** | **26** | **avg_bmi** | **0.138** | **0.64** | **(** | **0.35** | **-** | **1.16** | **)** |
| **Peptic ulcers** | **110** | **avg_bmi** | **0.138** | **1.23** | **(** | **0.94** | **-** | **1.62** | **)** |
| **Premature menopause and other ovarian failure** | **24** | **avg_bmi** | **0.139** | **1.54** | **(** | **0.87** | **-** | **2.73** | **)** |
| **Neurological disorders due to brain damage** | **560** | **avg_bmi** | **0.139** | **0.90** | **(** | **0.79** | **-** | **1.03** | **)** |
| **Hyposmolality and/or hyponatremia** | **338** | **avg_bmi** | **0.139** | **1.13** | **(** | **0.96** | **-** | **1.33** | **)** |
| **Cardiac pacemaker/device in situ** | **26** | **avg_bmi** | **0.141** | **1.51** | **(** | **0.87** | **-** | **2.62** | **)** |
| **Other disorders of middle ear and mastoid** | **32** | **avg_bmi** | **0.142** | **1.45** | **(** | **0.88** | **-** | **2.38** | **)** |
| **Type 2 diabetes** | **630** | **avg_bmi** | **0.142** | **1.10** | **(** | **0.97** | **-** | **1.25** | **)** |
| **Nonallopathic lesions NEC** | **188** | **avg_bmi** | **0.143** | **0.85** | **(** | **0.69** | **-** | **1.06** | **)** |
| **Heartburn** | **68** | **avg_bmi** | **0.143** | **1.30** | **(** | **0.91** | **-** | **1.86** | **)** |
| **Arterial embolism and thrombosis** | **254** | **avg_bmi** | **0.144** | **1.15** | **(** | **0.95** | **-** | **1.38** | **)** |
| **Abnormal sputum** | **89** | **avg_bmi** | **0.145** | **0.79** | **(** | **0.58** | **-** | **1.08** | **)** |
| **Unspecified osteomyelitis** | **104** | **avg_bmi** | **0.145** | **1.23** | **(** | **0.93** | **-** | **1.61** | **)** |
| **Peripheral or central vertigo** | **225** | **avg_bmi** | **0.146** | **1.15** | **(** | **0.95** | **-** | **1.40** | **)** |
| **Atherosclerosis of the extremities** | **1136** | **avg_bmi** | **0.146** | **1.08** | **(** | **0.97** | **-** | **1.20** | **)** |
| **Atrial fibrillation** | **896** | **avg_bmi** | **0.148** | **1.09** | **(** | **0.97** | **-** | **1.22** | **)** |
| **Atrial flutter** | **169** | **avg_bmi** | **0.149** | **1.18** | **(** | **0.94** | **-** | **1.47** | **)** |
| **Abnormal Papanicolaou smear of cervix and cervical HPV** | **65** | **avg_bmi** | **0.149** | **1.29** | **(** | **0.91** | **-** | **1.83** | **)** |
| **Cellulitis and abscess of arm** | **147** | **avg_bmi** | **0.150** | **1.19** | **(** | **0.94** | **-** | **1.51** | **)** |
| **Disorders of adrenal glands** | **88** | **avg_bmi** | **0.152** | **1.24** | **(** | **0.92** | **-** | **1.68** | **)** |
| **Gross hematuria** | **38** | **avg_bmi** | **0.156** | **1.40** | **(** | **0.88** | **-** | **2.22** | **)** |
| **Endometrial hyperplasia** | **42** | **avg_bmi** | **0.159** | **1.36** | **(** | **0.89** | **-** | **2.10** | **)** |
| **Intestinal obstruction without mention of hernia** | **314** | **avg_bmi** | **0.160** | **1.13** | **(** | **0.95** | **-** | **1.33** | **)** |
| **Chronic sinusitis** | **798** | **avg_bmi** | **0.160** | **1.09** | **(** | **0.97** | **-** | **1.23** | **)** |
| **H. pylori** | **36** | **avg_bmi** | **0.160** | **1.39** | **(** | **0.88** | **-** | **2.21** | **)** |
| **Nasal polyps** | **76** | **avg_bmi** | **0.161** | **1.26** | **(** | **0.91** | **-** | **1.75** | **)** |
| **Renal failure** | **1225** | **avg_bmi** | **0.163** | **1.07** | **(** | **0.97** | **-** | **1.17** | **)** |
| **Paralysis/spasm of vocal cords or larynx** | **28** | **avg_bmi** | **0.164** | **1.46** | **(** | **0.86** | **-** | **2.47** | **)** |
| **Dysthymic disorder** | **315** | **avg_bmi** | **0.165** | **1.13** | **(** | **0.95** | **-** | **1.34** | **)** |
| **Alcoholic liver damage** | **28** | **avg_bmi** | **0.165** | **1.46** | **(** | **0.86** | **-** | **2.48** | **)** |
| **Antisocial/borderline personality disorder** | **24** | **avg_bmi** | **0.167** | **1.51** | **(** | **0.84** | **-** | **2.69** | **)** |
| **Chondrocalcinosis** | **73** | **avg_bmi** | **0.167** | **1.26** | **(** | **0.91** | **-** | **1.76** | **)** |
| **Crohn's disease** | **72** | **avg_bmi** | **0.168** | **0.78** | **(** | **0.55** | **-** | **1.11** | **)** |
| **Chronic bronchitis** | **250** | **avg_bmi** | **0.170** | **0.88** | **(** | **0.73** | **-** | **1.06** | **)** |
| **Hypertensive chronic kidney disease** | **375** | **avg_bmi** | **0.171** | **1.12** | **(** | **0.95** | **-** | **1.32** | **)** |
| **Other disorders of bone and cartilage** | **1153** | **avg_bmi** | **0.172** | **1.07** | **(** | **0.97** | **-** | **1.19** | **)** |
| **Cellulitis and abscess of leg** | **304** | **avg_bmi** | **0.172** | **1.13** | **(** | **0.95** | **-** | **1.34** | **)** |
| **Other disorders of urethra and urinary tract** | **1575** | **avg_bmi** | **0.174** | **1.06** | **(** | **0.97** | **-** | **1.17** | **)** |
| **Unspecified polyarthropathy or polyarthritis** | **30** | **avg_bmi** | **0.178** | **0.69** | **(** | **0.40** | **-** | **1.19** | **)** |
| **Varicose veins of lower extremity, symptomtic** | **183** | **avg_bmi** | **0.179** | **1.16** | **(** | **0.93** | **-** | **1.44** | **)** |
| **Paralytic strabismus** | **42** | **avg_bmi** | **0.181** | **1.34** | **(** | **0.87** | **-** | **2.07** | **)** |
| **Atherosclerosis** | **1466** | **avg_bmi** | **0.182** | **1.07** | **(** | **0.97** | **-** | **1.17** | **)** |
| **Eosinophilia** | **23** | **avg_bmi** | **0.182** | **1.48** | **(** | **0.83** | **-** | **2.65** | **)** |
| **Lymphadenitis** | **301** | **avg_bmi** | **0.183** | **1.12** | **(** | **0.95** | **-** | **1.33** | **)** |
| **Methicillin sensitive Staphylococcus aureus** | **41** | **avg_bmi** | **0.183** | **1.34** | **(** | **0.87** | **-** | **2.07** | **)** |
| **Other cardiac conduction disorders** | **102** | **avg_bmi** | **0.184** | **1.21** | **(** | **0.91** | **-** | **1.60** | **)** |
| **Cancer of kidney and urinary organs** | **264** | **avg_bmi** | **0.184** | **1.13** | **(** | **0.94** | **-** | **1.34** | **)** |
| **Genu valgum or varum (acquired)** | **44** | **avg_bmi** | **0.185** | **1.34** | **(** | **0.87** | **-** | **2.06** | **)** |
| **Gram negative septicemia** | **35** | **avg_bmi** | **0.186** | **0.71** | **(** | **0.43** | **-** | **1.18** | **)** |
| **Sepsis and SIRS** | **94** | **avg_bmi** | **0.187** | **1.22** | **(** | **0.91** | **-** | **1.62** | **)** |
| **Mechanical complication due to other implant and internal device** | **79** | **avg_bmi** | **0.187** | **1.24** | **(** | **0.90** | **-** | **1.69** | **)** |
| **Uterine/Uterovaginal prolapse** | **129** | **avg_bmi** | **0.189** | **0.84** | **(** | **0.65** | **-** | **1.09** | **)** |
| **Empyema and pneumothorax** | **89** | **avg_bmi** | **0.190** | **0.81** | **(** | **0.59** | **-** | **1.11** | **)** |
| **Pathological, developmental or recurrent dislocation** | **21** | **avg_bmi** | **0.191** | **1.50** | **(** | **0.82** | **-** | **2.75** | **)** |
| **Chorioretinal scars** | **50** | **avg_bmi** | **0.191** | **1.30** | **(** | **0.88** | **-** | **1.94** | **)** |
| **Polyneuropathy in diabetes** | **270** | **avg_bmi** | **0.192** | **1.13** | **(** | **0.94** | **-** | **1.35** | **)** |
| **Schizophrenia and other psychotic disorders** | **225** | **avg_bmi** | **0.192** | **1.14** | **(** | **0.93** | **-** | **1.40** | **)** |
| **Acquired spondylolisthesis** | **123** | **avg_bmi** | **0.193** | **1.18** | **(** | **0.92** | **-** | **1.53** | **)** |
| **Hematuria** | **911** | **avg_bmi** | **0.194** | **1.07** | **(** | **0.97** | **-** | **1.19** | **)** |
| **Stricture of artery** | **165** | **avg_bmi** | **0.195** | **1.16** | **(** | **0.93** | **-** | **1.46** | **)** |
| **Hemangioma of skin & subcutaneous tissue** | **63** | **avg_bmi** | **0.196** | **1.26** | **(** | **0.89** | **-** | **1.80** | **)** |
| **Dermatophytosis / Dermatomycosis** | **995** | **avg_bmi** | **0.198** | **0.93** | **(** | **0.84** | **-** | **1.04** | **)** |
| **Bundle branch block** | **338** | **avg_bmi** | **0.198** | **1.11** | **(** | **0.95** | **-** | **1.31** | **)** |
| **Symptoms/disorders of the urinary system** | **2349** | **avg_bmi** | **0.198** | **1.05** | **(** | **0.97** | **-** | **1.14** | **)** |
| **Congenital anomalies of urinary system** | **44** | **avg_bmi** | **0.199** | **0.75** | **(** | **0.48** | **-** | **1.16** | **)** |
| **Symptoms involving head and neck** | **1169** | **avg_bmi** | **0.200** | **1.07** | **(** | **0.97** | **-** | **1.17** | **)** |
| **Hypocalcemia** | **34** | **avg_bmi** | **0.201** | **1.37** | **(** | **0.85** | **-** | **2.21** | **)** |
| **Primary thrombocytopenia** | **46** | **avg_bmi** | **0.201** | **0.75** | **(** | **0.49** | **-** | **1.16** | **)** |
| **Hypercalcemia** | **172** | **avg_bmi** | **0.201** | **0.86** | **(** | **0.69** | **-** | **1.08** | **)** |
| **Seborrheic keratosis** | **1542** | **avg_bmi** | **0.202** | **1.06** | **(** | **0.97** | **-** | **1.16** | **)** |
| **Hemorrhage of gastrointestinal tract** | **310** | **avg_bmi** | **0.202** | **1.12** | **(** | **0.94** | **-** | **1.33** | **)** |
| **Disorders of conjunctiva** | **169** | **avg_bmi** | **0.203** | **1.16** | **(** | **0.92** | **-** | **1.45** | **)** |
| **Ascites (non malignant)** | **57** | **avg_bmi** | **0.203** | **1.27** | **(** | **0.88** | **-** | **1.84** | **)** |
| **Corneal degenerations** | **66** | **avg_bmi** | **0.203** | **1.25** | **(** | **0.89** | **-** | **1.77** | **)** |
| **Abnormal loss of weight and underweight** | **76** | **avg_bmi** | **0.204** | **1.24** | **(** | **0.89** | **-** | **1.73** | **)** |
| **Genital prolapse** | **360** | **avg_bmi** | **0.205** | **0.90** | **(** | **0.77** | **-** | **1.06** | **)** |
| **Postoperative infection** | **217** | **avg_bmi** | **0.205** | **1.13** | **(** | **0.93** | **-** | **1.37** | **)** |
| **Gastric ulcer** | **130** | **avg_bmi** | **0.207** | **0.84** | **(** | **0.65** | **-** | **1.10** | **)** |
| **Osteomyelitis** | **133** | **avg_bmi** | **0.207** | **1.17** | **(** | **0.92** | **-** | **1.49** | **)** |
| **Althete's foot** | **134** | **avg_bmi** | **0.207** | **0.85** | **(** | **0.66** | **-** | **1.09** | **)** |
| **Fracture of upper limb** | **461** | **avg_bmi** | **0.207** | **1.09** | **(** | **0.95** | **-** | **1.26** | **)** |
| **Acquired deformities of ankle and foot** | **105** | **avg_bmi** | **0.207** | **0.83** | **(** | **0.62** | **-** | **1.11** | **)** |
| **Vaginal enterocele, congenital or acquired** | **58** | **avg_bmi** | **0.208** | **0.78** | **(** | **0.53** | **-** | **1.15** | **)** |
| **Hereditary hemolytic anemias** | **26** | **avg_bmi** | **0.208** | **1.42** | **(** | **0.82** | **-** | **2.46** | **)** |
| **Open wounds of extremities** | **687** | **avg_bmi** | **0.209** | **1.08** | **(** | **0.96** | **-** | **1.22** | **)** |
| **Other endocrine disorders** | **29** | **avg_bmi** | **0.210** | **1.39** | **(** | **0.83** | **-** | **2.33** | **)** |
| **Hemorrhage of rectum and anus** | **229** | **avg_bmi** | **0.213** | **1.13** | **(** | **0.93** | **-** | **1.37** | **)** |
| **Obesity** | **317** | **no_bmi** | **1.88E-04** | **1.37** | **(** | **1.16** | **-** | **1.61** | **)** |
| **Morbid obesity** | **317** | **no_bmi** | **1.88E-04** | **1.37** | **(** | **1.16** | **-** | **1.61** | **)** |
| **End stage renal disease** | **57** | **no_bmi** | **2.49E-04** | **2.01** | **(** | **1.38** | **-** | **2.92** | **)** |
| **Chronic ulcer of leg or foot** | **440** | **no_bmi** | **8.12E-04** | **1.27** | **(** | **1.10** | **-** | **1.45** | **)** |
| **Chronic ulcer of skin** | **611** | **no_bmi** | **1.53E-03** | **1.21** | **(** | **1.08** | **-** | **1.37** | **)** |
| **Sleep apnea** | **1393** | **no_bmi** | **2.41E-03** | **1.14** | **(** | **1.05** | **-** | **1.24** | **)** |
| **Emphysema** | **128** | **no_bmi** | **4.20E-03** | **0.68** | **(** | **0.52** | **-** | **0.88** | **)** |
| **Cystic mastopathy** | **518** | **no_bmi** | **5.59E-03** | **0.81** | **(** | **0.69** | **-** | **0.94** | **)** |
| **Other disorders of intestine** | **1301** | **no_bmi** | **6.70E-03** | **1.14** | **(** | **1.04** | **-** | **1.26** | **)** |
| **Abnormal results of function study of liver** | **179** | **no_bmi** | **7.07E-03** | **1.34** | **(** | **1.08** | **-** | **1.65** | **)** |
| **Disturbances of sensation of smell and taste** | **24** | **no_bmi** | **9.68E-03** | **2.15** | **(** | **1.20** | **-** | **3.83** | **)** |
| **Abnormal cytological, histological, immunological and DNA test findings** | **169** | **no_bmi** | **0.011** | **1.34** | **(** | **1.07** | **-** | **1.69** | **)** |
| **Chronic nonalcoholic liver disease** | **221** | **no_bmi** | **0.011** | **1.28** | **(** | **1.06** | **-** | **1.55** | **)** |
| **Corns and callosities** | **364** | **no_bmi** | **0.012** | **1.22** | **(** | **1.04** | **-** | **1.43** | **)** |
| **Retinal edema and hypertensive retinopathy** | **107** | **no_bmi** | **0.013** | **1.41** | **(** | **1.07** | **-** | **1.85** | **)** |
| **Type 2 diabetes** | **630** | **no_bmi** | **0.014** | **1.16** | **(** | **1.03** | **-** | **1.32** | **)** |
| **Fracture of lower limb** | **375** | **no_bmi** | **0.014** | **1.21** | **(** | **1.04** | **-** | **1.41** | **)** |
| **Spinal stenosis of lumbar region** | **456** | **no_bmi** | **0.015** | **1.19** | **(** | **1.03** | **-** | **1.36** | **)** |
| **Arterial embolism and thrombosis of lower extremity artery** | **108** | **no_bmi** | **0.016** | **1.40** | **(** | **1.07** | **-** | **1.84** | **)** |
| **Acute renal failure** | **305** | **no_bmi** | **0.017** | **1.22** | **(** | **1.04** | **-** | **1.44** | **)** |
| **Other hypertrophic and atrophic conditions of skin** | **1045** | **no_bmi** | **0.017** | **1.13** | **(** | **1.02** | **-** | **1.25** | **)** |
| **Osteoarthrosis NOS** | **2286** | **no_bmi** | **0.018** | **1.11** | **(** | **1.02** | **-** | **1.21** | **)** |
| **Diverticulitis** | **202** | **no_bmi** | **0.018** | **1.28** | **(** | **1.04** | **-** | **1.57** | **)** |
| **Chronic liver disease and cirrhosis** | **258** | **no_bmi** | **0.019** | **1.24** | **(** | **1.04** | **-** | **1.48** | **)** |
| **Spinal stenosis** | **475** | **no_bmi** | **0.019** | **1.18** | **(** | **1.03** | **-** | **1.35** | **)** |
| **Other specified diseases of sebaceous glands** | **160** | **no_bmi** | **0.020** | **0.75** | **(** | **0.59** | **-** | **0.96** | **)** |
| **Diabetes mellitus** | **3379** | **no_bmi** | **0.020** | **1.08** | **(** | **1.01** | **-** | **1.16** | **)** |
| **Benign mammary dysplasias** | **654** | **no_bmi** | **0.020** | **0.85** | **(** | **0.74** | **-** | **0.98** | **)** |
| **Chronic venous insufficiency** | **271** | **no_bmi** | **0.022** | **1.24** | **(** | **1.03** | **-** | **1.49** | **)** |
| **Behcet's syndrome** | **48** | **no_bmi** | **0.022** | **0.60** | **(** | **0.39** | **-** | **0.93** | **)** |
| **Influenza** | **42** | **no_bmi** | **0.022** | **0.57** | **(** | **0.35** | **-** | **0.92** | **)** |
| **Thoracic neuritis/radiculitis** | **634** | **no_bmi** | **0.023** | **1.15** | **(** | **1.02** | **-** | **1.30** | **)** |
| **Anemia of chronic disease** | **202** | **no_bmi** | **0.023** | **1.26** | **(** | **1.03** | **-** | **1.54** | **)** |
| **Eating disorder** | **52** | **no_bmi** | **0.023** | **1.58** | **(** | **1.07** | **-** | **2.34** | **)** |
| **Immunity deficiency** | **26** | **no_bmi** | **0.023** | **1.88** | **(** | **1.09** | **-** | **3.23** | **)** |
| **Viral warts & HPV** | **425** | **no_bmi** | **0.024** | **1.18** | **(** | **1.02** | **-** | **1.36** | **)** |
| **Gastrointestinal complications** | **942** | **no_bmi** | **0.024** | **1.16** | **(** | **1.02** | **-** | **1.31** | **)** |
| **Chronic tonsillitis and adenoiditis** | **27** | **no_bmi** | **0.025** | **1.86** | **(** | **1.08** | **-** | **3.19** | **)** |
| **Chronic ulcer of unspecified site** | **123** | **no_bmi** | **0.026** | **1.33** | **(** | **1.03** | **-** | **1.71** | **)** |
| **Other disorders of peritoneum** | **53** | **no_bmi** | **0.027** | **1.54** | **(** | **1.05** | **-** | **2.26** | **)** |
| **Scar conditions and fibrosis of skin** | **106** | **no_bmi** | **0.027** | **1.36** | **(** | **1.04** | **-** | **1.78** | **)** |
| **Obstruction of bile duct** | **32** | **no_bmi** | **0.027** | **0.54** | **(** | **0.31** | **-** | **0.93** | **)** |
| **Intestinal malabsorption NOS** | **22** | **no_bmi** | **0.028** | **1.94** | **(** | **1.08** | **-** | **3.50** | **)** |
| **Abnormal findings on radiological examination intrathoracic organs** | **119** | **no_bmi** | **0.030** | **1.35** | **(** | **1.03** | **-** | **1.76** | **)** |
| **Type 2 diabetic ketoacidosis** | **478** | **no_bmi** | **0.030** | **1.17** | **(** | **1.01** | **-** | **1.34** | **)** |
| **Ventral hernia** | **141** | **no_bmi** | **0.031** | **1.30** | **(** | **1.02** | **-** | **1.64** | **)** |
| **Other abnormality of urination** | **219** | **no_bmi** | **0.032** | **1.23** | **(** | **1.02** | **-** | **1.49** | **)** |
| **Cholelithiasis with other cholecystitis** | **90** | **no_bmi** | **0.033** | **1.38** | **(** | **1.03** | **-** | **1.85** | **)** |
| **Anemia in chronic kidney disease** | **75** | **no_bmi** | **0.034** | **1.41** | **(** | **1.03** | **-** | **1.95** | **)** |
| **Osteoarthrosis** | **3347** | **no_bmi** | **0.037** | **1.08** | **(** | **1.01** | **-** | **1.17** | **)** |
| **Other disorders of testis** | **75** | **no_bmi** | **0.037** | **0.69** | **(** | **0.48** | **-** | **0.98** | **)** |
| **Other disorders of the nervous system** | **38** | **no_bmi** | **0.037** | **1.61** | **(** | **1.03** | **-** | **2.52** | **)** |
| **Second degree AV block** | **32** | **no_bmi** | **0.037** | **1.70** | **(** | **1.03** | **-** | **2.80** | **)** |
| **Peripheral retinal degenerations** | **105** | **no_bmi** | **0.039** | **1.34** | **(** | **1.01** | **-** | **1.76** | **)** |
| **Coronary atherosclerosis** | **1468** | **no_bmi** | **0.040** | **1.10** | **(** | **1.00** | **-** | **1.21** | **)** |
| **Cirrhosis of liver without mention of alcohol** | **80** | **no_bmi** | **0.040** | **1.39** | **(** | **1.01** | **-** | **1.91** | **)** |
| **Macular puckering of retina** | **227** | **no_bmi** | **0.041** | **1.22** | **(** | **1.01** | **-** | **1.48** | **)** |
| **Disorders of synovium, tendon, and bursa** | **1114** | **no_bmi** | **0.042** | **1.11** | **(** | **1.00** | **-** | **1.23** | **)** |
| **Other specified cardiac dysrhythmias** | **788** | **no_bmi** | **0.042** | **1.13** | **(** | **1.00** | **-** | **1.28** | **)** |
| **Arthralgia/ankylosis of temporomandibular joint** | **29** | **no_bmi** | **0.042** | **1.72** | **(** | **1.02** | **-** | **2.90** | **)** |
| **Superficial cellulitis and abscess** | **1298** | **no_bmi** | **0.043** | **1.10** | **(** | **1.00** | **-** | **1.20** | **)** |
| **Other aneurysm** | **865** | **no_bmi** | **0.043** | **0.89** | **(** | **0.79** | **-** | **1.00** | **)** |
| **Decubitus ulcer** | **112** | **no_bmi** | **0.043** | **1.31** | **(** | **1.01** | **-** | **1.71** | **)** |
| **Chronic periodontitis** | **202** | **no_bmi** | **0.045** | **0.80** | **(** | **0.65** | **-** | **0.99** | **)** |
| **Displacement of intervertebral disc** | **291** | **no_bmi** | **0.045** | **1.19** | **(** | **1.00** | **-** | **1.41** | **)** |
| **Irritable Bowel Syndrome** | **365** | **no_bmi** | **0.047** | **1.18** | **(** | **1.00** | **-** | **1.38** | **)** |
| **Allergic conjunctivitis** | **119** | **no_bmi** | **0.049** | **1.31** | **(** | **1.00** | **-** | **1.72** | **)** |
| **Myeloproliferative disease** | **86** | **no_bmi** | **0.051** | **0.73** | **(** | **0.53** | **-** | **1.00** | **)** |
| **Lipoma of skin & subcutaneous tissue** | **114** | **no_bmi** | **0.051** | **0.75** | **(** | **0.57** | **-** | **1.00** | **)** |
| **Chronic renal failure** | **894** | **no_bmi** | **0.053** | **1.11** | **(** | **1.00** | **-** | **1.23** | **)** |
| **Calculus of bile duct** | **86** | **no_bmi** | **0.053** | **0.73** | **(** | **0.53** | **-** | **1.00** | **)** |
| **Testicular dysfunction** | **91** | **no_bmi** | **0.053** | **0.73** | **(** | **0.54** | **-** | **1.00** | **)** |
| **Obstructive chronic bronchitis** | **188** | **no_bmi** | **0.055** | **0.81** | **(** | **0.65** | **-** | **1.00** | **)** |
| **Fracture of humerus** | **144** | **no_bmi** | **0.056** | **1.26** | **(** | **0.99** | **-** | **1.60** | **)** |
| **Other cells and casts in urine** | **75** | **no_bmi** | **0.057** | **1.42** | **(** | **0.99** | **-** | **2.03** | **)** |
| **Conjunctivitis, infectious** | **235** | **no_bmi** | **0.058** | **1.21** | **(** | **0.99** | **-** | **1.47** | **)** |
| **Temporomandibular joint disorders** | **117** | **no_bmi** | **0.059** | **1.29** | **(** | **0.99** | **-** | **1.68** | **)** |
| **Cellulitis and abscess of foot/toes** | **117** | **no_bmi** | **0.059** | **1.29** | **(** | **0.99** | **-** | **1.67** | **)** |
| **Hemoptysis** | **76** | **no_bmi** | **0.059** | **0.72** | **(** | **0.51** | **-** | **1.01** | **)** |
| **Appendiceal conditions** | **110** | **no_bmi** | **0.059** | **0.76** | **(** | **0.57** | **-** | **1.01** | **)** |
| **Elevated levels of transaminase or lactic acid dehydrogenase** | **180** | **no_bmi** | **0.060** | **1.24** | **(** | **0.99** | **-** | **1.54** | **)** |
| **Angina pectoris** | **627** | **no_bmi** | **0.061** | **1.13** | **(** | **0.99** | **-** | **1.28** | **)** |
| **Muscular wasting and disuse atrophy** | **33** | **no_bmi** | **0.062** | **0.61** | **(** | **0.36** | **-** | **1.03** | **)** |
| **Elevated white blood cell count** | **101** | **no_bmi** | **0.063** | **1.30** | **(** | **0.99** | **-** | **1.73** | **)** |
| **Lipoma** | **247** | **no_bmi** | **0.064** | **0.84** | **(** | **0.69** | **-** | **1.01** | **)** |
| **Cancer of the upper aerodigestive tract** | **49** | **no_bmi** | **0.065** | **0.67** | **(** | **0.44** | **-** | **1.03** | **)** |
| **Polycythemia vera** | **23** | **no_bmi** | **0.066** | **0.54** | **(** | **0.28** | **-** | **1.04** | **)** |
| **Esophageal cancer** | **33** | **no_bmi** | **0.066** | **1.59** | **(** | **0.97** | **-** | **2.61** | **)** |
| **Noninflammatory disorders of vagina** | **85** | **no_bmi** | **0.068** | **1.33** | **(** | **0.98** | **-** | **1.81** | **)** |
| **Periodontitis (acute or chronic)** | **227** | **no_bmi** | **0.068** | **0.83** | **(** | **0.68** | **-** | **1.01** | **)** |
| **Cellulitis and abscess of leg** | **304** | **no_bmi** | **0.069** | **1.17** | **(** | **0.99** | **-** | **1.38** | **)** |
| **Urinary obstruction** | **40** | **no_bmi** | **0.070** | **0.64** | **(** | **0.40** | **-** | **1.04** | **)** |
| **Other open wound of head and face** | **164** | **no_bmi** | **0.070** | **1.23** | **(** | **0.98** | **-** | **1.54** | **)** |
| **Acute periodontitis** | **27** | **no_bmi** | **0.074** | **0.58** | **(** | **0.32** | **-** | **1.05** | **)** |
| **Lichen** | **48** | **no_bmi** | **0.075** | **1.44** | **(** | **0.96** | **-** | **2.14** | **)** |
| **Hemangioma and lymphangioma, any site** | **116** | **no_bmi** | **0.075** | **1.27** | **(** | **0.98** | **-** | **1.64** | **)** |
| **Conjunctivitis, noninfectious** | **148** | **no_bmi** | **0.076** | **1.25** | **(** | **0.98** | **-** | **1.60** | **)** |
| **Nonrheumatic aortic valve disorders** | **472** | **no_bmi** | **0.077** | **0.88** | **(** | **0.77** | **-** | **1.01** | **)** |
| **Other congenital anomalies of skin** | **41** | **no_bmi** | **0.077** | **1.48** | **(** | **0.96** | **-** | **2.27** | **)** |
| **Hypoventilation** | **29** | **no_bmi** | **0.077** | **1.61** | **(** | **0.95** | **-** | **2.73** | **)** |
| **Breast conditions, congenital or relating to hormones** | **86** | **no_bmi** | **0.077** | **1.31** | **(** | **0.97** | **-** | **1.78** | **)** |
| **Cancer, suspected or other** | **173** | **no_bmi** | **0.078** | **1.21** | **(** | **0.98** | **-** | **1.50** | **)** |
| **Hypotension NOS** | **175** | **no_bmi** | **0.081** | **1.21** | **(** | **0.98** | **-** | **1.50** | **)** |
| **Urethral stricture (not specified as infectious)** | **53** | **no_bmi** | **0.081** | **0.70** | **(** | **0.46** | **-** | **1.05** | **)** |
| **Testicular hypofunction** | **83** | **no_bmi** | **0.081** | **0.75** | **(** | **0.54** | **-** | **1.04** | **)** |
| **Hydrocele** | **47** | **no_bmi** | **0.081** | **0.67** | **(** | **0.43** | **-** | **1.05** | **)** |
| **Adverse drug events and drug allergies** | **237** | **no_bmi** | **0.082** | **1.18** | **(** | **0.98** | **-** | **1.42** | **)** |
| **Polyneuropathy in diabetes** | **270** | **no_bmi** | **0.083** | **1.17** | **(** | **0.98** | **-** | **1.40** | **)** |
| **Retinal drusen** | **199** | **no_bmi** | **0.083** | **1.21** | **(** | **0.98** | **-** | **1.49** | **)** |
| **Gastroparesis** | **25** | **no_bmi** | **0.084** | **1.64** | **(** | **0.94** | **-** | **2.86** | **)** |
| **Disturbances of amino-acid transport** | **29** | **no_bmi** | **0.084** | **1.58** | **(** | **0.94** | **-** | **2.67** | **)** |
| **Disturbances of sulphur-bearing amino-acid metabolism** | **29** | **no_bmi** | **0.084** | **1.58** | **(** | **0.94** | **-** | **2.67** | **)** |
| **Vascular insufficiency of intestine** | **79** | **no_bmi** | **0.085** | **1.32** | **(** | **0.96** | **-** | **1.80** | **)** |
| **Dermatophytosis of the body** | **84** | **no_bmi** | **0.085** | **1.31** | **(** | **0.96** | **-** | **1.79** | **)** |
| **Endometrial hyperplasia** | **42** | **no_bmi** | **0.085** | **1.46** | **(** | **0.95** | **-** | **2.24** | **)** |
| **Spondylosis with myelopathy** | **73** | **no_bmi** | **0.086** | **1.33** | **(** | **0.96** | **-** | **1.85** | **)** |
| **Diseases of white blood cells** | **354** | **no_bmi** | **0.087** | **1.14** | **(** | **0.98** | **-** | **1.33** | **)** |
| **Atrial fibrillation & flutter** | **1003** | **no_bmi** | **0.088** | **1.10** | **(** | **0.99** | **-** | **1.23** | **)** |
| **Fracture of tibia and fibula** | **107** | **no_bmi** | **0.088** | **1.27** | **(** | **0.97** | **-** | **1.67** | **)** |
| **Duodenitis** | **86** | **no_bmi** | **0.089** | **1.31** | **(** | **0.96** | **-** | **1.79** | **)** |
| **Optic neuritis/neuropathy** | **81** | **no_bmi** | **0.090** | **0.75** | **(** | **0.54** | **-** | **1.05** | **)** |
| **Fracture of neck of femur** | **204** | **no_bmi** | **0.091** | **1.20** | **(** | **0.97** | **-** | **1.47** | **)** |
| **Respiratory abnormalities** | **65** | **no_bmi** | **0.091** | **1.36** | **(** | **0.95** | **-** | **1.93** | **)** |
| **Keloid scar** | **26** | **no_bmi** | **0.091** | **1.61** | **(** | **0.93** | **-** | **2.80** | **)** |
| **Other disorders of biliary tract** | **46** | **no_bmi** | **0.091** | **0.69** | **(** | **0.44** | **-** | **1.06** | **)** |
| **Somatoform disorder** | **64** | **no_bmi** | **0.091** | **1.36** | **(** | **0.95** | **-** | **1.94** | **)** |
| **Immune disorders** | **117** | **no_bmi** | **0.094** | **1.25** | **(** | **0.96** | **-** | **1.62** | **)** |
| **Sciatica** | **269** | **no_bmi** | **0.095** | **1.17** | **(** | **0.97** | **-** | **1.41** | **)** |
| **Cardiac complications, not elsewhere classified** | **52** | **no_bmi** | **0.099** | **0.70** | **(** | **0.46** | **-** | **1.07** | **)** |
| **Protein plasma/amino-acid transport and metabolism disorder** | **150** | **no_bmi** | **0.099** | **1.21** | **(** | **0.96** | **-** | **1.53** | **)** |
| **Open wound of hand except finger(s)** | **87** | **no_bmi** | **0.100** | **1.30** | **(** | **0.95** | **-** | **1.76** | **)** |
| **Cerebral aneurysm** | **32** | **no_bmi** | **0.100** | **0.64** | **(** | **0.37** | **-** | **1.09** | **)** |
| **Atrophic gastritis** | **56** | **no_bmi** | **0.101** | **1.37** | **(** | **0.94** | **-** | **2.00** | **)** |
| **Edema** | **895** | **no_bmi** | **0.102** | **1.09** | **(** | **0.98** | **-** | **1.22** | **)** |
| **Prolapse of vaginal vault after hysterectomy** | **53** | **no_bmi** | **0.102** | **0.71** | **(** | **0.46** | **-** | **1.07** | **)** |
| **Psychogenic disorder** | **32** | **no_bmi** | **0.103** | **0.63** | **(** | **0.36** | **-** | **1.10** | **)** |
| **Acute and chronic tonsillitis** | **56** | **no_bmi** | **0.103** | **1.38** | **(** | **0.94** | **-** | **2.02** | **)** |
| **Postoperative infection** | **217** | **no_bmi** | **0.104** | **1.17** | **(** | **0.97** | **-** | **1.42** | **)** |
| **Herpes zoster with nervous system complications** | **40** | **no_bmi** | **0.104** | **1.44** | **(** | **0.93** | **-** | **2.23** | **)** |
| **Alcoholism** | **78** | **no_bmi** | **0.105** | **1.31** | **(** | **0.95** | **-** | **1.81** | **)** |
| **Other alveolar and parietoalveolar pneumonopathy** | **34** | **no_bmi** | **0.105** | **1.48** | **(** | **0.92** | **-** | **2.38** | **)** |
| **Hypotension** | **472** | **no_bmi** | **0.106** | **1.12** | **(** | **0.98** | **-** | **1.28** | **)** |
| **Thyrotoxicosis** | **128** | **no_bmi** | **0.106** | **1.23** | **(** | **0.96** | **-** | **1.58** | **)** |
| **Sepsis** | **79** | **no_bmi** | **0.106** | **1.30** | **(** | **0.95** | **-** | **1.78** | **)** |
| **Neurological disorders due to brain damage** | **560** | **no_bmi** | **0.107** | **0.90** | **(** | **0.78** | **-** | **1.02** | **)** |
| **Disorders of iris and ciliary body** | **116** | **no_bmi** | **0.107** | **0.80** | **(** | **0.61** | **-** | **1.05** | **)** |
| **Crystal arthropathies** | **75** | **no_bmi** | **0.107** | **1.31** | **(** | **0.94** | **-** | **1.81** | **)** |
| **Varicose veins of lower extremity, symptomtic** | **183** | **no_bmi** | **0.109** | **1.19** | **(** | **0.96** | **-** | **1.47** | **)** |
| **Pneumonia** | **1089** | **no_bmi** | **0.110** | **0.92** | **(** | **0.83** | **-** | **1.02** | **)** |
| **Asthma** | **886** | **no_bmi** | **0.111** | **1.09** | **(** | **0.98** | **-** | **1.20** | **)** |
| **Purpura and other hemorrhagic conditions** | **365** | **no_bmi** | **0.112** | **0.88** | **(** | **0.76** | **-** | **1.03** | **)** |
| **Insomnia** | **470** | **no_bmi** | **0.113** | **1.12** | **(** | **0.97** | **-** | **1.28** | **)** |
| **Diseases of hard tissues of teeth** | **269** | **no_bmi** | **0.113** | **0.87** | **(** | **0.72** | **-** | **1.03** | **)** |
| **Cramp of limb** | **102** | **no_bmi** | **0.113** | **1.25** | **(** | **0.95** | **-** | **1.65** | **)** |
| **Fever of unknown origin** | **568** | **no_bmi** | **0.113** | **1.11** | **(** | **0.98** | **-** | **1.25** | **)** |
| **Congenital anomalies of great vessels** | **21** | **no_bmi** | **0.114** | **1.63** | **(** | **0.89** | **-** | **2.97** | **)** |
| **Pain in limb** | **2292** | **no_bmi** | **0.114** | **1.07** | **(** | **0.98** | **-** | **1.16** | **)** |
| **Infection of the eye** | **623** | **no_bmi** | **0.115** | **1.11** | **(** | **0.97** | **-** | **1.27** | **)** |
| **Atrial fibrillation** | **896** | **no_bmi** | **0.115** | **1.10** | **(** | **0.98** | **-** | **1.23** | **)** |
| **Nonallopathic lesions NEC** | **188** | **no_bmi** | **0.117** | **0.84** | **(** | **0.68** | **-** | **1.04** | **)** |
| **Hypertrophy of breast (Gynecomastia)** | **70** | **no_bmi** | **0.118** | **1.31** | **(** | **0.93** | **-** | **1.82** | **)** |
| **Nontoxic multinodular goiter** | **94** | **no_bmi** | **0.118** | **0.78** | **(** | **0.58** | **-** | **1.06** | **)** |
| **Atherosclerosis of native arteries of the extremities with ulceration or gangrene** | **184** | **no_bmi** | **0.118** | **1.19** | **(** | **0.96** | **-** | **1.47** | **)** |
| **Cardiac congenital anomalies** | **98** | **no_bmi** | **0.118** | **1.25** | **(** | **0.94** | **-** | **1.66** | **)** |
| **Secondary malignancy of lymph nodes** | **158** | **no_bmi** | **0.119** | **1.20** | **(** | **0.96** | **-** | **1.50** | **)** |
| **Failure to thrive** | **47** | **no_bmi** | **0.119** | **0.70** | **(** | **0.45** | **-** | **1.09** | **)** |
| **Genu valgum or varum (acquired)** | **44** | **no_bmi** | **0.120** | **1.40** | **(** | **0.92** | **-** | **2.15** | **)** |
| **Peripheral or central vertigo** | **225** | **no_bmi** | **0.120** | **1.16** | **(** | **0.96** | **-** | **1.41** | **)** |
| **Cellulitis and abscess of arm** | **147** | **no_bmi** | **0.121** | **1.20** | **(** | **0.95** | **-** | **1.52** | **)** |
| **Open wound of foot except toe(s) alone** | **32** | **no_bmi** | **0.123** | **0.66** | **(** | **0.39** | **-** | **1.12** | **)** |
| **Other anemias** | **1831** | **no_bmi** | **0.123** | **1.07** | **(** | **0.98** | **-** | **1.16** | **)** |
| **Heartburn** | **68** | **no_bmi** | **0.123** | **1.32** | **(** | **0.93** | **-** | **1.88** | **)** |
| **Osteoporosis, osteopenia, & pathological fractures** | **1159** | **no_bmi** | **0.124** | **0.93** | **(** | **0.84** | **-** | **1.02** | **)** |
| **Cervical cancer and dysplasia** | **119** | **no_bmi** | **0.124** | **1.23** | **(** | **0.95** | **-** | **1.60** | **)** |
| **Dermatophytosis of nail** | **564** | **no_bmi** | **0.125** | **0.90** | **(** | **0.78** | **-** | **1.03** | **)** |
| **Cervical intraepithelial neoplasia (Cervical dysplasia)** | **107** | **no_bmi** | **0.126** | **1.24** | **(** | **0.94** | **-** | **1.64** | **)** |
| **Brain cancer** | **30** | **no_bmi** | **0.126** | **1.48** | **(** | **0.90** | **-** | **2.44** | **)** |
| **Renal failure** | **1225** | **no_bmi** | **0.126** | **1.07** | **(** | **0.98** | **-** | **1.18** | **)** |
| **Cancer of brain and nervous system** | **30** | **no_bmi** | **0.126** | **1.48** | **(** | **0.90** | **-** | **2.44** | **)** |
| **Dysthymic disorder** | **315** | **no_bmi** | **0.129** | **1.14** | **(** | **0.96** | **-** | **1.35** | **)** |
| **Unspecified osteomyelitis** | **104** | **no_bmi** | **0.129** | **1.24** | **(** | **0.94** | **-** | **1.63** | **)** |
| **Paralytic ileus** | **77** | **no_bmi** | **0.129** | **1.28** | **(** | **0.93** | **-** | **1.77** | **)** |
| **Peptic ulcers** | **110** | **no_bmi** | **0.131** | **1.23** | **(** | **0.94** | **-** | **1.62** | **)** |
| **Other benign neoplasm of connective and other soft tissue** | **52** | **no_bmi** | **0.132** | **1.35** | **(** | **0.91** | **-** | **2.00** | **)** |
| **Inflammatory spondylopathies** | **37** | **no_bmi** | **0.132** | **1.43** | **(** | **0.90** | **-** | **2.26** | **)** |
| **Type 1 diabetic neuropathy** | **75** | **no_bmi** | **0.134** | **0.77** | **(** | **0.55** | **-** | **1.08** | **)** |
| **Deviated nasal septum** | **214** | **no_bmi** | **0.135** | **1.17** | **(** | **0.95** | **-** | **1.44** | **)** |
| **Cardiac pacemaker/device in situ** | **26** | **no_bmi** | **0.136** | **1.52** | **(** | **0.88** | **-** | **2.63** | **)** |
| **Mixed hyperlipidemia** | **467** | **no_bmi** | **0.138** | **1.12** | **(** | **0.96** | **-** | **1.31** | **)** |
| **Chronic sinusitis** | **798** | **no_bmi** | **0.139** | **1.09** | **(** | **0.97** | **-** | **1.23** | **)** |
| **Hypertensive chronic kidney disease** | **375** | **no_bmi** | **0.140** | **1.13** | **(** | **0.96** | **-** | **1.33** | **)** |
| **Other disorders of middle ear and mastoid** | **32** | **no_bmi** | **0.140** | **1.45** | **(** | **0.88** | **-** | **2.38** | **)** |
| **Chronic prostatitis** | **57** | **no_bmi** | **0.142** | **1.33** | **(** | **0.91** | **-** | **1.95** | **)** |
| **Atrial flutter** | **169** | **no_bmi** | **0.142** | **1.18** | **(** | **0.95** | **-** | **1.48** | **)** |
| **Bronchopneumonia and lung abscess** | **26** | **no_bmi** | **0.142** | **0.64** | **(** | **0.36** | **-** | **1.16** | **)** |
| **Abnormal Papanicolaou smear of cervix and cervical HPV** | **65** | **no_bmi** | **0.146** | **1.30** | **(** | **0.91** | **-** | **1.84** | **)** |
| **Arterial embolism and thrombosis** | **254** | **no_bmi** | **0.148** | **1.15** | **(** | **0.95** | **-** | **1.38** | **)** |
| **Abnormal findings on examination of urine** | **346** | **no_bmi** | **0.149** | **1.12** | **(** | **0.96** | **-** | **1.32** | **)** |
| **Other disorders of urethra and urinary tract** | **1575** | **no_bmi** | **0.149** | **1.07** | **(** | **0.98** | **-** | **1.17** | **)** |
| **Other specified erythematous conditions** | **75** | **no_bmi** | **0.150** | **1.27** | **(** | **0.92** | **-** | **1.75** | **)** |
| **Sepsis and SIRS** | **94** | **no_bmi** | **0.151** | **1.24** | **(** | **0.93** | **-** | **1.65** | **)** |
| **Complication of internal orthopedic device** | **191** | **no_bmi** | **0.151** | **0.86** | **(** | **0.70** | **-** | **1.06** | **)** |
| **Hypertensive heart and/or renal disease** | **587** | **no_bmi** | **0.154** | **1.10** | **(** | **0.96** | **-** | **1.26** | **)** |
| **Empyema and pneumothorax** | **89** | **no_bmi** | **0.154** | **0.80** | **(** | **0.58** | **-** | **1.09** | **)** |
| **Gross hematuria** | **38** | **no_bmi** | **0.154** | **1.40** | **(** | **0.88** | **-** | **2.22** | **)** |
| **Chondrocalcinosis** | **73** | **no_bmi** | **0.155** | **1.27** | **(** | **0.91** | **-** | **1.77** | **)** |
| **Heart failure** | **1295** | **no_bmi** | **0.155** | **1.07** | **(** | **0.97** | **-** | **1.18** | **)** |
| **Crohn's disease** | **72** | **no_bmi** | **0.155** | **0.78** | **(** | **0.55** | **-** | **1.10** | **)** |
| **Disorders of adrenal glands** | **88** | **no_bmi** | **0.156** | **1.24** | **(** | **0.92** | **-** | **1.67** | **)** |
| **Paralysis/spasm of vocal cords or larynx** | **28** | **no_bmi** | **0.159** | **1.46** | **(** | **0.86** | **-** | **2.48** | **)** |
| **Other acquired musculoskeletal deformity** | **167** | **no_bmi** | **0.160** | **1.17** | **(** | **0.94** | **-** | **1.46** | **)** |
| **Lymphadenitis** | **301** | **no_bmi** | **0.162** | **1.13** | **(** | **0.95** | **-** | **1.33** | **)** |
| **Methicillin sensitive Staphylococcus aureus** | **41** | **no_bmi** | **0.163** | **1.36** | **(** | **0.88** | **-** | **2.10** | **)** |
| **H. pylori** | **36** | **no_bmi** | **0.164** | **1.39** | **(** | **0.88** | **-** | **2.20** | **)** |
| **Chorioretinal scars** | **50** | **no_bmi** | **0.164** | **1.32** | **(** | **0.89** | **-** | **1.97** | **)** |
| **Other cardiac conduction disorders** | **102** | **no_bmi** | **0.165** | **1.22** | **(** | **0.92** | **-** | **1.62** | **)** |
| **Antisocial/borderline personality disorder** | **24** | **no_bmi** | **0.165** | **1.51** | **(** | **0.84** | **-** | **2.69** | **)** |
| **Hyperglyceridemia** | **286** | **no_bmi** | **0.166** | **1.14** | **(** | **0.95** | **-** | **1.37** | **)** |
| **Abnormal sputum** | **89** | **no_bmi** | **0.166** | **0.80** | **(** | **0.59** | **-** | **1.10** | **)** |
| **Alcoholic liver damage** | **28** | **no_bmi** | **0.167** | **1.45** | **(** | **0.86** | **-** | **2.47** | **)** |
| **Unspecified polyarthropathy or polyarthritis** | **30** | **no_bmi** | **0.170** | **0.68** | **(** | **0.40** | **-** | **1.18** | **)** |
| **Primary angle-closure glaucoma** | **185** | **no_bmi** | **0.170** | **1.17** | **(** | **0.93** | **-** | **1.47** | **)** |
| **Atherosclerosis of the extremities** | **1136** | **no_bmi** | **0.175** | **1.07** | **(** | **0.97** | **-** | **1.19** | **)** |
| **Chronic airway obstruction** | **1126** | **no_bmi** | **0.175** | **0.94** | **(** | **0.85** | **-** | **1.03** | **)** |
| **Hemorrhage of rectum and anus** | **229** | **no_bmi** | **0.176** | **1.14** | **(** | **0.94** | **-** | **1.39** | **)** |
| **Conduct disorders** | **22** | **no_bmi** | **0.177** | **1.51** | **(** | **0.83** | **-** | **2.75** | **)** |
| **Bundle branch block** | **338** | **no_bmi** | **0.177** | **1.12** | **(** | **0.95** | **-** | **1.32** | **)** |
| **Chronic bronchitis** | **250** | **no_bmi** | **0.177** | **0.88** | **(** | **0.73** | **-** | **1.06** | **)** |
| **AV block** | **438** | **no_bmi** | **0.179** | **1.11** | **(** | **0.95** | **-** | **1.29** | **)** |
| **Inflammatory bowel disease** | **135** | **no_bmi** | **0.179** | **0.84** | **(** | **0.66** | **-** | **1.08** | **)** |
| **Congenital anomalies of urinary system** | **44** | **no_bmi** | **0.181** | **0.74** | **(** | **0.48** | **-** | **1.15** | **)** |
| **Hemorrhage of gastrointestinal tract** | **310** | **no_bmi** | **0.181** | **1.13** | **(** | **0.95** | **-** | **1.34** | **)** |
| **Staphylococcus infections** | **102** | **no_bmi** | **0.182** | **1.21** | **(** | **0.92** | **-** | **1.59** | **)** |
| **Nasal polyps** | **76** | **no_bmi** | **0.184** | **1.25** | **(** | **0.90** | **-** | **1.73** | **)** |
| **Uterine/Uterovaginal prolapse** | **129** | **no_bmi** | **0.185** | **0.84** | **(** | **0.65** | **-** | **1.09** | **)** |
| **Hyposmolality and/or hyponatremia** | **338** | **no_bmi** | **0.185** | **1.12** | **(** | **0.95** | **-** | **1.31** | **)** |
| **Calcaneal spur; Exostosis NOS** | **119** | **no_bmi** | **0.186** | **1.20** | **(** | **0.92** | **-** | **1.58** | **)** |
| **Genital prolapse** | **360** | **no_bmi** | **0.187** | **0.90** | **(** | **0.77** | **-** | **1.05** | **)** |
| **Hyperlipidemia** | **4508** | **no_bmi** | **0.188** | **1.07** | **(** | **0.97** | **-** | **1.17** | **)** |
| **Hereditary hemolytic anemias** | **26** | **no_bmi** | **0.188** | **1.44** | **(** | **0.84** | **-** | **2.50** | **)** |
| **Dysmetabolic syndrome X** | **90** | **no_bmi** | **0.189** | **1.22** | **(** | **0.91** | **-** | **1.64** | **)** |
| **Impacted cerumen** | **939** | **no_bmi** | **0.189** | **0.93** | **(** | **0.84** | **-** | **1.04** | **)** |
| **Schizophrenia and other psychotic disorders** | **225** | **no_bmi** | **0.190** | **1.14** | **(** | **0.94** | **-** | **1.40** | **)** |
| **Premature menopause and other ovarian failure** | **24** | **no_bmi** | **0.191** | **1.46** | **(** | **0.83** | **-** | **2.58** | **)** |
| **Hypercalcemia** | **172** | **no_bmi** | **0.191** | **0.86** | **(** | **0.69** | **-** | **1.08** | **)** |
| **Corneal degenerations** | **66** | **no_bmi** | **0.191** | **1.26** | **(** | **0.89** | **-** | **1.78** | **)** |
| **Cancer of kidney and urinary organs** | **264** | **no_bmi** | **0.192** | **1.12** | **(** | **0.94** | **-** | **1.34** | **)** |
| **Eosinophilia** | **23** | **no_bmi** | **0.193** | **1.47** | **(** | **0.82** | **-** | **2.63** | **)** |
| **Osteomyelitis** | **133** | **no_bmi** | **0.193** | **1.17** | **(** | **0.92** | **-** | **1.50** | **)** |
| **Mechanical complication due to other implant and internal device** | **79** | **no_bmi** | **0.194** | **1.23** | **(** | **0.90** | **-** | **1.69** | **)** |

**†** Values are not corrected for multiple testing

| **BioVU** | | | | | | | | | |
| --- | --- | --- | --- | --- | --- | --- | --- | --- | --- |
|  |  |  |  |  |  |  |  |  |  |
| **PheWAS description** | **Cases** | **adjustment** | **p†** | **OR** | **95% CI** | | | | |
| **Type 2 diabetic ketoacidosis** | **1639** | **avg_bmi** | **3.54E-03** | **1.12** | **(** | **1.04** | **-** | **1.22** | **)** |
| **Joint effusions** | **195** | **avg_bmi** | **3.75E-03** | **1.34** | **(** | **1.10** | **-** | **1.64** | **)** |
| **Obstruction of bile duct** | **111** | **avg_bmi** | **3.98E-03** | **1.48** | **(** | **1.13** | **-** | **1.93** | **)** |
| **Diseases of pulp and periapical tissues** | **49** | **avg_bmi** | **4.26E-03** | **0.52** | **(** | **0.33** | **-** | **0.81** | **)** |
| **Proteinuria** | **352** | **avg_bmi** | **5.04E-03** | **0.80** | **(** | **0.68** | **-** | **0.93** | **)** |
| **Myalgia and myositis NOS** | **729** | **avg_bmi** | **5.04E-03** | **1.17** | **(** | **1.05** | **-** | **1.30** | **)** |
| **Rosacea** | **225** | **avg_bmi** | **5.56E-03** | **0.76** | **(** | **0.62** | **-** | **0.92** | **)** |
| **Vascular hamartomas and non-neoplastic nevi** | **69** | **avg_bmi** | **6.26E-03** | **0.60** | **(** | **0.41** | **-** | **0.86** | **)** |
| **Periapical abscess** | **48** | **avg_bmi** | **6.41E-03** | **0.53** | **(** | **0.34** | **-** | **0.84** | **)** |
| **Cystoid macular degeneration of retina** | **111** | **avg_bmi** | **7.51E-03** | **1.43** | **(** | **1.10** | **-** | **1.87** | **)** |
| **Anterior pituitary disorders** | **96** | **avg_bmi** | **7.52E-03** | **0.66** | **(** | **0.49** | **-** | **0.89** | **)** |
| **Abnormal mammogram** | **870** | **avg_bmi** | **7.82E-03** | **0.87** | **(** | **0.78** | **-** | **0.96** | **)** |
| **Streptococcus infection** | **341** | **avg_bmi** | **8.02E-03** | **1.23** | **(** | **1.06** | **-** | **1.43** | **)** |
| **Decreased white blood cell count** | **634** | **avg_bmi** | **8.24E-03** | **1.17** | **(** | **1.04** | **-** | **1.31** | **)** |
| **Pervasive developmental disorders** | **51** | **avg_bmi** | **8.91E-03** | **0.55** | **(** | **0.35** | **-** | **0.86** | **)** |
| **Benign mammary dysplasias** | **526** | **avg_bmi** | **9.42E-03** | **0.84** | **(** | **0.73** | **-** | **0.96** | **)** |
| **Symptoms and disorders of the joints** | **660** | **avg_bmi** | **0.010** | **1.16** | **(** | **1.04** | **-** | **1.30** | **)** |
| **Benign neoplasm of thyroid glands** | **37** | **avg_bmi** | **0.011** | **1.82** | **(** | **1.15** | **-** | **2.89** | **)** |
| **Attention deficit hyperactivity disorder** | **43** | **avg_bmi** | **0.011** | **0.53** | **(** | **0.33** | **-** | **0.87** | **)** |
| **Acute osteomyelitis** | **105** | **avg_bmi** | **0.012** | **1.42** | **(** | **1.08** | **-** | **1.87** | **)** |
| **Staphylococcus infections** | **621** | **avg_bmi** | **0.012** | **1.16** | **(** | **1.03** | **-** | **1.30** | **)** |
| **Mitral valve stenosis and/or aortic valve stenosis** | **157** | **avg_bmi** | **0.014** | **1.33** | **(** | **1.06** | **-** | **1.66** | **)** |
| **Type 2 diabetes** | **3306** | **avg_bmi** | **0.015** | **1.08** | **(** | **1.02** | **-** | **1.15** | **)** |
| **Premature beats** | **216** | **avg_bmi** | **0.015** | **1.27** | **(** | **1.05** | **-** | **1.54** | **)** |
| **Mitral stenosis/insufficiency** | **61** | **avg_bmi** | **0.016** | **1.55** | **(** | **1.09** | **-** | **2.22** | **)** |
| **Gram positive septicemia** | **133** | **avg_bmi** | **0.016** | **1.35** | **(** | **1.06** | **-** | **1.72** | **)** |
| **Cystic mastopathy** | **449** | **avg_bmi** | **0.017** | **0.84** | **(** | **0.73** | **-** | **0.97** | **)** |
| **Chronic nonalcoholic liver disease** | **463** | **avg_bmi** | **0.017** | **1.18** | **(** | **1.03** | **-** | **1.34** | **)** |
| **Nontoxic uninodular goiter** | **216** | **avg_bmi** | **0.018** | **1.26** | **(** | **1.04** | **-** | **1.53** | **)** |
| **Retinal vascular changes and abnomalities** | **125** | **avg_bmi** | **0.018** | **1.35** | **(** | **1.05** | **-** | **1.74** | **)** |
| **Cancer of the digestive organs and peritoneum** | **115** | **avg_bmi** | **0.018** | **1.37** | **(** | **1.06** | **-** | **1.78** | **)** |
| **Anemia in chronic kidney disease** | **336** | **avg_bmi** | **0.019** | **0.82** | **(** | **0.70** | **-** | **0.97** | **)** |
| **Pituitary hypofunction** | **62** | **avg_bmi** | **0.019** | **0.63** | **(** | **0.43** | **-** | **0.93** | **)** |
| **Acute bronchitis and bronchiolitis** | **772** | **avg_bmi** | **0.020** | **1.13** | **(** | **1.02** | **-** | **1.26** | **)** |
| **Hemorrhage from gastrointestinal ulcer** | **61** | **avg_bmi** | **0.020** | **1.52** | **(** | **1.07** | **-** | **2.17** | **)** |
| **Nevus, non-neoplastic** | **54** | **avg_bmi** | **0.021** | **0.61** | **(** | **0.40** | **-** | **0.93** | **)** |
| **Other symptoms involving abdomen and pelvis** | **619** | **avg_bmi** | **0.021** | **0.87** | **(** | **0.77** | **-** | **0.98** | **)** |
| **Acute pericarditis** | **33** | **avg_bmi** | **0.022** | **1.77** | **(** | **1.09** | **-** | **2.87** | **)** |
| **Crystal arthropathies** | **41** | **avg_bmi** | **0.022** | **1.66** | **(** | **1.08** | **-** | **2.55** | **)** |
| **Benign neoplasm of respiratory and intrathoracic organs** | **70** | **avg_bmi** | **0.024** | **0.66** | **(** | **0.46** | **-** | **0.95** | **)** |
| **Cancer of connective tissue** | **169** | **avg_bmi** | **0.025** | **1.28** | **(** | **1.03** | **-** | **1.59** | **)** |
| **Neutropenia** | **542** | **avg_bmi** | **0.025** | **1.15** | **(** | **1.02** | **-** | **1.30** | **)** |
| **Disease of tricuspid valve** | **123** | **avg_bmi** | **0.025** | **1.34** | **(** | **1.04** | **-** | **1.72** | **)** |
| **Intracerebral hemorrhage** | **127** | **avg_bmi** | **0.025** | **1.33** | **(** | **1.04** | **-** | **1.70** | **)** |
| **Lipoma of skin & subcutaneous tissue** | **77** | **avg_bmi** | **0.026** | **1.43** | **(** | **1.04** | **-** | **1.97** | **)** |
| **Pericarditis** | **171** | **avg_bmi** | **0.026** | **1.28** | **(** | **1.03** | **-** | **1.58** | **)** |
| **Other diseases of the teeth and supporting structures** | **79** | **avg_bmi** | **0.027** | **0.68** | **(** | **0.48** | **-** | **0.96** | **)** |
| **Hypoglycemia** | **111** | **avg_bmi** | **0.027** | **1.35** | **(** | **1.04** | **-** | **1.77** | **)** |
| **Cirrhosis of liver without mention of alcohol** | **342** | **avg_bmi** | **0.027** | **1.19** | **(** | **1.02** | **-** | **1.39** | **)** |
| **Secondary malignant neoplasm of liver** | **249** | **avg_bmi** | **0.027** | **1.22** | **(** | **1.02** | **-** | **1.46** | **)** |
| **Astigmatism** | **82** | **avg_bmi** | **0.027** | **1.41** | **(** | **1.04** | **-** | **1.92** | **)** |
| **Acute renal failure** | **1742** | **avg_bmi** | **0.027** | **1.09** | **(** | **1.01** | **-** | **1.17** | **)** |
| **Disorders of the pituitary gland and its hypothalamic control** | **173** | **avg_bmi** | **0.027** | **0.78** | **(** | **0.62** | **-** | **0.97** | **)** |
| **Inflammatory disease of breast** | **41** | **avg_bmi** | **0.028** | **1.63** | **(** | **1.05** | **-** | **2.51** | **)** |
| **Ill-defined descriptions and complications of heart disease** | **755** | **avg_bmi** | **0.030** | **1.13** | **(** | **1.01** | **-** | **1.25** | **)** |
| **Myasthenia gravis** | **41** | **avg_bmi** | **0.030** | **1.61** | **(** | **1.05** | **-** | **2.48** | **)** |
| **Chronic rheumatic disease of the heart valves** | **330** | **avg_bmi** | **0.032** | **1.19** | **(** | **1.02** | **-** | **1.39** | **)** |
| **Cardiac dysrhythmias** | **4842** | **avg_bmi** | **0.032** | **1.07** | **(** | **1.01** | **-** | **1.14** | **)** |
| **Hypoparathyroidism** | **32** | **avg_bmi** | **0.032** | **1.71** | **(** | **1.05** | **-** | **2.80** | **)** |
| **Inguinal hernia** | **349** | **avg_bmi** | **0.033** | **0.84** | **(** | **0.72** | **-** | **0.99** | **)** |
| **Non-healing surgical wound** | **78** | **avg_bmi** | **0.035** | **0.70** | **(** | **0.50** | **-** | **0.97** | **)** |
| **Nontoxic nodular goiter** | **446** | **avg_bmi** | **0.036** | **1.16** | **(** | **1.01** | **-** | **1.33** | **)** |
| **Myocardial infarction** | **1152** | **avg_bmi** | **0.036** | **1.10** | **(** | **1.01** | **-** | **1.21** | **)** |
| **Localized adiposity** | **36** | **avg_bmi** | **0.037** | **1.63** | **(** | **1.03** | **-** | **2.58** | **)** |
| **Secondary malignancy of lung** | **352** | **avg_bmi** | **0.037** | **1.17** | **(** | **1.01** | **-** | **1.37** | **)** |
| **Raynaud's syndrome** | **77** | **avg_bmi** | **0.038** | **0.69** | **(** | **0.49** | **-** | **0.98** | **)** |
| **Inflammatory conditions of jaw** | **21** | **avg_bmi** | **0.038** | **0.47** | **(** | **0.23** | **-** | **0.96** | **)** |
| **Primary angle-closure glaucoma** | **75** | **avg_bmi** | **0.038** | **1.40** | **(** | **1.02** | **-** | **1.94** | **)** |
| **Unspecified osteomyelitis** | **248** | **avg_bmi** | **0.038** | **1.21** | **(** | **1.01** | **-** | **1.45** | **)** |
| **Respiratory insufficiency** | **901** | **avg_bmi** | **0.039** | **1.11** | **(** | **1.01** | **-** | **1.23** | **)** |
| **Peptic ulcer** | **326** | **avg_bmi** | **0.039** | **1.18** | **(** | **1.01** | **-** | **1.38** | **)** |
| **Chondrocalcinosis** | **40** | **avg_bmi** | **0.040** | **1.58** | **(** | **1.02** | **-** | **2.45** | **)** |
| **Giant cell arteritis** | **48** | **avg_bmi** | **0.040** | **0.63** | **(** | **0.41** | **-** | **0.98** | **)** |
| **Mental retardation** | **56** | **avg_bmi** | **0.040** | **0.65** | **(** | **0.44** | **-** | **0.98** | **)** |
| **Pancreatic cancer** | **135** | **avg_bmi** | **0.041** | **1.29** | **(** | **1.01** | **-** | **1.64** | **)** |
| **Diabetes mellitus** | **3957** | **avg_bmi** | **0.041** | **1.07** | **(** | **1.00** | **-** | **1.13** | **)** |
| **Cancer of other female genital organs** | **41** | **avg_bmi** | **0.041** | **1.57** | **(** | **1.02** | **-** | **2.43** | **)** |
| **Diseases of blood and blood-forming organs** | **259** | **avg_bmi** | **0.042** | **0.83** | **(** | **0.69** | **-** | **0.99** | **)** |
| **Breast cancer** | **547** | **avg_bmi** | **0.042** | **0.88** | **(** | **0.77** | **-** | **1.00** | **)** |
| **Benign neoplasm of uterus** | **187** | **avg_bmi** | **0.042** | **0.80** | **(** | **0.64** | **-** | **0.99** | **)** |
| **Arthropathy NOS involving multiple sites** | **35** | **avg_bmi** | **0.042** | **0.58** | **(** | **0.34** | **-** | **0.98** | **)** |
| **Pituitary hyperfunction** | **46** | **avg_bmi** | **0.043** | **0.63** | **(** | **0.41** | **-** | **0.99** | **)** |
| **Polyarteritis nodosa and allied conditions** | **171** | **avg_bmi** | **0.044** | **0.79** | **(** | **0.63** | **-** | **0.99** | **)** |
| **Squamous cell carcinoma** | **120** | **avg_bmi** | **0.045** | **1.30** | **(** | **1.01** | **-** | **1.68** | **)** |
| **Malignant neoplasm of renal pelvis** | **45** | **avg_bmi** | **0.045** | **1.53** | **(** | **1.01** | **-** | **2.31** | **)** |
| **Infection of the eye** | **5597** | **avg_bmi** | **0.045** | **1.07** | **(** | **1.00** | **-** | **1.14** | **)** |
| **Disorders of tooth development** | **40** | **avg_bmi** | **0.049** | **1.56** | **(** | **1.00** | **-** | **2.43** | **)** |
| **Open wound of hand except finger(s)** | **61** | **avg_bmi** | **0.050** | **0.68** | **(** | **0.46** | **-** | **1.00** | **)** |
| **Iron deficiency anemia secondary to blood loss** | **345** | **avg_bmi** | **0.050** | **1.17** | **(** | **1.00** | **-** | **1.36** | **)** |
| **Disorders of adrenal glands** | **285** | **avg_bmi** | **0.051** | **0.84** | **(** | **0.71** | **-** | **1.00** | **)** |
| **Chronic liver disease and cirrhosis** | **543** | **avg_bmi** | **0.052** | **1.13** | **(** | **1.00** | **-** | **1.28** | **)** |
| **Hemorrhage of gastrointestinal tract** | **535** | **avg_bmi** | **0.053** | **1.13** | **(** | **1.00** | **-** | **1.28** | **)** |
| **Edema** | **1796** | **avg_bmi** | **0.054** | **1.07** | **(** | **1.00** | **-** | **1.16** | **)** |
| **Disturbances in tooth eruption** | **38** | **avg_bmi** | **0.055** | **1.56** | **(** | **0.99** | **-** | **2.46** | **)** |
| **Obesity** | **1345** | **avg_bmi** | **0.055** | **1.10** | **(** | **1.00** | **-** | **1.21** | **)** |
| **Other disorders of adrenal glands** | **36** | **avg_bmi** | **0.055** | **0.61** | **(** | **0.37** | **-** | **1.01** | **)** |
| **Prostate cancer** | **512** | **avg_bmi** | **0.055** | **0.88** | **(** | **0.77** | **-** | **1.00** | **)** |
| **Nodular lymphoma** | **169** | **avg_bmi** | **0.056** | **0.80** | **(** | **0.64** | **-** | **1.01** | **)** |
| **Infusion and transfusion reaction** | **40** | **avg_bmi** | **0.056** | **1.53** | **(** | **0.99** | **-** | **2.38** | **)** |
| **Urethral hypermobility/ISD** | **78** | **avg_bmi** | **0.056** | **0.72** | **(** | **0.52** | **-** | **1.01** | **)** |
| **Subdural hemorrhage (injury)** | **81** | **avg_bmi** | **0.057** | **0.72** | **(** | **0.52** | **-** | **1.01** | **)** |
| **Chronic venous insufficiency** | **206** | **avg_bmi** | **0.057** | **1.21** | **(** | **0.99** | **-** | **1.47** | **)** |
| **Uterine leiomyoma** | **169** | **avg_bmi** | **0.060** | **0.80** | **(** | **0.64** | **-** | **1.01** | **)** |
| **Pathologic fracture** | **267** | **avg_bmi** | **0.060** | **1.18** | **(** | **0.99** | **-** | **1.41** | **)** |
| **Chronic pericarditis** | **42** | **avg_bmi** | **0.060** | **1.51** | **(** | **0.98** | **-** | **2.32** | **)** |
| **Renal failure** | **2941** | **avg_bmi** | **0.060** | **1.06** | **(** | **1.00** | **-** | **1.13** | **)** |
| **Renal cell carcinoma** | **256** | **avg_bmi** | **0.061** | **1.18** | **(** | **0.99** | **-** | **1.41** | **)** |
| **Throat pain** | **37** | **avg_bmi** | **0.061** | **1.55** | **(** | **0.98** | **-** | **2.46** | **)** |
| **Torsion dystonia** | **94** | **avg_bmi** | **0.061** | **1.32** | **(** | **0.99** | **-** | **1.76** | **)** |
| **Morbid obesity** | **439** | **avg_bmi** | **0.062** | **1.17** | **(** | **0.99** | **-** | **1.37** | **)** |
| **Peyronie's disease** | **21** | **avg_bmi** | **0.062** | **0.52** | **(** | **0.26** | **-** | **1.03** | **)** |
| **Neuralgia, neuritis, and radiculitis NOS** | **421** | **avg_bmi** | **0.062** | **1.14** | **(** | **0.99** | **-** | **1.31** | **)** |
| **Abnormal involuntary movements** | **291** | **avg_bmi** | **0.063** | **1.17** | **(** | **0.99** | **-** | **1.38** | **)** |
| **Cancer of bone & connective tissue** | **241** | **avg_bmi** | **0.064** | **1.19** | **(** | **0.99** | **-** | **1.42** | **)** |
| **Cancer of kidney and renal pelvis** | **262** | **avg_bmi** | **0.064** | **1.18** | **(** | **0.99** | **-** | **1.40** | **)** |
| **Nontoxic multinodular goiter** | **287** | **avg_bmi** | **0.064** | **1.17** | **(** | **0.99** | **-** | **1.39** | **)** |
| **Diffuse diseases of connective tissue** | **225** | **avg_bmi** | **0.066** | **0.83** | **(** | **0.68** | **-** | **1.01** | **)** |
| **Myoclonus** | **50** | **avg_bmi** | **0.066** | **1.44** | **(** | **0.98** | **-** | **2.14** | **)** |
| **Antisocial/borderline personality disorder** | **37** | **avg_bmi** | **0.067** | **0.62** | **(** | **0.38** | **-** | **1.03** | **)** |
| **Infection/inflammation of internal prosthetic device, implant or graft** | **580** | **avg_bmi** | **0.068** | **1.12** | **(** | **0.99** | **-** | **1.26** | **)** |
| **Anorexia** | **225** | **avg_bmi** | **0.068** | **1.19** | **(** | **0.99** | **-** | **1.44** | **)** |
| **Primary pulmonary hypertension** | **146** | **avg_bmi** | **0.068** | **1.24** | **(** | **0.98** | **-** | **1.57** | **)** |
| **Bacteremia** | **683** | **avg_bmi** | **0.068** | **1.11** | **(** | **0.99** | **-** | **1.24** | **)** |
| **Graves' disease** | **105** | **avg_bmi** | **0.069** | **0.76** | **(** | **0.57** | **-** | **1.02** | **)** |
| **Urticaria** | **116** | **avg_bmi** | **0.070** | **0.78** | **(** | **0.59** | **-** | **1.02** | **)** |
| **Malignant neoplasm of small intestine** | **39** | **avg_bmi** | **0.070** | **1.51** | **(** | **0.97** | **-** | **2.35** | **)** |
| **Other disorders of arteries and arterioles** | **127** | **avg_bmi** | **0.071** | **0.79** | **(** | **0.61** | **-** | **1.02** | **)** |
| **Disturbance of salivary secretion** | **38** | **avg_bmi** | **0.072** | **1.51** | **(** | **0.96** | **-** | **2.37** | **)** |
| **Septicemia** | **1202** | **avg_bmi** | **0.072** | **1.08** | **(** | **0.99** | **-** | **1.18** | **)** |
| **Secondary/extrinsic cardiomyopathies** | **280** | **avg_bmi** | **0.074** | **1.17** | **(** | **0.98** | **-** | **1.38** | **)** |
| **Other nonmalignant breast conditions** | **511** | **avg_bmi** | **0.074** | **0.89** | **(** | **0.78** | **-** | **1.01** | **)** |
| **Gestational diabetes** | **62** | **avg_bmi** | **0.075** | **1.41** | **(** | **0.97** | **-** | **2.07** | **)** |
| **Nephritis & nephropathy** | **124** | **avg_bmi** | **0.076** | **1.26** | **(** | **0.98** | **-** | **1.63** | **)** |
| **Diseases of esophagus** | **3410** | **avg_bmi** | **0.078** | **1.06** | **(** | **0.99** | **-** | **1.13** | **)** |
| **Secondary diabetes mellitus** | **58** | **avg_bmi** | **0.078** | **0.70** | **(** | **0.47** | **-** | **1.04** | **)** |
| **Diverticulosis and diverticulitis** | **1257** | **avg_bmi** | **0.080** | **1.08** | **(** | **0.99** | **-** | **1.19** | **)** |
| **Disorders of fluid, electrolyte, and acid-base balance** | **4326** | **avg_bmi** | **0.082** | **1.05** | **(** | **0.99** | **-** | **1.12** | **)** |
| **Respiratory failure; insufficiency; arrest** | **1499** | **avg_bmi** | **0.083** | **1.07** | **(** | **0.99** | **-** | **1.16** | **)** |
| **Essential hypertension** | **8431** | **avg_bmi** | **0.083** | **1.08** | **(** | **0.99** | **-** | **1.17** | **)** |
| **Infections involving bone** | **350** | **avg_bmi** | **0.083** | **1.14** | **(** | **0.98** | **-** | **1.33** | **)** |
| **Abnormal findings on mammogram or breast exam** | **1322** | **avg_bmi** | **0.083** | **0.92** | **(** | **0.85** | **-** | **1.01** | **)** |
| **Chronic fatigue syndrome** | **34** | **avg_bmi** | **0.084** | **1.53** | **(** | **0.95** | **-** | **2.46** | **)** |
| **Hypersomnia** | **34** | **avg_bmi** | **0.084** | **0.63** | **(** | **0.37** | **-** | **1.06** | **)** |
| **Other specified nonpsychotic and/or transient mental disorders** | **53** | **avg_bmi** | **0.084** | **0.69** | **(** | **0.46** | **-** | **1.05** | **)** |
| **Duodenal ulcer** | **70** | **avg_bmi** | **0.086** | **1.34** | **(** | **0.96** | **-** | **1.86** | **)** |
| **Aneurysm of artery of lower extremity** | **54** | **avg_bmi** | **0.086** | **0.70** | **(** | **0.47** | **-** | **1.05** | **)** |
| **Cyst and pseudocyst of pancreas** | **81** | **avg_bmi** | **0.086** | **0.75** | **(** | **0.54** | **-** | **1.04** | **)** |
| **Nasal polyps** | **62** | **avg_bmi** | **0.087** | **1.36** | **(** | **0.96** | **-** | **1.94** | **)** |
| **Lump or mass in breast** | **542** | **avg_bmi** | **0.087** | **0.89** | **(** | **0.78** | **-** | **1.02** | **)** |
| **Systemic sclerosis** | **50** | **avg_bmi** | **0.087** | **0.69** | **(** | **0.45** | **-** | **1.06** | **)** |
| **Other disorders of the kidney and ureters** | **1572** | **avg_bmi** | **0.089** | **1.07** | **(** | **0.99** | **-** | **1.16** | **)** |
| **Disorders of diaphragm** | **39** | **avg_bmi** | **0.090** | **1.47** | **(** | **0.94** | **-** | **2.29** | **)** |
| **Sleep related movement disorders** | **159** | **avg_bmi** | **0.091** | **0.82** | **(** | **0.65** | **-** | **1.03** | **)** |
| **Eye infection, viral** | **5468** | **avg_bmi** | **0.092** | **1.06** | **(** | **0.99** | **-** | **1.13** | **)** |
| **Thrombocytopenia** | **843** | **avg_bmi** | **0.092** | **0.92** | **(** | **0.83** | **-** | **1.01** | **)** |
| **Other specified diseases of nail** | **32** | **avg_bmi** | **0.092** | **1.53** | **(** | **0.93** | **-** | **2.49** | **)** |
| **Malignant neoplasm of gallbladder & extrahepatic bile ducts** | **40** | **avg_bmi** | **0.093** | **1.46** | **(** | **0.94** | **-** | **2.27** | **)** |
| **Cardiac complications, not elsewhere classified** | **69** | **avg_bmi** | **0.093** | **1.33** | **(** | **0.95** | **-** | **1.87** | **)** |
| **Heart failure NOS** | **616** | **avg_bmi** | **0.093** | **1.11** | **(** | **0.98** | **-** | **1.25** | **)** |
| **Schizophrenia and other psychotic disorders** | **257** | **avg_bmi** | **0.093** | **0.86** | **(** | **0.71** | **-** | **1.03** | **)** |
| **Varicose veins of lower extremity, symptomtic** | **126** | **avg_bmi** | **0.094** | **1.24** | **(** | **0.96** | **-** | **1.59** | **)** |
| **Elevated prostate specific antigen** | **457** | **avg_bmi** | **0.095** | **1.13** | **(** | **0.98** | **-** | **1.29** | **)** |
| **Osteomyelitis** | **334** | **avg_bmi** | **0.095** | **1.14** | **(** | **0.98** | **-** | **1.34** | **)** |
| **Psychogenic and somatoform disorders** | **73** | **avg_bmi** | **0.097** | **1.32** | **(** | **0.95** | **-** | **1.84** | **)** |
| **Hypoventilation** | **65** | **avg_bmi** | **0.097** | **1.34** | **(** | **0.95** | **-** | **1.89** | **)** |
| **Nonrheumatic mitral valve disorders** | **908** | **avg_bmi** | **0.098** | **1.09** | **(** | **0.98** | **-** | **1.20** | **)** |
| **Abnormal electrocardiogram** | **552** | **avg_bmi** | **0.099** | **1.11** | **(** | **0.98** | **-** | **1.26** | **)** |
| **Carditis** | **331** | **avg_bmi** | **0.102** | **1.14** | **(** | **0.97** | **-** | **1.33** | **)** |
| **Diverticulum of esophagus, acquired** | **26** | **avg_bmi** | **0.105** | **0.61** | **(** | **0.34** | **-** | **1.11** | **)** |
| **Cardiac arrest & ventricular fibrillation** | **164** | **avg_bmi** | **0.105** | **1.20** | **(** | **0.96** | **-** | **1.49** | **)** |
| **Mental disorders due to brain damage** | **31** | **avg_bmi** | **0.106** | **0.63** | **(** | **0.36** | **-** | **1.10** | **)** |
| **Osteoarthrosis; localized, primary** | **812** | **avg_bmi** | **0.106** | **0.92** | **(** | **0.82** | **-** | **1.02** | **)** |
| **Osteoarthrosis, generalized** | **763** | **avg_bmi** | **0.106** | **0.91** | **(** | **0.82** | **-** | **1.02** | **)** |
| **Gross hematuria** | **98** | **avg_bmi** | **0.107** | **0.78** | **(** | **0.58** | **-** | **1.05** | **)** |
| **Abnormal movement** | **1085** | **avg_bmi** | **0.107** | **1.08** | **(** | **0.98** | **-** | **1.18** | **)** |
| **Immunity deficiency** | **278** | **avg_bmi** | **0.109** | **0.87** | **(** | **0.73** | **-** | **1.03** | **)** |
| **Cancer of other lymphoid, histiocytic tissue** | **563** | **avg_bmi** | **0.110** | **0.90** | **(** | **0.80** | **-** | **1.02** | **)** |
| **Chorioretinal scars** | **36** | **avg_bmi** | **0.111** | **0.67** | **(** | **0.40** | **-** | **1.10** | **)** |
| **Neoplasm of uncertain behavior of breast** | **61** | **avg_bmi** | **0.113** | **0.73** | **(** | **0.50** | **-** | **1.08** | **)** |
| **Other infectious diseases** | **45** | **avg_bmi** | **0.114** | **1.40** | **(** | **0.92** | **-** | **2.11** | **)** |
| **Impacted cerumen** | **482** | **avg_bmi** | **0.114** | **1.11** | **(** | **0.97** | **-** | **1.27** | **)** |
| **Iron metabolism disorder** | **56** | **avg_bmi** | **0.114** | **0.73** | **(** | **0.49** | **-** | **1.08** | **)** |
| **Hyperosmolality and/or hypernatremia** | **135** | **avg_bmi** | **0.115** | **1.21** | **(** | **0.95** | **-** | **1.55** | **)** |
| **Loss of teeth or edentulism** | **41** | **avg_bmi** | **0.115** | **0.68** | **(** | **0.43** | **-** | **1.10** | **)** |
| **Kyphosis (acquired)** | **49** | **avg_bmi** | **0.117** | **1.38** | **(** | **0.92** | **-** | **2.05** | **)** |
| **Orthostatic hypotension** | **254** | **avg_bmi** | **0.117** | **1.15** | **(** | **0.96** | **-** | **1.38** | **)** |
| **Herpes zoster** | **238** | **avg_bmi** | **0.119** | **1.16** | **(** | **0.96** | **-** | **1.39** | **)** |
| **Disorders of esophageal motility** | **59** | **avg_bmi** | **0.121** | **1.33** | **(** | **0.93** | **-** | **1.91** | **)** |
| **Celiac or tropical sprue** | **24** | **avg_bmi** | **0.121** | **1.56** | **(** | **0.89** | **-** | **2.75** | **)** |
| **Celiac disease** | **24** | **avg_bmi** | **0.122** | **1.56** | **(** | **0.89** | **-** | **2.75** | **)** |
| **Intestinal malabsorption** | **74** | **avg_bmi** | **0.122** | **1.29** | **(** | **0.93** | **-** | **1.79** | **)** |
| **Gastrointestinal hemorrhage** | **1410** | **avg_bmi** | **0.122** | **1.07** | **(** | **0.98** | **-** | **1.16** | **)** |
| **Other specified anomalies of kidney** | **23** | **avg_bmi** | **0.125** | **0.60** | **(** | **0.32** | **-** | **1.15** | **)** |
| **Hyperparathyroidism** | **192** | **avg_bmi** | **0.127** | **0.85** | **(** | **0.69** | **-** | **1.05** | **)** |
| **Fever of unknown origin** | **2409** | **avg_bmi** | **0.127** | **1.05** | **(** | **0.99** | **-** | **1.12** | **)** |
| **Seborrheic keratosis** | **1305** | **avg_bmi** | **0.128** | **0.94** | **(** | **0.86** | **-** | **1.02** | **)** |
| **Dementia with cerebral degenerations** | **35** | **avg_bmi** | **0.128** | **0.67** | **(** | **0.41** | **-** | **1.12** | **)** |
| **Psoriasis vulgaris** | **201** | **avg_bmi** | **0.129** | **0.85** | **(** | **0.69** | **-** | **1.05** | **)** |
| **Other nonspecific findings on examination of urine** | **129** | **avg_bmi** | **0.129** | **0.82** | **(** | **0.63** | **-** | **1.06** | **)** |
| **Non-Hodgkins lymphoma** | **559** | **avg_bmi** | **0.130** | **0.91** | **(** | **0.80** | **-** | **1.03** | **)** |
| **Macular puckering of retina** | **128** | **avg_bmi** | **0.130** | **1.21** | **(** | **0.95** | **-** | **1.55** | **)** |
| **Esophagitis, GERD and related diseases** | **3082** | **avg_bmi** | **0.130** | **1.05** | **(** | **0.99** | **-** | **1.12** | **)** |
| **Cellulitis and abscess of oral soft tissues** | **21** | **avg_bmi** | **0.132** | **0.60** | **(** | **0.31** | **-** | **1.17** | **)** |
| **Heart failure** | **2251** | **avg_bmi** | **0.132** | **1.06** | **(** | **0.98** | **-** | **1.13** | **)** |
| **Fracture of foot** | **213** | **avg_bmi** | **0.132** | **1.16** | **(** | **0.96** | **-** | **1.41** | **)** |
| **Convulsions** | **607** | **avg_bmi** | **0.133** | **1.10** | **(** | **0.97** | **-** | **1.23** | **)** |
| **Alkalosis** | **96** | **avg_bmi** | **0.134** | **1.24** | **(** | **0.94** | **-** | **1.65** | **)** |
| **Type 2 diabetic nephropathy** | **696** | **avg_bmi** | **0.134** | **1.09** | **(** | **0.97** | **-** | **1.22** | **)** |
| **Lung cancer** | **372** | **avg_bmi** | **0.135** | **1.12** | **(** | **0.97** | **-** | **1.30** | **)** |
| **Retinal detachment with retinal defect** | **67** | **avg_bmi** | **0.136** | **0.76** | **(** | **0.53** | **-** | **1.09** | **)** |
| **Other paralytic syndromes** | **111** | **avg_bmi** | **0.136** | **1.23** | **(** | **0.94** | **-** | **1.61** | **)** |
| **Type 1 diabetes** | **1056** | **avg_bmi** | **0.138** | **1.07** | **(** | **0.98** | **-** | **1.18** | **)** |
| **Acne** | **147** | **avg_bmi** | **0.138** | **0.83** | **(** | **0.64** | **-** | **1.06** | **)** |
| **Breast cancer, including in situ** | **501** | **avg_bmi** | **0.139** | **0.90** | **(** | **0.79** | **-** | **1.03** | **)** |
| **Cancer of other male genital organs** | **33** | **avg_bmi** | **0.139** | **0.68** | **(** | **0.40** | **-** | **1.14** | **)** |
| **Blindness and low vision** | **169** | **avg_bmi** | **0.140** | **1.18** | **(** | **0.95** | **-** | **1.46** | **)** |
| **Diseases of nail** | **87** | **avg_bmi** | **0.140** | **1.25** | **(** | **0.93** | **-** | **1.70** | **)** |
| **Other biliary tract disease** | **290** | **avg_bmi** | **0.143** | **1.13** | **(** | **0.96** | **-** | **1.34** | **)** |
| **Abnormal findings examination of lungs** | **455** | **avg_bmi** | **0.143** | **0.90** | **(** | **0.79** | **-** | **1.04** | **)** |
| **Occlusion of cerebral arteries, with cerebral infarction** | **105** | **avg_bmi** | **0.144** | **0.81** | **(** | **0.61** | **-** | **1.08** | **)** |
| **Non-melanoma skin cancer** | **1214** | **avg_bmi** | **0.145** | **1.07** | **(** | **0.98** | **-** | **1.17** | **)** |
| **Polycythemia vera, secondary** | **68** | **avg_bmi** | **0.145** | **0.77** | **(** | **0.54** | **-** | **1.10** | **)** |
| **Swelling of limb** | **615** | **avg_bmi** | **0.146** | **1.09** | **(** | **0.97** | **-** | **1.23** | **)** |
| **Arthropod-borne diseases** | **158** | **avg_bmi** | **0.147** | **0.84** | **(** | **0.67** | **-** | **1.06** | **)** |
| **Dermatophytosis of the body** | **59** | **avg_bmi** | **0.147** | **1.31** | **(** | **0.91** | **-** | **1.88** | **)** |
| **Hypothyroidism** | **2378** | **avg_bmi** | **0.147** | **1.05** | **(** | **0.98** | **-** | **1.12** | **)** |
| **Abnormal findings on exam of gastrointestinal tract/abdominal area** | **177** | **avg_bmi** | **0.147** | **0.85** | **(** | **0.68** | **-** | **1.06** | **)** |
| **Unspecified erythematous condition** | **59** | **avg_bmi** | **0.148** | **1.31** | **(** | **0.91** | **-** | **1.88** | **)** |
| **Premature menopause and other ovarian failure** | **44** | **avg_bmi** | **0.148** | **0.72** | **(** | **0.46** | **-** | **1.13** | **)** |
| **Large cell lymphoma** | **186** | **avg_bmi** | **0.149** | **0.86** | **(** | **0.69** | **-** | **1.06** | **)** |
| **Gastritis and duodenitis, NOS** | **44** | **avg_bmi** | **0.150** | **1.36** | **(** | **0.89** | **-** | **2.07** | **)** |
| **Abnormal thyroid function** | **40** | **avg_bmi** | **0.151** | **0.71** | **(** | **0.44** | **-** | **1.14** | **)** |
| **Pain in limb** | **3206** | **avg_bmi** | **0.151** | **1.04** | **(** | **0.98** | **-** | **1.11** | **)** |
| **Develomental delays and disorders** | **123** | **avg_bmi** | **0.154** | **0.83** | **(** | **0.64** | **-** | **1.07** | **)** |
| **Diverticulosis** | **1085** | **avg_bmi** | **0.154** | **1.07** | **(** | **0.97** | **-** | **1.18** | **)** |
| **Type 2 diabetic neuropathy** | **677** | **avg_bmi** | **0.155** | **1.09** | **(** | **0.97** | **-** | **1.22** | **)** |
| **Superficial cellulitis and abscess** | **1563** | **avg_bmi** | **0.155** | **1.06** | **(** | **0.98** | **-** | **1.14** | **)** |
| **Acidosis** | **391** | **avg_bmi** | **0.158** | **1.11** | **(** | **0.96** | **-** | **1.29** | **)** |
| **Angina pectoris** | **673** | **avg_bmi** | **0.159** | **1.09** | **(** | **0.97** | **-** | **1.22** | **)** |
| **Nerve root and plexus disorders** | **84** | **avg_bmi** | **0.159** | **1.24** | **(** | **0.92** | **-** | **1.69** | **)** |
| **Fracture of humerus** | **172** | **avg_bmi** | **0.160** | **1.17** | **(** | **0.94** | **-** | **1.44** | **)** |
| **Ankylosing spondylitis** | **39** | **avg_bmi** | **0.161** | **1.38** | **(** | **0.88** | **-** | **2.17** | **)** |
| **Sleep apnea** | **942** | **avg_bmi** | **0.161** | **1.07** | **(** | **0.97** | **-** | **1.19** | **)** |
| **Disorders of mineral metabolism** | **1024** | **avg_bmi** | **0.161** | **0.94** | **(** | **0.85** | **-** | **1.03** | **)** |
| **Methicillin sensitive Staphylococcus aureus** | **305** | **avg_bmi** | **0.162** | **1.12** | **(** | **0.95** | **-** | **1.32** | **)** |
| **Spontaneous ecchymoses** | **24** | **avg_bmi** | **0.163** | **1.50** | **(** | **0.85** | **-** | **2.64** | **)** |
| **Myoneural disorders** | **46** | **avg_bmi** | **0.163** | **1.34** | **(** | **0.89** | **-** | **2.01** | **)** |
| **Type 1 diabetic neuropathy** | **194** | **avg_bmi** | **0.164** | **1.16** | **(** | **0.94** | **-** | **1.42** | **)** |
| **Retinal detachments and defects** | **125** | **avg_bmi** | **0.165** | **0.83** | **(** | **0.64** | **-** | **1.08** | **)** |
| **Chronic laryngitis** | **99** | **avg_bmi** | **0.165** | **1.22** | **(** | **0.92** | **-** | **1.62** | **)** |
| **Dermatomyositis and Polymyositis** | **37** | **avg_bmi** | **0.166** | **0.71** | **(** | **0.43** | **-** | **1.15** | **)** |
| **Failure to thrive** | **270** | **avg_bmi** | **0.166** | **1.13** | **(** | **0.95** | **-** | **1.35** | **)** |
| **Myeloproliferative disease** | **240** | **avg_bmi** | **0.167** | **0.88** | **(** | **0.73** | **-** | **1.06** | **)** |
| **Hypertension** | **8551** | **avg_bmi** | **0.167** | **1.06** | **(** | **0.98** | **-** | **1.15** | **)** |
| **Benign neoplasm of lip, oral cavity, and pharynx** | **90** | **avg_bmi** | **0.169** | **0.81** | **(** | **0.59** | **-** | **1.10** | **)** |
| **Obesity** | **1345** | **no_bmi** | **1.36E-06** | **1.22** | **(** | **1.13** | **-** | **1.33** | **)** |
| **Overweight** | **1587** | **no_bmi** | **3.22E-05** | **1.18** | **(** | **1.09** | **-** | **1.27** | **)** |
| **Type 2 diabetes** | **3306** | **no_bmi** | **5.26E-05** | **1.14** | **(** | **1.07** | **-** | **1.21** | **)** |
| **Type 2 diabetic ketoacidosis** | **1639** | **no_bmi** | **8.45E-05** | **1.17** | **(** | **1.08** | **-** | **1.26** | **)** |
| **Morbid obesity** | **439** | **no_bmi** | **1.44E-04** | **1.30** | **(** | **1.14** | **-** | **1.49** | **)** |
| **Diabetes mellitus** | **3957** | **no_bmi** | **3.01E-04** | **1.12** | **(** | **1.05** | **-** | **1.18** | **)** |
| **Joint effusions** | **195** | **no_bmi** | **2.24E-03** | **1.37** | **(** | **1.12** | **-** | **1.67** | **)** |
| **Myalgia and myositis NOS** | **729** | **no_bmi** | **2.38E-03** | **1.18** | **(** | **1.06** | **-** | **1.32** | **)** |
| **Sleep apnea** | **942** | **no_bmi** | **4.62E-03** | **1.15** | **(** | **1.04** | **-** | **1.26** | **)** |
| **Rosacea** | **225** | **no_bmi** | **4.94E-03** | **0.75** | **(** | **0.62** | **-** | **0.92** | **)** |
| **Essential hypertension** | **8431** | **no_bmi** | **4.98E-03** | **1.12** | **(** | **1.04** | **-** | **1.21** | **)** |
| **Obstruction of bile duct** | **111** | **no_bmi** | **5.09E-03** | **1.46** | **(** | **1.12** | **-** | **1.90** | **)** |
| **Diseases of pulp and periapical tissues** | **49** | **no_bmi** | **5.26E-03** | **0.52** | **(** | **0.33** | **-** | **0.82** | **)** |
| **Symptoms and disorders of the joints** | **660** | **no_bmi** | **5.60E-03** | **1.17** | **(** | **1.05** | **-** | **1.31** | **)** |
| **Vascular hamartomas and non-neoplastic nevi** | **69** | **no_bmi** | **5.91E-03** | **0.59** | **(** | **0.41** | **-** | **0.86** | **)** |
| **Chronic nonalcoholic liver disease** | **463** | **no_bmi** | **6.06E-03** | **1.20** | **(** | **1.05** | **-** | **1.38** | **)** |
| **Streptococcus infection** | **341** | **no_bmi** | **6.08E-03** | **1.24** | **(** | **1.06** | **-** | **1.44** | **)** |
| **Staphylococcus infections** | **621** | **no_bmi** | **6.39E-03** | **1.17** | **(** | **1.05** | **-** | **1.32** | **)** |
| **Cystoid macular degeneration of retina** | **111** | **no_bmi** | **6.75E-03** | **1.44** | **(** | **1.11** | **-** | **1.87** | **)** |
| **Benign mammary dysplasias** | **526** | **no_bmi** | **6.94E-03** | **0.83** | **(** | **0.73** | **-** | **0.95** | **)** |
| **Pervasive developmental disorders** | **51** | **no_bmi** | **7.16E-03** | **0.54** | **(** | **0.35** | **-** | **0.85** | **)** |
| **Periapical abscess** | **48** | **no_bmi** | **7.75E-03** | **0.54** | **(** | **0.34** | **-** | **0.85** | **)** |
| **Attention deficit hyperactivity disorder** | **43** | **no_bmi** | **8.38E-03** | **0.52** | **(** | **0.32** | **-** | **0.85** | **)** |
| **Abnormal mammogram** | **870** | **no_bmi** | **8.62E-03** | **0.87** | **(** | **0.78** | **-** | **0.96** | **)** |
| **Acute osteomyelitis** | **105** | **no_bmi** | **9.59E-03** | **1.44** | **(** | **1.09** | **-** | **1.89** | **)** |
| **Decreased white blood cell count** | **634** | **no_bmi** | **0.010** | **1.16** | **(** | **1.04** | **-** | **1.31** | **)** |
| **Proteinuria** | **352** | **no_bmi** | **0.011** | **0.82** | **(** | **0.70** | **-** | **0.95** | **)** |
| **Benign neoplasm of thyroid glands** | **37** | **no_bmi** | **0.011** | **1.81** | **(** | **1.14** | **-** | **2.87** | **)** |
| **Acute bronchitis and bronchiolitis** | **772** | **no_bmi** | **0.011** | **1.15** | **(** | **1.03** | **-** | **1.27** | **)** |
| **Cystic mastopathy** | **449** | **no_bmi** | **0.012** | **0.83** | **(** | **0.72** | **-** | **0.96** | **)** |
| **Anterior pituitary disorders** | **96** | **no_bmi** | **0.013** | **0.68** | **(** | **0.50** | **-** | **0.92** | **)** |
| **Localized adiposity** | **36** | **no_bmi** | **0.013** | **1.81** | **(** | **1.13** | **-** | **2.88** | **)** |
| **Acute renal failure** | **1742** | **no_bmi** | **0.013** | **1.10** | **(** | **1.02** | **-** | **1.19** | **)** |
| **Inguinal hernia** | **349** | **no_bmi** | **0.013** | **0.82** | **(** | **0.70** | **-** | **0.96** | **)** |
| **Edema** | **1796** | **no_bmi** | **0.013** | **1.10** | **(** | **1.02** | **-** | **1.18** | **)** |
| **Hypertension** | **8551** | **no_bmi** | **0.014** | **1.11** | **(** | **1.02** | **-** | **1.20** | **)** |
| **Premature beats** | **216** | **no_bmi** | **0.015** | **1.27** | **(** | **1.05** | **-** | **1.54** | **)** |
| **Gram positive septicemia** | **133** | **no_bmi** | **0.015** | **1.35** | **(** | **1.06** | **-** | **1.72** | **)** |
| **Retinal vascular changes and abnomalities** | **125** | **no_bmi** | **0.017** | **1.36** | **(** | **1.06** | **-** | **1.74** | **)** |
| **Hemorrhage from gastrointestinal ulcer** | **61** | **no_bmi** | **0.017** | **1.54** | **(** | **1.08** | **-** | **2.19** | **)** |
| **Cancer of the digestive organs and peritoneum** | **115** | **no_bmi** | **0.018** | **1.37** | **(** | **1.06** | **-** | **1.78** | **)** |
| **Inflammatory disease of breast** | **41** | **no_bmi** | **0.018** | **1.70** | **(** | **1.10** | **-** | **2.63** | **)** |
| **Chronic venous insufficiency** | **206** | **no_bmi** | **0.018** | **1.27** | **(** | **1.04** | **-** | **1.54** | **)** |
| **Mitral stenosis/insufficiency** | **61** | **no_bmi** | **0.018** | **1.54** | **(** | **1.08** | **-** | **2.20** | **)** |
| **Mitral valve stenosis and/or aortic valve stenosis** | **157** | **no_bmi** | **0.018** | **1.31** | **(** | **1.05** | **-** | **1.64** | **)** |
| **Cirrhosis of liver without mention of alcohol** | **342** | **no_bmi** | **0.019** | **1.20** | **(** | **1.03** | **-** | **1.40** | **)** |
| **Nevus, non-neoplastic** | **54** | **no_bmi** | **0.019** | **0.61** | **(** | **0.40** | **-** | **0.92** | **)** |
| **Acute pericarditis** | **33** | **no_bmi** | **0.019** | **1.79** | **(** | **1.10** | **-** | **2.91** | **)** |
| **Nontoxic uninodular goiter** | **216** | **no_bmi** | **0.019** | **1.26** | **(** | **1.04** | **-** | **1.53** | **)** |
| **Crystal arthropathies** | **41** | **no_bmi** | **0.021** | **1.66** | **(** | **1.08** | **-** | **2.56** | **)** |
| **Ill-defined descriptions and complications of heart disease** | **755** | **no_bmi** | **0.021** | **1.13** | **(** | **1.02** | **-** | **1.26** | **)** |
| **Other symptoms involving abdomen and pelvis** | **619** | **no_bmi** | **0.022** | **0.87** | **(** | **0.77** | **-** | **0.98** | **)** |
| **Chronic liver disease and cirrhosis** | **543** | **no_bmi** | **0.023** | **1.15** | **(** | **1.02** | **-** | **1.31** | **)** |
| **Unspecified osteomyelitis** | **248** | **no_bmi** | **0.023** | **1.23** | **(** | **1.03** | **-** | **1.48** | **)** |
| **Intracerebral hemorrhage** | **127** | **no_bmi** | **0.023** | **1.33** | **(** | **1.04** | **-** | **1.71** | **)** |
| **Other diseases of the teeth and supporting structures** | **79** | **no_bmi** | **0.023** | **0.67** | **(** | **0.48** | **-** | **0.95** | **)** |
| **Raynaud's syndrome** | **77** | **no_bmi** | **0.024** | **0.67** | **(** | **0.48** | **-** | **0.95** | **)** |
| **Respiratory insufficiency** | **901** | **no_bmi** | **0.025** | **1.12** | **(** | **1.01** | **-** | **1.24** | **)** |
| **Myocardial infarction** | **1152** | **no_bmi** | **0.025** | **1.11** | **(** | **1.01** | **-** | **1.21** | **)** |
| **Astigmatism** | **82** | **no_bmi** | **0.026** | **1.42** | **(** | **1.04** | **-** | **1.93** | **)** |
| **Myasthenia gravis** | **41** | **no_bmi** | **0.026** | **1.63** | **(** | **1.06** | **-** | **2.51** | **)** |
| **Benign neoplasm of respiratory and intrathoracic organs** | **70** | **no_bmi** | **0.026** | **0.67** | **(** | **0.46** | **-** | **0.95** | **)** |
| **Infection of the eye** | **5597** | **no_bmi** | **0.026** | **1.08** | **(** | **1.01** | **-** | **1.15** | **)** |
| **Secondary malignant neoplasm of liver** | **249** | **no_bmi** | **0.027** | **1.22** | **(** | **1.02** | **-** | **1.46** | **)** |
| **Lipoma of skin & subcutaneous tissue** | **77** | **no_bmi** | **0.027** | **1.43** | **(** | **1.04** | **-** | **1.96** | **)** |
| **Pericarditis** | **171** | **no_bmi** | **0.027** | **1.27** | **(** | **1.03** | **-** | **1.58** | **)** |
| **Anemia in chronic kidney disease** | **336** | **no_bmi** | **0.027** | **0.83** | **(** | **0.71** | **-** | **0.98** | **)** |
| **Hypoparathyroidism** | **32** | **no_bmi** | **0.028** | **1.74** | **(** | **1.06** | **-** | **2.84** | **)** |
| **Cardiac dysrhythmias** | **4842** | **no_bmi** | **0.028** | **1.07** | **(** | **1.01** | **-** | **1.14** | **)** |
| **Hypoglycemia** | **111** | **no_bmi** | **0.029** | **1.35** | **(** | **1.03** | **-** | **1.76** | **)** |
| **Cancer of connective tissue** | **169** | **no_bmi** | **0.030** | **1.27** | **(** | **1.02** | **-** | **1.57** | **)** |
| **Disease of tricuspid valve** | **123** | **no_bmi** | **0.030** | **1.32** | **(** | **1.03** | **-** | **1.71** | **)** |
| **Neutropenia** | **542** | **no_bmi** | **0.030** | **1.15** | **(** | **1.01** | **-** | **1.30** | **)** |
| **Renal failure** | **2941** | **no_bmi** | **0.030** | **1.07** | **(** | **1.01** | **-** | **1.14** | **)** |
| **Infection/inflammation of internal prosthetic device, implant or graft** | **580** | **no_bmi** | **0.030** | **1.14** | **(** | **1.01** | **-** | **1.29** | **)** |
| **Nontoxic nodular goiter** | **446** | **no_bmi** | **0.033** | **1.16** | **(** | **1.01** | **-** | **1.33** | **)** |
| **Pituitary hypofunction** | **62** | **no_bmi** | **0.033** | **0.66** | **(** | **0.45** | **-** | **0.97** | **)** |
| **Type 2 diabetic nephropathy** | **696** | **no_bmi** | **0.033** | **1.13** | **(** | **1.01** | **-** | **1.26** | **)** |
| **Secondary malignancy of lung** | **352** | **no_bmi** | **0.034** | **1.18** | **(** | **1.01** | **-** | **1.37** | **)** |
| **Diseases of blood and blood-forming organs** | **259** | **no_bmi** | **0.034** | **0.82** | **(** | **0.69** | **-** | **0.99** | **)** |
| **Type 2 diabetic neuropathy** | **677** | **no_bmi** | **0.035** | **1.13** | **(** | **1.01** | **-** | **1.26** | **)** |
| **Peptic ulcer** | **326** | **no_bmi** | **0.037** | **1.18** | **(** | **1.01** | **-** | **1.39** | **)** |
| **Primary angle-closure glaucoma** | **75** | **no_bmi** | **0.037** | **1.41** | **(** | **1.02** | **-** | **1.94** | **)** |
| **Inflammatory conditions of jaw** | **21** | **no_bmi** | **0.037** | **0.47** | **(** | **0.23** | **-** | **0.96** | **)** |
| **Arthropathy NOS involving multiple sites** | **35** | **no_bmi** | **0.038** | **0.57** | **(** | **0.34** | **-** | **0.97** | **)** |
| **Chondrocalcinosis** | **40** | **no_bmi** | **0.038** | **1.59** | **(** | **1.03** | **-** | **2.46** | **)** |
| **Polyarteritis nodosa and allied conditions** | **171** | **no_bmi** | **0.039** | **0.79** | **(** | **0.63** | **-** | **0.99** | **)** |
| **Chronic rheumatic disease of the heart valves** | **330** | **no_bmi** | **0.039** | **1.18** | **(** | **1.01** | **-** | **1.38** | **)** |
| **Malignant neoplasm of renal pelvis** | **45** | **no_bmi** | **0.039** | **1.54** | **(** | **1.02** | **-** | **2.33** | **)** |
| **Renal cell carcinoma** | **256** | **no_bmi** | **0.040** | **1.20** | **(** | **1.01** | **-** | **1.43** | **)** |
| **Giant cell arteritis** | **48** | **no_bmi** | **0.040** | **0.63** | **(** | **0.41** | **-** | **0.98** | **)** |
| **Cancer of kidney and renal pelvis** | **262** | **no_bmi** | **0.041** | **1.20** | **(** | **1.01** | **-** | **1.43** | **)** |
| **Type 1 diabetes** | **1056** | **no_bmi** | **0.041** | **1.10** | **(** | **1.00** | **-** | **1.21** | **)** |
| **Mental retardation** | **56** | **no_bmi** | **0.043** | **0.66** | **(** | **0.44** | **-** | **0.99** | **)** |
| **Open wound of hand except finger(s)** | **61** | **no_bmi** | **0.043** | **0.67** | **(** | **0.46** | **-** | **0.99** | **)** |
| **Disorders of the pituitary gland and its hypothalamic control** | **173** | **no_bmi** | **0.043** | **0.79** | **(** | **0.64** | **-** | **0.99** | **)** |
| **Cancer of other female genital organs** | **41** | **no_bmi** | **0.045** | **1.56** | **(** | **1.01** | **-** | **2.40** | **)** |
| **Diseases of esophagus** | **3410** | **no_bmi** | **0.046** | **1.07** | **(** | **1.00** | **-** | **1.14** | **)** |
| **Breast cancer** | **547** | **no_bmi** | **0.047** | **0.88** | **(** | **0.77** | **-** | **1.00** | **)** |
| **Bacteremia** | **683** | **no_bmi** | **0.047** | **1.12** | **(** | **1.00** | **-** | **1.25** | **)** |
| **Subdural hemorrhage (injury)** | **81** | **no_bmi** | **0.048** | **0.72** | **(** | **0.52** | **-** | **1.00** | **)** |
| **Hemorrhage of gastrointestinal tract** | **535** | **no_bmi** | **0.049** | **1.13** | **(** | **1.00** | **-** | **1.28** | **)** |
| **Neuralgia, neuritis, and radiculitis NOS** | **421** | **no_bmi** | **0.049** | **1.15** | **(** | **1.00** | **-** | **1.32** | **)** |
| **Disorders of tooth development** | **40** | **no_bmi** | **0.051** | **1.55** | **(** | **1.00** | **-** | **2.41** | **)** |
| **Primary pulmonary hypertension** | **146** | **no_bmi** | **0.051** | **1.26** | **(** | **1.00** | **-** | **1.59** | **)** |
| **Heart failure** | **2251** | **no_bmi** | **0.052** | **1.07** | **(** | **1.00** | **-** | **1.15** | **)** |
| **Myoclonus** | **50** | **no_bmi** | **0.052** | **1.48** | **(** | **1.00** | **-** | **2.18** | **)** |
| **Diffuse diseases of connective tissue** | **225** | **no_bmi** | **0.052** | **0.82** | **(** | **0.68** | **-** | **1.00** | **)** |
| **Pancreatic cancer** | **135** | **no_bmi** | **0.052** | **1.27** | **(** | **1.00** | **-** | **1.62** | **)** |
| **Benign neoplasm of uterus** | **187** | **no_bmi** | **0.053** | **0.81** | **(** | **0.65** | **-** | **1.00** | **)** |
| **Cyst and pseudocyst of pancreas** | **81** | **no_bmi** | **0.053** | **0.72** | **(** | **0.52** | **-** | **1.00** | **)** |
| **Non-healing surgical wound** | **78** | **no_bmi** | **0.053** | **0.72** | **(** | **0.51** | **-** | **1.00** | **)** |
| **Infections involving bone** | **350** | **no_bmi** | **0.054** | **1.16** | **(** | **1.00** | **-** | **1.35** | **)** |
| **Squamous cell carcinoma** | **120** | **no_bmi** | **0.054** | **1.28** | **(** | **1.00** | **-** | **1.66** | **)** |
| **Nodular lymphoma** | **169** | **no_bmi** | **0.054** | **0.80** | **(** | **0.64** | **-** | **1.00** | **)** |
| **Disturbances in tooth eruption** | **38** | **no_bmi** | **0.055** | **1.56** | **(** | **0.99** | **-** | **2.45** | **)** |
| **Eye infection, viral** | **5468** | **no_bmi** | **0.055** | **1.06** | **(** | **1.00** | **-** | **1.14** | **)** |
| **Iron deficiency anemia secondary to blood loss** | **345** | **no_bmi** | **0.055** | **1.16** | **(** | **1.00** | **-** | **1.36** | **)** |
| **Urethral hypermobility/ISD** | **78** | **no_bmi** | **0.055** | **0.72** | **(** | **0.52** | **-** | **1.01** | **)** |
| **Peyronie's disease** | **21** | **no_bmi** | **0.056** | **0.51** | **(** | **0.26** | **-** | **1.02** | **)** |
| **Superficial cellulitis and abscess** | **1563** | **no_bmi** | **0.056** | **1.08** | **(** | **1.00** | **-** | **1.16** | **)** |
| **Septicemia** | **1202** | **no_bmi** | **0.057** | **1.09** | **(** | **1.00** | **-** | **1.18** | **)** |
| **Other disorders of the kidney and ureters** | **1572** | **no_bmi** | **0.057** | **1.08** | **(** | **1.00** | **-** | **1.17** | **)** |
| **Nontoxic multinodular goiter** | **287** | **no_bmi** | **0.057** | **1.18** | **(** | **1.00** | **-** | **1.40** | **)** |
| **Gestational diabetes** | **62** | **no_bmi** | **0.061** | **1.44** | **(** | **0.98** | **-** | **2.11** | **)** |
| **Chronic pericarditis** | **42** | **no_bmi** | **0.061** | **1.51** | **(** | **0.98** | **-** | **2.32** | **)** |
| **Prostate cancer** | **512** | **no_bmi** | **0.061** | **0.88** | **(** | **0.77** | **-** | **1.01** | **)** |
| **Respiratory failure; insufficiency; arrest** | **1499** | **no_bmi** | **0.064** | **1.08** | **(** | **1.00** | **-** | **1.17** | **)** |
| **Other specified nonpsychotic and/or transient mental disorders** | **53** | **no_bmi** | **0.064** | **0.68** | **(** | **0.45** | **-** | **1.02** | **)** |
| **Nephritis & nephropathy** | **124** | **no_bmi** | **0.064** | **1.27** | **(** | **0.99** | **-** | **1.64** | **)** |
| **Infusion and transfusion reaction** | **40** | **no_bmi** | **0.065** | **1.51** | **(** | **0.98** | **-** | **2.34** | **)** |
| **Pathologic fracture** | **267** | **no_bmi** | **0.065** | **1.18** | **(** | **0.99** | **-** | **1.40** | **)** |
| **Graves' disease** | **105** | **no_bmi** | **0.065** | **0.76** | **(** | **0.57** | **-** | **1.02** | **)** |
| **Diverticulosis and diverticulitis** | **1257** | **no_bmi** | **0.065** | **1.09** | **(** | **0.99** | **-** | **1.19** | **)** |
| **Osteomyelitis** | **334** | **no_bmi** | **0.066** | **1.16** | **(** | **0.99** | **-** | **1.35** | **)** |
| **Secondary diabetes mellitus** | **58** | **no_bmi** | **0.066** | **0.69** | **(** | **0.46** | **-** | **1.03** | **)** |
| **Obstructive sleep apnea** | **636** | **no_bmi** | **0.067** | **1.11** | **(** | **0.99** | **-** | **1.25** | **)** |
| **Hypersomnia** | **34** | **no_bmi** | **0.069** | **0.62** | **(** | **0.36** | **-** | **1.04** | **)** |
| **Other disorders of adrenal glands** | **36** | **no_bmi** | **0.069** | **0.62** | **(** | **0.38** | **-** | **1.04** | **)** |
| **Other disorders of arteries and arterioles** | **127** | **no_bmi** | **0.069** | **0.79** | **(** | **0.61** | **-** | **1.02** | **)** |
| **Uterine leiomyoma** | **169** | **no_bmi** | **0.069** | **0.81** | **(** | **0.64** | **-** | **1.02** | **)** |
| **Malignant neoplasm of small intestine** | **39** | **no_bmi** | **0.070** | **1.51** | **(** | **0.97** | **-** | **2.35** | **)** |
| **Secondary/extrinsic cardiomyopathies** | **280** | **no_bmi** | **0.070** | **1.17** | **(** | **0.99** | **-** | **1.39** | **)** |
| **Pituitary hyperfunction** | **46** | **no_bmi** | **0.070** | **0.66** | **(** | **0.42** | **-** | **1.03** | **)** |
| **Esophagitis, GERD and related diseases** | **3082** | **no_bmi** | **0.072** | **1.06** | **(** | **0.99** | **-** | **1.13** | **)** |
| **Chronic fatigue syndrome** | **34** | **no_bmi** | **0.073** | **1.55** | **(** | **0.96** | **-** | **2.50** | **)** |
| **Antisocial/borderline personality disorder** | **37** | **no_bmi** | **0.074** | **0.63** | **(** | **0.38** | **-** | **1.05** | **)** |
| **Pain in limb** | **3206** | **no_bmi** | **0.074** | **1.06** | **(** | **0.99** | **-** | **1.12** | **)** |
| **Heart failure NOS** | **616** | **no_bmi** | **0.075** | **1.11** | **(** | **0.99** | **-** | **1.25** | **)** |
| **Systemic sclerosis** | **50** | **no_bmi** | **0.075** | **0.68** | **(** | **0.45** | **-** | **1.04** | **)** |
| **Swelling of limb** | **615** | **no_bmi** | **0.075** | **1.11** | **(** | **0.99** | **-** | **1.25** | **)** |
| **Disorders of fluid, electrolyte, and acid-base balance** | **4326** | **no_bmi** | **0.075** | **1.06** | **(** | **0.99** | **-** | **1.12** | **)** |
| **Disorders of diaphragm** | **39** | **no_bmi** | **0.077** | **1.49** | **(** | **0.96** | **-** | **2.33** | **)** |
| **Cancer of bone & connective tissue** | **241** | **no_bmi** | **0.077** | **1.18** | **(** | **0.98** | **-** | **1.41** | **)** |
| **Torsion dystonia** | **94** | **no_bmi** | **0.077** | **1.30** | **(** | **0.97** | **-** | **1.73** | **)** |
| **Disturbance of salivary secretion** | **38** | **no_bmi** | **0.078** | **1.50** | **(** | **0.96** | **-** | **2.35** | **)** |
| **Cellulitis and abscess of leg** | **492** | **no_bmi** | **0.078** | **1.12** | **(** | **0.99** | **-** | **1.28** | **)** |
| **Duodenal ulcer** | **70** | **no_bmi** | **0.078** | **1.35** | **(** | **0.97** | **-** | **1.87** | **)** |
| **Other nonmalignant breast conditions** | **511** | **no_bmi** | **0.081** | **0.89** | **(** | **0.78** | **-** | **1.01** | **)** |
| **Abnormal findings on mammogram or breast exam** | **1322** | **no_bmi** | **0.081** | **0.92** | **(** | **0.85** | **-** | **1.01** | **)** |
| **Varicose veins of lower extremity, symptomtic** | **126** | **no_bmi** | **0.082** | **1.25** | **(** | **0.97** | **-** | **1.60** | **)** |
| **Disorders of adrenal glands** | **285** | **no_bmi** | **0.084** | **0.86** | **(** | **0.72** | **-** | **1.02** | **)** |
| **Mental disorders due to brain damage** | **31** | **no_bmi** | **0.085** | **0.62** | **(** | **0.36** | **-** | **1.07** | **)** |
| **Cardiac complications, not elsewhere classified** | **69** | **no_bmi** | **0.085** | **1.34** | **(** | **0.96** | **-** | **1.88** | **)** |
| **Schizophrenia and other psychotic disorders** | **257** | **no_bmi** | **0.086** | **0.85** | **(** | **0.71** | **-** | **1.02** | **)** |
| **Cardiac arrest & ventricular fibrillation** | **164** | **no_bmi** | **0.086** | **1.21** | **(** | **0.97** | **-** | **1.51** | **)** |
| **Hypoventilation** | **65** | **no_bmi** | **0.087** | **1.35** | **(** | **0.96** | **-** | **1.91** | **)** |
| **Systolic/diastolic heart failure** | **1965** | **no_bmi** | **0.088** | **1.07** | **(** | **0.99** | **-** | **1.15** | **)** |
| **Abnormal electrocardiogram** | **552** | **no_bmi** | **0.088** | **1.12** | **(** | **0.98** | **-** | **1.26** | **)** |
| **Aneurysm of artery of lower extremity** | **54** | **no_bmi** | **0.089** | **0.70** | **(** | **0.47** | **-** | **1.05** | **)** |
| **Hypothyroidism** | **2378** | **no_bmi** | **0.089** | **1.06** | **(** | **0.99** | **-** | **1.13** | **)** |
| **Lump or mass in breast** | **542** | **no_bmi** | **0.089** | **0.89** | **(** | **0.79** | **-** | **1.02** | **)** |
| **Urticaria** | **116** | **no_bmi** | **0.090** | **0.79** | **(** | **0.60** | **-** | **1.04** | **)** |
| **Nasal polyps** | **62** | **no_bmi** | **0.093** | **1.35** | **(** | **0.95** | **-** | **1.93** | **)** |
| **Throat pain** | **37** | **no_bmi** | **0.093** | **1.48** | **(** | **0.94** | **-** | **2.34** | **)** |
| **Elevated prostate specific antigen** | **457** | **no_bmi** | **0.094** | **1.13** | **(** | **0.98** | **-** | **1.29** | **)** |
| **Psychogenic and somatoform disorders** | **73** | **no_bmi** | **0.096** | **1.32** | **(** | **0.95** | **-** | **1.84** | **)** |
| **Herpes zoster** | **238** | **no_bmi** | **0.096** | **1.17** | **(** | **0.97** | **-** | **1.40** | **)** |
| **Sleep related movement disorders** | **159** | **no_bmi** | **0.098** | **0.82** | **(** | **0.65** | **-** | **1.04** | **)** |
| **Acne** | **147** | **no_bmi** | **0.098** | **0.81** | **(** | **0.63** | **-** | **1.04** | **)** |
| **Alkalosis** | **96** | **no_bmi** | **0.099** | **1.27** | **(** | **0.96** | **-** | **1.69** | **)** |
| **Fracture of foot** | **213** | **no_bmi** | **0.099** | **1.18** | **(** | **0.97** | **-** | **1.43** | **)** |
| **Malignant neoplasm of gallbladder & extrahepatic bile ducts** | **40** | **no_bmi** | **0.100** | **1.45** | **(** | **0.93** | **-** | **2.24** | **)** |
| **Other infectious diseases** | **45** | **no_bmi** | **0.100** | **1.42** | **(** | **0.94** | **-** | **2.14** | **)** |
| **Abnormal involuntary movements** | **291** | **no_bmi** | **0.101** | **1.15** | **(** | **0.97** | **-** | **1.36** | **)** |
| **Other specified diseases of nail** | **32** | **no_bmi** | **0.101** | **1.51** | **(** | **0.92** | **-** | **2.46** | **)** |
| **Diverticulum of esophagus, acquired** | **26** | **no_bmi** | **0.103** | **0.61** | **(** | **0.34** | **-** | **1.10** | **)** |
| **Angina pectoris** | **673** | **no_bmi** | **0.105** | **1.10** | **(** | **0.98** | **-** | **1.23** | **)** |
| **Thrombocytopenia** | **843** | **no_bmi** | **0.106** | **0.92** | **(** | **0.83** | **-** | **1.02** | **)** |
| **Neoplasm of uncertain behavior of breast** | **61** | **no_bmi** | **0.106** | **0.73** | **(** | **0.50** | **-** | **1.07** | **)** |
| **Intestinal malabsorption** | **74** | **no_bmi** | **0.109** | **1.30** | **(** | **0.94** | **-** | **1.80** | **)** |
| **Hyperosmolality and/or hypernatremia** | **135** | **no_bmi** | **0.110** | **1.22** | **(** | **0.96** | **-** | **1.55** | **)** |
| **Cancer of other lymphoid, histiocytic tissue** | **563** | **no_bmi** | **0.111** | **0.90** | **(** | **0.80** | **-** | **1.02** | **)** |
| **Other specified anomalies of kidney** | **23** | **no_bmi** | **0.111** | **0.59** | **(** | **0.31** | **-** | **1.13** | **)** |
| **Abnormal findings examination of lungs** | **455** | **no_bmi** | **0.112** | **0.89** | **(** | **0.78** | **-** | **1.03** | **)** |
| **Seborrheic keratosis** | **1305** | **no_bmi** | **0.114** | **0.93** | **(** | **0.86** | **-** | **1.02** | **)** |
| **Type 1 diabetic neuropathy** | **194** | **no_bmi** | **0.114** | **1.18** | **(** | **0.96** | **-** | **1.45** | **)** |
| **Loss of teeth or edentulism** | **41** | **no_bmi** | **0.115** | **0.68** | **(** | **0.43** | **-** | **1.10** | **)** |
| **Gross hematuria** | **98** | **no_bmi** | **0.115** | **0.79** | **(** | **0.59** | **-** | **1.06** | **)** |
| **Carditis** | **331** | **no_bmi** | **0.116** | **1.13** | **(** | **0.97** | **-** | **1.33** | **)** |
| **Iron metabolism disorder** | **56** | **no_bmi** | **0.117** | **0.73** | **(** | **0.49** | **-** | **1.08** | **)** |
| **Methicillin sensitive Staphylococcus aureus** | **305** | **no_bmi** | **0.120** | **1.14** | **(** | **0.97** | **-** | **1.34** | **)** |
| **Fever of unknown origin** | **2409** | **no_bmi** | **0.121** | **1.05** | **(** | **0.99** | **-** | **1.12** | **)** |
| **Chorioretinal scars** | **36** | **no_bmi** | **0.123** | **0.67** | **(** | **0.41** | **-** | **1.11** | **)** |
| **Hypertensive heart disease** | **867** | **no_bmi** | **0.123** | **1.09** | **(** | **0.98** | **-** | **1.21** | **)** |
| **Disorders of esophageal motility** | **59** | **no_bmi** | **0.124** | **1.33** | **(** | **0.93** | **-** | **1.90** | **)** |
| **Gastrointestinal hemorrhage** | **1410** | **no_bmi** | **0.124** | **1.07** | **(** | **0.98** | **-** | **1.16** | **)** |
| **Unspecified erythematous condition** | **59** | **no_bmi** | **0.124** | **1.33** | **(** | **0.92** | **-** | **1.91** | **)** |
| **Immunity deficiency** | **278** | **no_bmi** | **0.125** | **0.87** | **(** | **0.73** | **-** | **1.04** | **)** |
| **Non-Hodgkins lymphoma** | **559** | **no_bmi** | **0.125** | **0.91** | **(** | **0.80** | **-** | **1.03** | **)** |
| **Osteoporosis, osteopenia, & pathological fractures** | **2986** | **no_bmi** | **0.127** | **0.95** | **(** | **0.89** | **-** | **1.01** | **)** |
| **Macular puckering of retina** | **128** | **no_bmi** | **0.127** | **1.21** | **(** | **0.95** | **-** | **1.55** | **)** |
| **Dementia with cerebral degenerations** | **35** | **no_bmi** | **0.127** | **0.67** | **(** | **0.41** | **-** | **1.12** | **)** |
| **Retinal detachment with retinal defect** | **67** | **no_bmi** | **0.127** | **0.76** | **(** | **0.53** | **-** | **1.08** | **)** |
| **Celiac or tropical sprue** | **24** | **no_bmi** | **0.129** | **1.55** | **(** | **0.88** | **-** | **2.72** | **)** |
| **Celiac disease** | **24** | **no_bmi** | **0.129** | **1.55** | **(** | **0.88** | **-** | **2.72** | **)** |
| **Abnormal thyroid function** | **40** | **no_bmi** | **0.131** | **0.69** | **(** | **0.43** | **-** | **1.12** | **)** |
| **Abnormal findings on exam of gastrointestinal tract/abdominal area** | **177** | **no_bmi** | **0.132** | **0.85** | **(** | **0.68** | **-** | **1.05** | **)** |
| **Dermatophytosis of the body** | **59** | **no_bmi** | **0.132** | **1.32** | **(** | **0.92** | **-** | **1.90** | **)** |
| **Nonrheumatic mitral valve disorders** | **908** | **no_bmi** | **0.133** | **1.08** | **(** | **0.98** | **-** | **1.19** | **)** |
| **Scoliosis** | **145** | **no_bmi** | **0.133** | **0.83** | **(** | **0.65** | **-** | **1.06** | **)** |
| **Nerve root and plexus disorders** | **84** | **no_bmi** | **0.134** | **1.26** | **(** | **0.93** | **-** | **1.71** | **)** |
| **Osteoarthrosis, generalized** | **763** | **no_bmi** | **0.135** | **0.92** | **(** | **0.82** | **-** | **1.03** | **)** |
| **Diverticulosis** | **1085** | **no_bmi** | **0.135** | **1.08** | **(** | **0.98** | **-** | **1.18** | **)** |
| **Blindness and low vision** | **169** | **no_bmi** | **0.135** | **1.18** | **(** | **0.95** | **-** | **1.46** | **)** |
| **Acidosis** | **391** | **no_bmi** | **0.136** | **1.12** | **(** | **0.97** | **-** | **1.29** | **)** |
| **Acid-base balance disorder** | **513** | **no_bmi** | **0.136** | **1.10** | **(** | **0.97** | **-** | **1.25** | **)** |
| **Other nonspecific findings on examination of urine** | **129** | **no_bmi** | **0.136** | **0.82** | **(** | **0.64** | **-** | **1.06** | **)** |
| **Abnormal movement** | **1085** | **no_bmi** | **0.138** | **1.07** | **(** | **0.98** | **-** | **1.17** | **)** |
| **Cellulitis and abscess of oral soft tissues** | **21** | **no_bmi** | **0.139** | **0.60** | **(** | **0.31** | **-** | **1.18** | **)** |
| **GERD** | **2856** | **no_bmi** | **0.140** | **1.05** | **(** | **0.98** | **-** | **1.12** | **)** |
| **Orthostatic hypotension** | **254** | **no_bmi** | **0.140** | **1.14** | **(** | **0.96** | **-** | **1.37** | **)** |
| **Kyphosis (acquired)** | **49** | **no_bmi** | **0.141** | **1.35** | **(** | **0.91** | **-** | **2.00** | **)** |
| **Acquired deformities of ankle and foot** | **167** | **no_bmi** | **0.141** | **1.18** | **(** | **0.95** | **-** | **1.46** | **)** |
| **Hyperparathyroidism** | **192** | **no_bmi** | **0.141** | **0.85** | **(** | **0.69** | **-** | **1.05** | **)** |
| **Spontaneous ecchymoses** | **24** | **no_bmi** | **0.141** | **1.53** | **(** | **0.87** | **-** | **2.70** | **)** |
| **Fracture of humerus** | **172** | **no_bmi** | **0.142** | **1.17** | **(** | **0.95** | **-** | **1.45** | **)** |
| **Myeloproliferative disease** | **240** | **no_bmi** | **0.143** | **0.87** | **(** | **0.72** | **-** | **1.05** | **)** |
| **Premature menopause and other ovarian failure** | **44** | **no_bmi** | **0.143** | **0.71** | **(** | **0.45** | **-** | **1.12** | **)** |
| **Anorexia** | **225** | **no_bmi** | **0.143** | **1.15** | **(** | **0.95** | **-** | **1.39** | **)** |
| **Myoneural disorders** | **46** | **no_bmi** | **0.144** | **1.35** | **(** | **0.90** | **-** | **2.04** | **)** |
| **Lung cancer** | **372** | **no_bmi** | **0.144** | **1.12** | **(** | **0.96** | **-** | **1.29** | **)** |
| **Occlusion of cerebral arteries, with cerebral infarction** | **105** | **no_bmi** | **0.145** | **0.81** | **(** | **0.61** | **-** | **1.08** | **)** |
| **HIV infection, symptomatic** | **58** | **no_bmi** | **0.145** | **0.75** | **(** | **0.51** | **-** | **1.10** | **)** |
| **Cancer of other male genital organs** | **33** | **no_bmi** | **0.146** | **0.68** | **(** | **0.40** | **-** | **1.14** | **)** |
| **Human immunodeficiency virus** | **58** | **no_bmi** | **0.146** | **0.75** | **(** | **0.51** | **-** | **1.11** | **)** |
| **Ventral hernia** | **188** | **no_bmi** | **0.147** | **1.16** | **(** | **0.95** | **-** | **1.43** | **)** |
| **Other forms of chronic heart disease** | **740** | **no_bmi** | **0.148** | **1.08** | **(** | **0.97** | **-** | **1.21** | **)** |
| **Polycythemia vera, secondary** | **68** | **no_bmi** | **0.148** | **0.77** | **(** | **0.54** | **-** | **1.10** | **)** |
| **Chronic ulcer of unspecified site** | **119** | **no_bmi** | **0.150** | **1.21** | **(** | **0.93** | **-** | **1.56** | **)** |

**†** Values are not corrected for multiple testing

## Supplementary Table 2: Top 100 phenotypes from the meta-analysis of SNP rs6499640, in low association with rs8050136. The table is a detailed description of the meta-analysis PheWAS analysis of rs6499640 for both populations (n=24,198). The columns state the number of cases, the adjustment (either by average BMI (avg_bmi) or no adjustment (no_bmi), the raw p-value, the Odds Ratio (OR), and the 95% Confidence Interval (95% CI). The Bonferroni alpha=0.05 equates to a p-value of 4.95x10^-5^, and an FDR of q=0.05 gives a p-value of 2.48x10^-4^.

| **rs6499640 - eMERGE Population** | | | | | | | | | |
| --- | --- | --- | --- | --- | --- | --- | --- | --- | --- |
|  |  |  |  |  |  |  |  |  |  |
| **PheWAS description** | **Cases** | **adjustment** | **p†** | **OR** | **95% CI** | | | | |
| **Dysphagia** | **1861** | **avg_bmi** | **1.51E-03** | **1.12** | **(** | **1.05** | **-** | **1.21** | **)** |
| **Other disorders of gallbladder** | **77** | **avg_bmi** | **1.71E-03** | **1.66** | **(** | **1.21** | **-** | **2.28** | **)** |
| **Symptoms and disorders of the joints** | **1386** | **avg_bmi** | **3.82E-03** | **1.13** | **(** | **1.04** | **-** | **1.23** | **)** |
| **GERD** | **4422** | **avg_bmi** | **5.56E-03** | **1.08** | **(** | **1.02** | **-** | **1.14** | **)** |
| **Esophagitis, GERD and related diseases** | **5368** | **avg_bmi** | **6.08E-03** | **1.07** | **(** | **1.02** | **-** | **1.13** | **)** |
| **Benign neoplasm of other endocrine glands** | **184** | **avg_bmi** | **9.82E-03** | **1.31** | **(** | **1.07** | **-** | **1.62** | **)** |
| **Disorders of the autonomic nervous system** | **226** | **avg_bmi** | **0.011** | **1.28** | **(** | **1.06** | **-** | **1.54** | **)** |
| **Primary thrombocytopenia** | **109** | **avg_bmi** | **0.013** | **1.41** | **(** | **1.08** | **-** | **1.84** | **)** |
| **Iatrogenic hypotension** | **75** | **avg_bmi** | **0.013** | **0.64** | **(** | **0.45** | **-** | **0.91** | **)** |
| **Dysthymic disorder** | **713** | **avg_bmi** | **0.014** | **1.15** | **(** | **1.03** | **-** | **1.28** | **)** |
| **Intervertebral disc disorders** | **2593** | **avg_bmi** | **0.017** | **1.08** | **(** | **1.01** | **-** | **1.15** | **)** |
| **Enthesopathy** | **2334** | **avg_bmi** | **0.019** | **1.09** | **(** | **1.01** | **-** | **1.17** | **)** |
| **Rosacea** | **511** | **avg_bmi** | **0.020** | **1.16** | **(** | **1.02** | **-** | **1.32** | **)** |
| **Bladder cancer and neoplasms** | **425** | **avg_bmi** | **0.022** | **1.18** | **(** | **1.02** | **-** | **1.35** | **)** |
| **Other biliary tract disease** | **500** | **avg_bmi** | **0.023** | **1.16** | **(** | **1.02** | **-** | **1.32** | **)** |
| **Discoid lupus erythematosus** | **270** | **avg_bmi** | **0.024** | **0.81** | **(** | **0.68** | **-** | **0.97** | **)** |
| **Diseases of esophagus** | **5908** | **avg_bmi** | **0.025** | **1.06** | **(** | **1.01** | **-** | **1.11** | **)** |
| **Agorophobia, social phobia, and panic disorder** | **193** | **avg_bmi** | **0.025** | **1.26** | **(** | **1.03** | **-** | **1.55** | **)** |
| **Spinal stenosis of lumbar region** | **1114** | **avg_bmi** | **0.025** | **1.11** | **(** | **1.01** | **-** | **1.21** | **)** |
| **Otitis media** | **731** | **avg_bmi** | **0.027** | **1.13** | **(** | **1.01** | **-** | **1.26** | **)** |
| **Other abnormality of urination** | **665** | **avg_bmi** | **0.027** | **0.88** | **(** | **0.78** | **-** | **0.99** | **)** |
| **Eustachian tube disorders** | **1282** | **avg_bmi** | **0.028** | **1.1** | **(** | **1.01** | **-** | **1.20** | **)** |
| **Chronic ulcer of unspecified site** | **242** | **avg_bmi** | **0.028** | **0.81** | **(** | **0.67** | **-** | **0.98** | **)** |
| **Senile cataract** | **4138** | **avg_bmi** | **0.028** | **0.94** | **(** | **0.88** | **-** | **0.99** | **)** |
| **Spinal stenosis** | **1302** | **avg_bmi** | **0.032** | **1.09** | **(** | **1.01** | **-** | **1.19** | **)** |
| **Nausea and vomiting** | **3369** | **avg_bmi** | **0.033** | **1.06** | **(** | **1.00** | **-** | **1.12** | **)** |
| **Sarcoidosis** | **86** | **avg_bmi** | **0.033** | **1.39** | **(** | **1.03** | **-** | **1.88** | **)** |
| **Patellar fracture** | **105** | **avg_bmi** | **0.036** | **0.73** | **(** | **0.55** | **-** | **0.98** | **)** |
| **Eustachian tube disorders** | **445** | **avg_bmi** | **0.036** | **1.16** | **(** | **1.01** | **-** | **1.32** | **)** |
| **Superficial keratitis** | **95** | **avg_bmi** | **0.036** | **0.72** | **(** | **0.52** | **-** | **0.98** | **)** |
| **Osteoarthritis; localized** | **2525** | **avg_bmi** | **0.040** | **1.07** | **(** | **1.00** | **-** | **1.14** | **)** |
| **Nonrheumatic aortic valve disorders** | **1262** | **avg_bmi** | **0.041** | **1.09** | **(** | **1.00** | **-** | **1.19** | **)** |
| **Other conditions of brain** | **460** | **avg_bmi** | **0.043** | **1.15** | **(** | **1.00** | **-** | **1.32** | **)** |
| **Intracerebral hemorrhage** | **154** | **avg_bmi** | **0.044** | **1.27** | **(** | **1.01** | **-** | **1.60** | **)** |
| **Bladder cancer** | **401** | **avg_bmi** | **0.045** | **1.16** | **(** | **1.00** | **-** | **1.34** | **)** |
| **Hemorrhage from gastrointestinal ulcer** | **101** | **avg_bmi** | **0.046** | **0.73** | **(** | **0.54** | **-** | **0.99** | **)** |
| **Abnormal thyroid function** | **116** | **avg_bmi** | **0.047** | **1.3** | **(** | **1.00** | **-** | **1.69** | **)** |
| **Anemia in chronic kidney disease** | **411** | **avg_bmi** | **0.047** | **1.15** | **(** | **1.00** | **-** | **1.33** | **)** |
| **Uveitis** | **171** | **avg_bmi** | **0.048** | **0.79** | **(** | **0.63** | **-** | **1.00** | **)** |
| **Keratitis** | **492** | **avg_bmi** | **0.053** | **0.87** | **(** | **0.76** | **-** | **1.00** | **)** |
| **Cystoid macular degeneration of retina** | **209** | **avg_bmi** | **0.053** | **0.82** | **(** | **0.67** | **-** | **1.00** | **)** |
| **Chronic pain syndrome** | **108** | **avg_bmi** | **0.053** | **1.31** | **(** | **1.00** | **-** | **1.71** | **)** |
| **Fracture of vertebral column without mention of spinal cord injury** | **579** | **avg_bmi** | **0.055** | **0.89** | **(** | **0.78** | **-** | **1.00** | **)** |
| **Hereditary and idiopathic peripheral neuropathy** | **1115** | **avg_bmi** | **0.056** | **1.09** | **(** | **1.00** | **-** | **1.19** | **)** |
| **Polycythemia vera** | **97** | **avg_bmi** | **0.056** | **1.32** | **(** | **0.99** | **-** | **1.76** | **)** |
| **Effects of radiation NOS** | **100** | **avg_bmi** | **0.056** | **1.32** | **(** | **0.99** | **-** | **1.75** | **)** |
| **Cyst of kidney, acquired** | **323** | **avg_bmi** | **0.057** | **0.85** | **(** | **0.72** | **-** | **1.00** | **)** |
| **Endometrial hyperplasia** | **72** | **avg_bmi** | **0.057** | **0.71** | **(** | **0.49** | **-** | **1.01** | **)** |
| **Other open wound of head and face** | **267** | **avg_bmi** | **0.062** | **1.18** | **(** | **0.99** | **-** | **1.41** | **)** |
| **Hemoptysis** | **295** | **avg_bmi** | **0.065** | **1.17** | **(** | **0.99** | **-** | **1.38** | **)** |
| **Dysphagia** | **1861** | **no_bmi** | **1.14E-03** | **1.13** | **(** | **1.05** | **-** | **1.21** | **)** |
| **Other disorders of gallbladder** | **77** | **no_bmi** | **1.73E-03** | **1.66** | **(** | **1.21** | **-** | **2.28** | **)** |
| **Symptoms and disorders of the joints** | **1386** | **no_bmi** | **6.16E-03** | **1.12** | **(** | **1.03** | **-** | **1.22** | **)** |
| **GERD** | **4422** | **no_bmi** | **9.03E-03** | **1.08** | **(** | **1.02** | **-** | **1.14** | **)** |
| **Disorders of the autonomic nervous system** | **226** | **no_bmi** | **9.51E-03** | **1.28** | **(** | **1.06** | **-** | **1.55** | **)** |
| **Primary thrombocytopenia** | **109** | **no_bmi** | **0.011** | **1.41** | **(** | **1.08** | **-** | **1.85** | **)** |
| **Esophagitis, GERD and related diseases** | **5368** | **no_bmi** | **0.011** | **1.07** | **(** | **1.02** | **-** | **1.12** | **)** |
| **Iatrogenic hypotension** | **75** | **no_bmi** | **0.013** | **0.64** | **(** | **0.45** | **-** | **0.91** | **)** |
| **Benign neoplasm of other endocrine glands** | **184** | **no_bmi** | **0.014** | **1.3** | **(** | **1.05** | **-** | **1.60** | **)** |
| **Dysthymic disorder** | **713** | **no_bmi** | **0.015** | **1.15** | **(** | **1.03** | **-** | **1.28** | **)** |
| **Other biliary tract disease** | **500** | **no_bmi** | **0.021** | **1.16** | **(** | **1.02** | **-** | **1.32** | **)** |
| **Bladder cancer and neoplasms** | **425** | **no_bmi** | **0.021** | **1.18** | **(** | **1.02** | **-** | **1.36** | **)** |
| **Rosacea** | **511** | **no_bmi** | **0.022** | **1.16** | **(** | **1.02** | **-** | **1.32** | **)** |
| **Intervertebral disc disorders** | **2593** | **no_bmi** | **0.024** | **1.07** | **(** | **1.01** | **-** | **1.14** | **)** |
| **Chronic ulcer of unspecified site** | **242** | **no_bmi** | **0.024** | **0.8** | **(** | **0.66** | **-** | **0.97** | **)** |
| **Enthesopathy** | **2334** | **no_bmi** | **0.025** | **1.09** | **(** | **1.01** | **-** | **1.17** | **)** |
| **Senile cataract** | **4138** | **no_bmi** | **0.027** | **0.94** | **(** | **0.88** | **-** | **0.99** | **)** |
| **Other abnormality of urination** | **665** | **no_bmi** | **0.027** | **0.88** | **(** | **0.78** | **-** | **0.99** | **)** |
| **Agorophobia, social phobia, and panic disorder** | **193** | **no_bmi** | **0.028** | **1.26** | **(** | **1.03** | **-** | **1.54** | **)** |
| **Discoid lupus erythematosus** | **270** | **no_bmi** | **0.030** | **0.82** | **(** | **0.68** | **-** | **0.98** | **)** |
| **Superficial keratitis** | **95** | **no_bmi** | **0.032** | **0.71** | **(** | **0.52** | **-** | **0.97** | **)** |
| **Nausea and vomiting** | **3369** | **no_bmi** | **0.034** | **1.06** | **(** | **1.00** | **-** | **1.12** | **)** |
| **Eustachian tube disorders** | **1282** | **no_bmi** | **0.037** | **1.09** | **(** | **1.01** | **-** | **1.19** | **)** |
| **Diseases of esophagus** | **5908** | **no_bmi** | **0.037** | **1.05** | **(** | **1.00** | **-** | **1.11** | **)** |
| **Spinal stenosis of lumbar region** | **1114** | **no_bmi** | **0.037** | **1.1** | **(** | **1.01** | **-** | **1.20** | **)** |
| **Patellar fracture** | **105** | **no_bmi** | **0.038** | **0.73** | **(** | **0.55** | **-** | **0.98** | **)** |
| **Otitis media** | **731** | **no_bmi** | **0.038** | **1.12** | **(** | **1.01** | **-** | **1.25** | **)** |
| **Eustachian tube disorders** | **445** | **no_bmi** | **0.040** | **1.15** | **(** | **1.01** | **-** | **1.32** | **)** |
| **Hemorrhage from gastrointestinal ulcer** | **101** | **no_bmi** | **0.041** | **0.73** | **(** | **0.54** | **-** | **0.99** | **)** |
| **Nonrheumatic aortic valve disorders** | **1262** | **no_bmi** | **0.041** | **1.09** | **(** | **1.00** | **-** | **1.19** | **)** |
| **Spinal stenosis** | **1302** | **no_bmi** | **0.044** | **1.09** | **(** | **1.00** | **-** | **1.18** | **)** |
| **Intracerebral hemorrhage** | **154** | **no_bmi** | **0.044** | **1.27** | **(** | **1.01** | **-** | **1.59** | **)** |
| **Abnormal thyroid function** | **116** | **no_bmi** | **0.044** | **1.31** | **(** | **1.01** | **-** | **1.70** | **)** |
| **Other conditions of brain** | **460** | **no_bmi** | **0.045** | **1.15** | **(** | **1.00** | **-** | **1.32** | **)** |
| **Bladder cancer** | **401** | **no_bmi** | **0.045** | **1.16** | **(** | **1.00** | **-** | **1.34** | **)** |
| **Sarcoidosis** | **86** | **no_bmi** | **0.046** | **1.36** | **(** | **1.01** | **-** | **1.83** | **)** |
| **Uveitis** | **171** | **no_bmi** | **0.047** | **0.79** | **(** | **0.63** | **-** | **1.00** | **)** |
| **Endometrial hyperplasia** | **72** | **no_bmi** | **0.049** | **0.7** | **(** | **0.49** | **-** | **1.00** | **)** |
| **Cystoid macular degeneration of retina** | **209** | **no_bmi** | **0.051** | **0.82** | **(** | **0.67** | **-** | **1.00** | **)** |
| **Cyst of kidney, acquired** | **323** | **no_bmi** | **0.052** | **0.85** | **(** | **0.72** | **-** | **1.00** | **)** |
| **Polycythemia vera** | **97** | **no_bmi** | **0.054** | **1.32** | **(** | **1.00** | **-** | **1.76** | **)** |
| **Other open wound of head and face** | **267** | **no_bmi** | **0.055** | **1.19** | **(** | **1.00** | **-** | **1.42** | **)** |
| **Hemoptysis** | **295** | **no_bmi** | **0.056** | **1.18** | **(** | **1.00** | **-** | **1.39** | **)** |
| **Anemia in chronic kidney disease** | **411** | **no_bmi** | **0.057** | **1.15** | **(** | **1.00** | **-** | **1.32** | **)** |
| **Effects of radiation NOS** | **100** | **no_bmi** | **0.057** | **1.32** | **(** | **0.99** | **-** | **1.75** | **)** |
| **Keratitis** | **492** | **no_bmi** | **0.060** | **0.88** | **(** | **0.77** | **-** | **1.01** | **)** |
| **Fracture of vertebral column without mention of spinal cord injury** | **579** | **no_bmi** | **0.063** | **0.89** | **(** | **0.79** | **-** | **1.01** | **)** |
| **Osteoarthritis; localized** | **2525** | **no_bmi** | **0.065** | **1.06** | **(** | **1.00** | **-** | **1.14** | **)** |
| **Chronic pain syndrome** | **108** | **no_bmi** | **0.066** | **1.29** | **(** | **0.98** | **-** | **1.69** | **)** |
| **Hereditary and idiopathic peripheral neuropathy** | **1115** | **no_bmi** | **0.067** | **1.09** | **(** | **0.99** | **-** | **1.19** | **)** |

**†** Values are not corrected for multiple testing

## Supplementary Table 3: Results from the top 100 phenotypes from the eMERGE PheWAS of rs7199182, in low association with rs8050136. The table is a detailed description of the meta-analysis PheWAS analysis of rs7199182 for both populations (n=24,198). The columns state the number of cases, the adjustment (either by average BMI (avg_bmi) or no adjustment (no_bmi), the raw p-value, the Odds Ratio (OR), and the 95% Confidence Interval (95% CI). The Bonferroni alpha=0.05 equates to a p-value of 4.95x10^-5^, and an FDR of q=0.05 gives a p-value of 2.48x10^-4^.

| **rs7199182 - eMERGE Population** | | | | | | | | | |
| --- | --- | --- | --- | --- | --- | --- | --- | --- | --- |
|  |  |  |  |  |  |  |  |  |  |
| **PheWAS description** | **Cases** | **adjustment** | **p†** | **OR** | **95% CI** | | | | |
| **Chronic periodontitis** | **202** | **avg_bmi** | **5.20E-05** | **14.66** | **(** | **3.99** | **-** | **53.8** | **)** |
| **Periodontitis (acute or chronic)** | **227** | **avg_bmi** | **6.45E-05** | **12.09** | **(** | **3.56** | **-** | **41.1** | **)** |
| **Gingival and periodontal diseases** | **273** | **avg_bmi** | **1.98E-04** | **8.55** | **(** | **2.76** | **-** | **26.5** | **)** |
| **Urinary obstruction** | **40** | **avg_bmi** | **5.18E-04** | **57.75** | **(** | **5.85** | **-** | **570** | **)** |
| **Arthropathy NOS involving multiple sites** | **25** | **avg_bmi** | **8.31E-04** | **101.37** | **(** | **6.75** | **-** | **1522** | **)** |
| **Type 2 diabetic retinopathy** | **30** | **avg_bmi** | **3.88E-03** | **28.99** | **(** | **2.95** | **-** | **285** | **)** |
| **Paroxysmal ventricular tachycardia** | **103** | **avg_bmi** | **6.77E-03** | **9.24** | **(** | **1.85** | **-** | **46.2** | **)** |
| **Attention deficit hyperactivity disorder** | **33** | **avg_bmi** | **6.91E-03** | **19.23** | **(** | **2.25** | **-** | **164** | **)** |
| **Pervasive developmental disorders** | **37** | **avg_bmi** | **9.06E-03** | **17.01** | **(** | **2.02** | **-** | **143** | **)** |
| **Hypertrophy of breast (Gynecomastia)** | **70** | **avg_bmi** | **9.17E-03** | **7.09** | **(** | **1.62** | **-** | **30.9** | **)** |
| **Paroxysmal tachycardia, unspecified** | **142** | **avg_bmi** | **9.71E-03** | **8.08** | **(** | **1.66** | **-** | **39.4** | **)** |
| **Viral Enteritis** | **31** | **avg_bmi** | **0.010** | **16.53** | **(** | **1.94** | **-** | **141** | **)** |
| **Inflammatory spondylopathies** | **37** | **avg_bmi** | **0.012** | **15.58** | **(** | **1.84** | **-** | **132** | **)** |
| **Pallor and flushing** | **53** | **avg_bmi** | **0.012** | **16.22** | **(** | **1.85** | **-** | **142** | **)** |
| **Cancer of the upper aerodigestive tract** | **49** | **avg_bmi** | **0.012** | **16.31** | **(** | **1.85** | **-** | **144** | **)** |
| **Congenital deformities of feet** | **22** | **avg_bmi** | **0.012** | **21.55** | **(** | **1.96** | **-** | **237** | **)** |
| **Congenital anomalies of peripheral vascular system** | **27** | **avg_bmi** | **0.012** | **15.11** | **(** | **1.8** | **-** | **127** | **)** |
| **Cerebral aneurysm** | **32** | **avg_bmi** | **0.013** | **15.19** | **(** | **1.77** | **-** | **131** | **)** |
| **Breast conditions, congenital or relating to hormones** | **86** | **avg_bmi** | **0.015** | **6.02** | **(** | **1.42** | **-** | **25.5** | **)** |
| **Acquired spondylolisthesis** | **123** | **avg_bmi** | **0.016** | **7.05** | **(** | **1.43** | **-** | **34.7** | **)** |
| **Urethral hypermobility/ISD** | **30** | **avg_bmi** | **0.017** | **13.65** | **(** | **1.61** | **-** | **116** | **)** |
| **Cervicocranial/Cervicobrachial syndrome** | **45** | **avg_bmi** | **0.019** | **39.71** | **(** | **1.83** | **-** | **861** | **)** |
| **Degeneration of intervertebral disc** | **746** | **avg_bmi** | **0.020** | **3.15** | **(** | **1.2** | **-** | **8.29** | **)** |
| **Early complications of trauma or procedure** | **24** | **avg_bmi** | **0.023** | **13.76** | **(** | **1.44** | **-** | **131** | **)** |
| **Peripheral angiopathy in diseases classified elsewhere** | **38** | **avg_bmi** | **0.024** | **11.92** | **(** | **1.38** | **-** | **103** | **)** |
| **Secondary/extrinsic cardiomyopathies** | **36** | **avg_bmi** | **0.025** | **11.13** | **(** | **1.34** | **-** | **92.1** | **)** |
| **Other spec nonpsychotic/transient mental disorder** | **166** | **avg_bmi** | **0.028** | **6.19** | **(** | **1.22** | **-** | **31.3** | **)** |
| **Other nutritional deficiency** | **23** | **avg_bmi** | **0.029** | **11.12** | **(** | **1.28** | **-** | **96.6** | **)** |
| **Methicillin sensitive Staphylococcus aureus** | **41** | **avg_bmi** | **0.032** | **9.98** | **(** | **1.22** | **-** | **81.4** | **)** |
| **Psychogenic disorder** | **32** | **avg_bmi** | **0.033** | **12.05** | **(** | **1.23** | **-** | **119** | **)** |
| **Chronic fatigue syndrome** | **26** | **avg_bmi** | **0.033** | **11.11** | **(** | **1.22** | **-** | **102** | **)** |
| **Intracerebral hemorrhage** | **27** | **avg_bmi** | **0.033** | **11.42** | **(** | **1.21** | **-** | **107** | **)** |
| **Anisometropia** | **99** | **avg_bmi** | **0.033** | **18.37** | **(** | **1.26** | **-** | **268** | **)** |
| **Conduct disorders** | **22** | **avg_bmi** | **0.034** | **15.26** | **(** | **1.23** | **-** | **189** | **)** |
| **Congenital anomalies of limbs** | **38** | **avg_bmi** | **0.034** | **11.49** | **(** | **1.2** | **-** | **110** | **)** |
| **Other acquired musculoskeletal deformity** | **167** | **avg_bmi** | **0.035** | **5.42** | **(** | **1.13** | **-** | **26** | **)** |
| **Hypovolemia** | **551** | **avg_bmi** | **0.035** | **3.5** | **(** | **1.09** | **-** | **11.2** | **)** |
| **Astigmatism** | **1401** | **avg_bmi** | **0.038** | **4.67** | **(** | **1.09** | **-** | **20** | **)** |
| **Immunity deficiency** | **26** | **avg_bmi** | **0.038** | **9.97** | **(** | **1.14** | **-** | **87.3** | **)** |
| **Open wound of hand except finger(s)** | **87** | **avg_bmi** | **0.039** | **9.37** | **(** | **1.11** | **-** | **78.8** | **)** |
| **Symptoms affecting skin** | **490** | **avg_bmi** | **0.040** | **3.13** | **(** | **1.05** | **-** | **9.27** | **)** |
| **H. pylori** | **36** | **avg_bmi** | **0.050** | **8.44** | **(** | **1** | **-** | **71** | **)** |
| **Chronic ischemic heart disease** | **2097** | **avg_bmi** | **0.050** | **0.27** | **(** | **0.07** | **-** | **1** | **)** |
| **Other disorders of intestine** | **1301** | **avg_bmi** | **0.051** | **0.08** | **(** | **0.01** | **-** | **1.01** | **)** |
| **Gastrointestinal complications** | **942** | **avg_bmi** | **0.063** | **0.05** | **(** | **0** | **-** | **1.17** | **)** |
| **Tuberculosis** | **65** | **avg_bmi** | **0.065** | **7.79** | **(** | **0.88** | **-** | **69.1** | **)** |
| **Osteoporosis, NOS or other** | **139** | **avg_bmi** | **0.066** | **4.54** | **(** | **0.91** | **-** | **22.7** | **)** |
| **Speech and language disorder** | **45** | **avg_bmi** | **0.066** | **8.72** | **(** | **0.87** | **-** | **87.6** | **)** |
| **Other specified osteoporosis** | **31** | **avg_bmi** | **0.073** | **7.82** | **(** | **0.83** | **-** | **74.1** | **)** |
| **Sleep apnea** | **1393** | **avg_bmi** | **0.132** | **0.19** | **(** | **0.02** | **-** | **1.66** | **)** |
| **Chronic periodontitis** | **202** | **no_bmi** | **5.40E-05** | **14.58** | **(** | **3.97** | **-** | **53.6** | **)** |
| **Periodontitis (acute or chronic)** | **227** | **no_bmi** | **7.23E-05** | **11.95** | **(** | **3.51** | **-** | **40.7** | **)** |
| **Gingival and periodontal diseases** | **273** | **no_bmi** | **2.14E-04** | **8.5** | **(** | **2.74** | **-** | **26.4** | **)** |
| **Urinary obstruction** | **40** | **no_bmi** | **6.48E-04** | **53.16** | **(** | **5.42** | **-** | **521** | **)** |
| **Arthropathy NOS involving multiple sites** | **25** | **no_bmi** | **6.81E-04** | **109.94** | **(** | **7.3** | **-** | **1655** | **)** |
| **Type 2 diabetic retinopathy** | **30** | **no_bmi** | **4.73E-03** | **27.87** | **(** | **2.77** | **-** | **280** | **)** |
| **Cancer of the upper aerodigestive tract** | **49** | **no_bmi** | **5.83E-03** | **19.96** | **(** | **2.38** | **-** | **168** | **)** |
| **Attention deficit hyperactivity disorder** | **33** | **no_bmi** | **6.66E-03** | **19.24** | **(** | **2.27** | **-** | **163** | **)** |
| **Paroxysmal ventricular tachycardia** | **103** | **no_bmi** | **8.47E-03** | **8.62** | **(** | **1.73** | **-** | **42.9** | **)** |
| **Pervasive developmental disorders** | **37** | **no_bmi** | **8.67E-03** | **17.13** | **(** | **2.05** | **-** | **143** | **)** |
| **Congenital deformities of feet** | **22** | **no_bmi** | **9.12E-03** | **23.26** | **(** | **2.18** | **-** | **248** | **)** |
| **Paroxysmal tachycardia, unspecified** | **142** | **no_bmi** | **9.23E-03** | **8.16** | **(** | **1.68** | **-** | **39.7** | **)** |
| **Congenital anomalies of peripheral vascular system** | **27** | **no_bmi** | **9.44E-03** | **16.72** | **(** | **1.99** | **-** | **140** | **)** |
| **Hypertrophy of breast (Gynecomastia)** | **70** | **no_bmi** | **0.010** | **6.91** | **(** | **1.58** | **-** | **30.2** | **)** |
| **Inflammatory spondylopathies** | **37** | **no_bmi** | **0.011** | **15.85** | **(** | **1.89** | **-** | **133** | **)** |
| **Cerebral aneurysm** | **32** | **no_bmi** | **0.012** | **15.73** | **(** | **1.86** | **-** | **133** | **)** |
| **Pallor and flushing** | **53** | **no_bmi** | **0.012** | **16.22** | **(** | **1.85** | **-** | **142** | **)** |
| **Viral Enteritis** | **31** | **no_bmi** | **0.013** | **15.41** | **(** | **1.8** | **-** | **132** | **)** |
| **Acquired spondylolisthesis** | **123** | **no_bmi** | **0.016** | **7.1** | **(** | **1.45** | **-** | **34.9** | **)** |
| **Urethral hypermobility/ISD** | **30** | **no_bmi** | **0.017** | **13.63** | **(** | **1.61** | **-** | **116** | **)** |
| **Breast conditions, congenital or relating to hormones** | **86** | **no_bmi** | **0.018** | **5.77** | **(** | **1.36** | **-** | **24.5** | **)** |
| **Other spec nonpsychotic/transient mental disorder** | **166** | **no_bmi** | **0.020** | **6.77** | **(** | **1.36** | **-** | **33.7** | **)** |
| **Gastrointestinal complications** | **942** | **no_bmi** | **0.023** | **0.07** | **(** | **0.01** | **-** | **0.69** | **)** |
| **Intracerebral hemorrhage** | **27** | **no_bmi** | **0.024** | **12.95** | **(** | **1.39** | **-** | **120** | **)** |
| **Anisometropia** | **99** | **no_bmi** | **0.024** | **19.69** | **(** | **1.47** | **-** | **264** | **)** |
| **Early complications of trauma or procedure** | **24** | **no_bmi** | **0.025** | **13.07** | **(** | **1.38** | **-** | **124** | **)** |
| **Secondary/extrinsic cardiomyopathies** | **36** | **no_bmi** | **0.026** | **11.05** | **(** | **1.34** | **-** | **91.3** | **)** |
| **Degeneration of intervertebral disc** | **746** | **no_bmi** | **0.026** | **2.99** | **(** | **1.14** | **-** | **7.83** | **)** |
| **Psychogenic disorder** | **32** | **no_bmi** | **0.026** | **13.15** | **(** | **1.35** | **-** | **128** | **)** |
| **Peripheral angiopathy in diseases classified elsewhere** | **38** | **no_bmi** | **0.027** | **11.35** | **(** | **1.32** | **-** | **97.8** | **)** |
| **Conduct disorders** | **22** | **no_bmi** | **0.029** | **16.96** | **(** | **1.35** | **-** | **214** | **)** |
| **Other acquired musculoskeletal deformity** | **167** | **no_bmi** | **0.030** | **5.63** | **(** | **1.19** | **-** | **26.7** | **)** |
| **Hypovolemia** | **551** | **no_bmi** | **0.031** | **3.59** | **(** | **1.13** | **-** | **11.5** | **)** |
| **Chronic fatigue syndrome** | **26** | **no_bmi** | **0.031** | **11.31** | **(** | **1.25** | **-** | **102** | **)** |
| **Osteoporosis, NOS or other** | **139** | **no_bmi** | **0.031** | **5.7** | **(** | **1.18** | **-** | **27.7** | **)** |
| **Congenital anomalies of limbs** | **38** | **no_bmi** | **0.031** | **11.74** | **(** | **1.25** | **-** | **111** | **)** |
| **Cervicocranial/Cervicobrachial syndrome** | **45** | **no_bmi** | **0.032** | **33.06** | **(** | **1.36** | **-** | **802** | **)** |
| **Immunity deficiency** | **26** | **no_bmi** | **0.033** | **10.37** | **(** | **1.21** | **-** | **88.8** | **)** |
| **Other disorders of intestine** | **1301** | **no_bmi** | **0.033** | **0.1** | **(** | **0.01** | **-** | **0.83** | **)** |
| **Methicillin sensitive Staphylococcus aureus** | **41** | **no_bmi** | **0.034** | **9.63** | **(** | **1.18** | **-** | **78.6** | **)** |
| **Other nutritional deficiency** | **23** | **no_bmi** | **0.035** | **10.24** | **(** | **1.18** | **-** | **88.7** | **)** |
| **Astigmatism** | **1401** | **no_bmi** | **0.036** | **4.72** | **(** | **1.1** | **-** | **20.1** | **)** |
| **Open wound of hand except finger(s)** | **87** | **no_bmi** | **0.041** | **9.2** | **(** | **1.09** | **-** | **77.4** | **)** |
| **Symptoms affecting skin** | **490** | **no_bmi** | **0.042** | **3.08** | **(** | **1.04** | **-** | **9.14** | **)** |
| **Chronic ischemic heart disease** | **2097** | **no_bmi** | **0.044** | **0.25** | **(** | **0.07** | **-** | **0.96** | **)** |
| **Sleep apnea** | **1393** | **no_bmi** | **0.047** | **0.11** | **(** | **0.01** | **-** | **0.97** | **)** |
| **Speech and language disorder** | **45** | **no_bmi** | **0.048** | **10.02** | **(** | **1.03** | **-** | **98** | **)** |
| **Tuberculosis** | **65** | **no_bmi** | **0.048** | **8.88** | **(** | **1.02** | **-** | **77** | **)** |
| **Other specified osteoporosis** | **31** | **no_bmi** | **0.048** | **9.05** | **(** | **1.02** | **-** | **80.4** | **)** |
| **H. pylori** | **36** | **no_bmi** | **0.049** | **8.5** | **(** | **1.01** | **-** | **71.2** | **)** |

**†** Values are not corrected for multiple testing
